# Cytotoxic Agents by the Phosphinoylation and Thiophosphinoylation of 3‑Hydroxy-1,2,3,6-tetrahydrophosphinine 1‑Oxides as β‑Hydroxyphosphonates and -Phosphine Oxides

**DOI:** 10.1021/acsomega.5c11546

**Published:** 2026-02-26

**Authors:** Zsuzsanna Szalai, Kristóf Szloboda, Konstantin Karaghiosoff, Mátyás Czugler, Angéla Takács, László Kőhidai, Ágnes Gömöry, László Drahos, György Keglevich

**Affiliations:** † Department of Organic Chemistry and Technology, Faculty of Chemical Technology and Biotechnology, 61810 Budapest University of Technology and Economics, Műegyetem rkp. 3, 1111 Budapest, Hungary; ‡ Department Chemie, Ludwig-Maximilians-Universität München, Butenandtstr. 5-13, D-81377 München, Germany; § Department of Genetics, Cell and Immunobiology, Semmelweis University, Nagyvárad tér 4, 1089 Budapest, Hungary; ∥ MS Proteomics Research Group, 280964 Research Centre for Natural Sciences, 1117 Budapest, Hungary

## Abstract

A series of hydroxy-1,2,3,6-tetrahydrophosphinine oxides were prepared by the two-step ring enlargement of 1-substituted 3-phospholene 1-oxides via the corresponding dichlorocarbene adducts. The two diastereomers of the *P*-ethoxy-3-phosphabicyclo­[3.1.0]­hexane 3-oxides could be identified by single crystal X-ray analysis, hence the isomers could be characterized by NMR methods. Detailed examination of the crystal structures of the two isomers shows weak O···H and Cl···H interactions, which are different for the two isomers, in accord with the different arrangements of the molecules in the solid state. The less hindered hydroxy-tetrahydrophosphinine oxide isomers were selectively phosphinoylated and thiophosphinoylated. The cytotoxic effect of the P-heterocycles synthesized was tested on U266 myeloma cells and on A2058 melanoma cells. The results are promising, as the viability of the cells was decreased drastically at the higher 100 μM concentration, especially in respect of one hydroxy-tetrahydrophosphinine oxide and the two P-functionalized derivatives, independently of the substituent’s nature.

## Graphical abstract

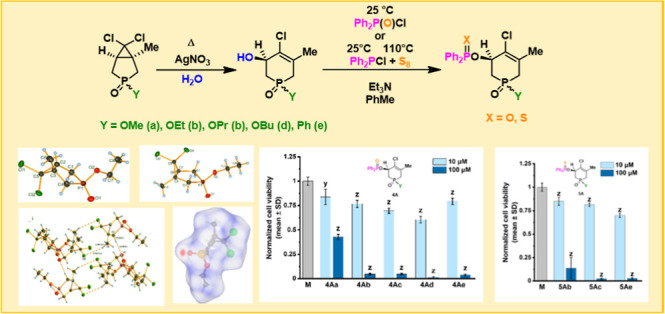

## 1 Introduction

Hydroxyphosphonates in general form a representative group within phosphonates. No doubt that the α-hydroxyphosphonates should be regarded the most prominent representatives, whose synthesis and reactivity are still in the focus^1^ due to the green chemical challenges^2,3^ and the potential bioactivity.^4–8^ Dronic acid derivatives are α-substituted-α-hydroxy-methylene-bisphosphonic acid derivatives^9^ that are applied in the treatment of bone diseases.^10–13^ It was our experience that the modification of the hydroxy group of α-hydroxyphosphonates by acylation^14^ or phosphorylation^15,16^ may lead to the increase in the bioactivity meaning cytotoxic activity. Furthermore, modification of α-hydroxy-methylene-bisphosphonates by acylation or rearrangement led also to promising species regarding cytotoxicity.^17–19^ The synthesis and chemistry of β-hydroxyphosphonates is a less explored field.^20–24^ There were some interesting bioactivities reported. The β-hydroxyphosphonate analogues of biotin-5′-AMP are inhibitors of the holocarboxylase synthetise enzyme.^25^ It was also found that β-hydroxyphosphonate derivatives as autotaxin (ATX) inhibitors are attractive drug targets, as they may slow the spread of cancer.^26^ Another observation was that the β-hydroxyphosphonate analogues of l-carnitine decrease the level of glucose, triglicerides and cholesterol in the liver, and the level of triglicerides in the serum.^27^ A β-hydroxy-gamma-aminophosphonate increased the metabolic activity of *Nocardia brasiliensis* associated with the increased hydrolysis of the substrates tested, such as l-tyrosine.^28^

It was a challenge for us to modify 3-hydroxy-1,2,3,6-tetrahydrophosphinine oxides described by us earlier.^29^ These suitably functionalized P-heterocycles are a matter of fact, special β-hydroxyphosphonates. It was a challenge for us to convert them to the phosphinoylated and thiophosphinoylated derivatives and to test them as cytotoxic agents on myeloma and other cell lines.

## 2 Results and Discussion

### 2.1 Preparation of 3-Hydroxy-1,2,3,6-tetrahydrophosphinine 1-Oxide Starting Materials as β-Hydroxyphosphonates and as a β-Hydroxyphosphine Oxide

The title compounds, the hydroxy-tetrahydrophosphinine oxides (**3a**–**e**) were prepared by the two-step ring enlargement of the 3-phospholene oxides (**1a**–**e**) elaborated by the Keglevich group earlier.^30–32^ According to this, dichlorocarbene generated from chloroform under phase transfer catalytic conditions applying triethylbenzylammonium chloride (TEBAC) was added on the double bond of the phospholene oxide (**1a**–**e**) in the first step that was followed by a silver-nitrate-promoted solvolytic ring opening in water (Scheme 1). While the *P*-alkoxy phosphabicyclo[3.1.0]­hexane 3-oxides **2a**–**d** were obtained as a mixture of two diastereomers (**2A** and **2B**),^30^ the phenyl derivative **2e** was formed as a single isomer (**B**).^31^ Its structure exhibiting the dichlorocyclopropane ring and the oxygen atom of the P=O function in the trans disposition (2**Be**) was confirmed by single crystal X-ray analysis.^33^ In our earlier work, the assignment of the diastereomeric (epimeric) structure to the two species was tentative.^34^ We now have been successful to isolate both isomers of dichlorocarbene adduct **2b** in a crystalline form suitable for single crystal X-ray measurement. Hence, it occurred that the assignment is the other way round, as it was suggested earlier. The isomer displaying a δ_P_ of 86.8 and present in the crude mixture in 51% contains the dichlorocyclopropane ring and the P=O oxygen in cis position (**2Ab**) (Figure 1), while the other species with a δ_P_ of 82.9 and a proportion of 49% is the one exhibiting the cyclopropane ring and the oxygen atom of the P-function in trans relation (**2Bb**) (Figure 2). Now, both diastereomers **2Ab** and **2Bb** could be fully characterized also by ^13^C and ^1^H NMR spectral data.

**Scheme 1:**
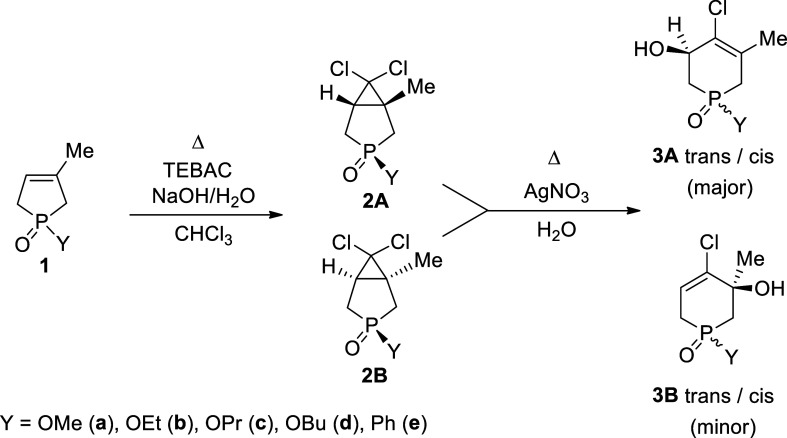
Two-Step Ring Enlargement of 1-Substituted-3-methyl-phospholene 1-Oxides (**1**) to 1,2,3,6-Tetrahydrophosphinine 1-Oxides (**3**)

**Figure 1:**
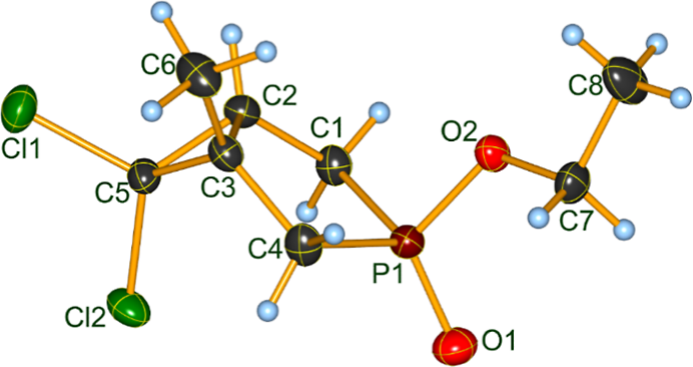
Molecular structure of phosphabicyclo[3.1.0]­hexane 3-oxide **2Ab** in the crystal (see also **2A**, Y = EtO in Scheme 1). DIAMOND^35^ representation; thermal ellipsoids are drawn at 50% probability level.

**Figure 2:**
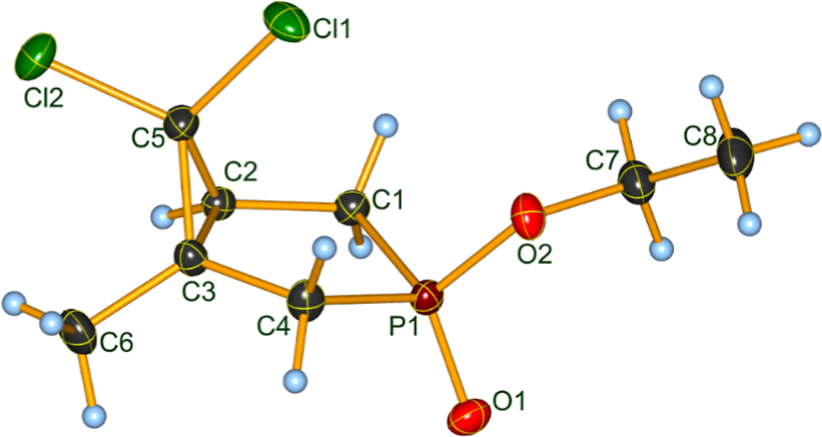
Molecular structure of phosphabicyclo[3.1.0]­hexane 3-oxide **2Bb** in the crystal (see also **2B**, Y = EtO in Scheme 1). DIAMOND^35^ representation; thermal ellipsoids are drawn at 50% probability level.

As regards the second step, to the aqueous (solvolytic) opening of the cyclopropane ring of the 3-phosphabicyclo[3.1.0]­hexane 3-oxides (**2a**–**e**), it may be said that hydroxy-tetrahydrophosphinine oxides (**3a**–**e**) were formed as a mixture of major **3A** and minor **3B** regioisomers both comprising two diastereomers (Scheme 1). The crude mixtures were suitable for further transformations. The known compounds of the above P-heterocyclic pool were identified by ^31^P NMR and HPLC-MS, while the new actors, adduct **2d** and tetrahydrophosphinine oxide **3d** were fully characterized by ^31^P, ^13^C and ^1^H NMR, as well as HRMS. The assignments of the ^13^C NMR signals are based on the typical chemical shifts and J­(P,C) couplings.

### 2.2 X-ray Structure Analysis of Phosphabicyclo[3.1.0]­hexane 3-Oxides **2Ab** and **2Bb**

Phosphabicyclo­[3.1.0]­hexane 3-oxide **2Ab** crystallizes in the centrosymmetric monoclinic *P*2_1_/*n* space group (four molecules in the unit cell), while isomer **2Bb** crystallizes in the also centrosymmetric orthorhombic *Pccn* space group (eight molecules in the unit cell). Details for data collection and structure refinement as well as selected bond lengths and bond angles for both compounds can be found in the Supporting Information (Tables S1–S6). The experimentally determined molecular structures of **2Ab** and **2Bb** show the configuration at the chiral carbon atoms C2 (*S* for **2Ab** and *R* for **2Bb**) and C3 (*R* for **2Ab** and *S* for **2Bb**) regarding the arbitrarily selected enantiomeric molecules of the asymmetric units. Crystallization of both **2Ab** and **2Bb** in centrosymmetric space groups points to the equal probability of the addition of dichlorocarbene from both sides of the double bond of the phospholene oxide ring. Atom distances and bond angles are for both isomers in the expected ranges. The structures show clearly the trans orientation of the cyclopropane ring with respect to the OEt unit at phosphorus in the case of **2Ab** and the respective cis orientation in the case of **2Bb**. In both cases the five-membered phosphole ring is only slightly puckered with a weakly pronounced tendency toward an envelope conformation (Table 1). In fact, the carbon atoms C1–C4 are almost coplanar with the phosphorus atom deviating only slightly from that plane. The offset of the phosphorus from that plane outlined by the four carbon atoms of the phosphole ring is for both compounds away from the cyclopropane ring. The folding is slightly more pronounced for diastereoisomer **2Ab** as compared to form **2Bb**. The interplane angle between the plane of the cyclopropyl ring and the plane formed by the four carbon atoms of the phosphole ring is very similar for both isomers (70.2° for **2Ab** and 70.1° for **2Bb**).

**Table 1:** Torsion Angles (in deg) Observed for the Isomers **2Ab** and **2Bb** ^a^

	**2Ab**	**2Bb**
P1–C1–C2–C3	–10.8(2)	7.5(1)
P1–C4–C3–C2	15.3(2)	–10.9(1)
C1–C2–C3–C4	–3.0(2)	2.3(1)
C5–C3–C2–C1	–111.2(2)	110.6(1)

a Signs of these values for **2Bb** change opposite if the other enantiomer is selected in the cell.

Looking at the epimeric molecular structures, it might seem for the first glance somewhat strange that the two isomers crystallize in different space groups. Analyzing the intermolecular distances in the crystals of the two diastereomers, it becomes evident that the weak interactions between the neighboring molecules in the crystal are indeed different corresponding to the two kinds of packing. For both isomers, two types of weak interactions exist. One of them is a common O···H–C hydrogen bond involving one oxygen atom (O1) of the phosphorus in one molecule, and one of the hydrogen atoms of the methylene group at C4 (**2Ab**) and C1 (**2Bb**) in another molecule. It is interesting to note that C1 and C4 are just on opposite sites in a quasi–enantiomeric relation.

These hydrogen bonds seem to be weak, but through their cooperative effect may be structure determining. The Hirshfeld surface^36^ indicates that the P=O oxygen atom is the sole attractor in diastereomer **2Bb** (see Figure 3).

**Figure 3:**
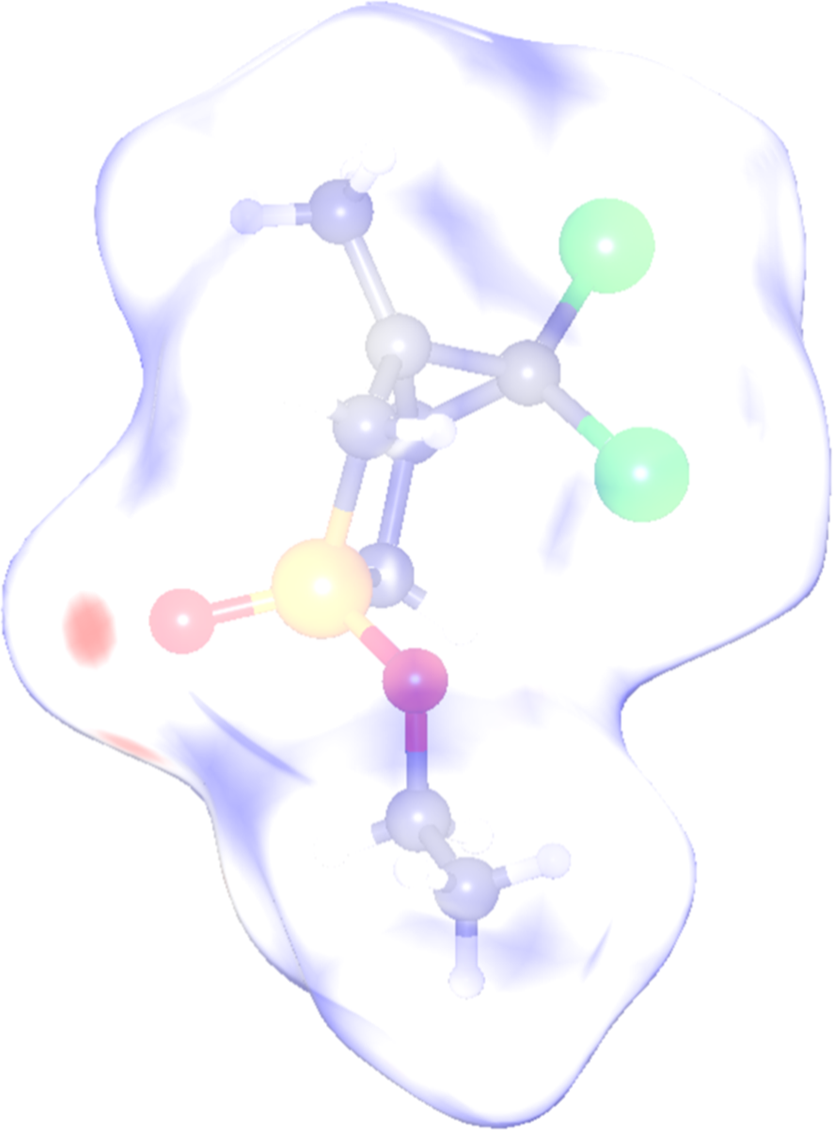
Hirshfeld surface of diastereomer **2Bb** (*d*_norm_ representation, CrystalExplorer V24 ^36^).

The electrostatic potential mapped on the Hirshfeld surface also confirms that the above O atom is the attractor, while the Cl atoms related environment is repulsive (see Figure 4).

**Figure 4:**
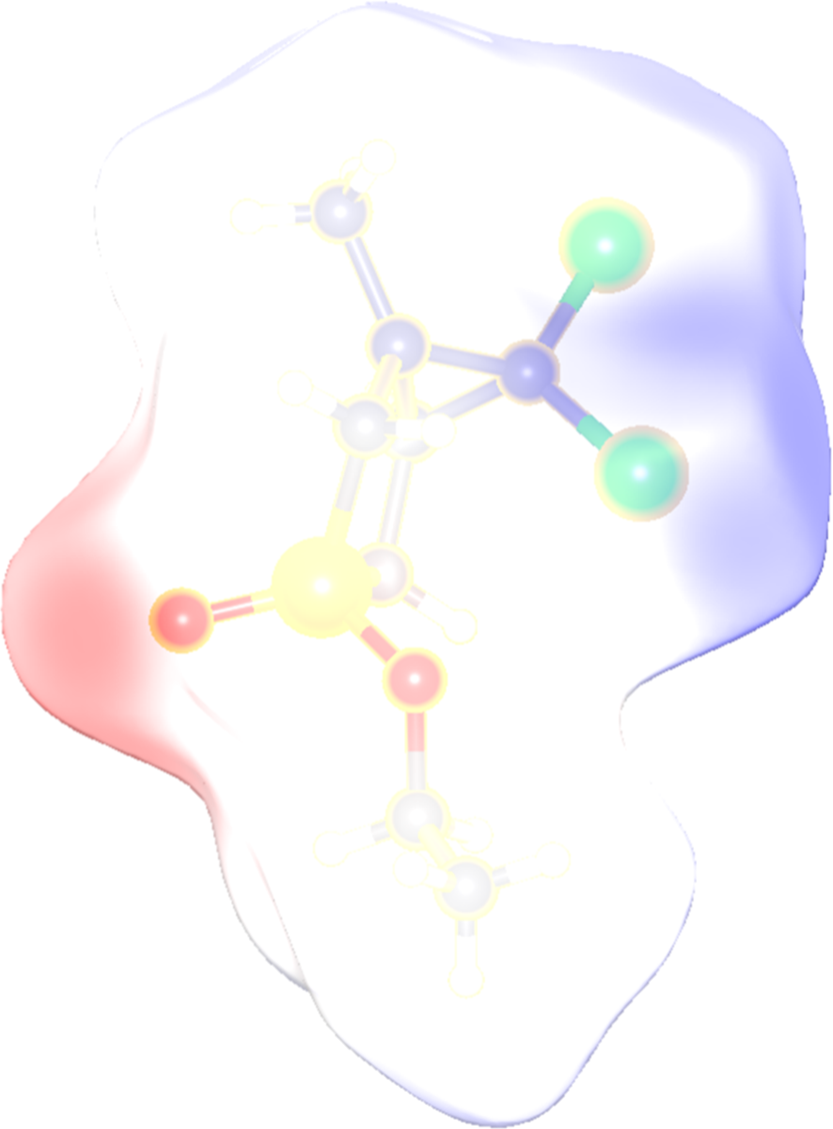
Electrostatic potential colored Hirshfeld Surface of isomer **2Bb**. The EEQ extreme values are −0.1204 and 0.0285 (CrystalExplorer V24 ^36^).

In **2Ab**, the O···H–C contacts form chains running along approximately the *ac* diagonal. These chains are oriented parallel to each other by weak hydrogen bonds involving one chlorine atom of the dichlorocyclopropane ring of one molecule and a proton of the methyl group (C6) at the bridging carbon atom C3 of a molecule of the neighboring chain (Table 2). These contacts altogether span a 2D-layer. Within this layer, the chains are arranged around centers of symmetries such, that two molecules of different chains form dimers based on Cl···H contacts. The layers are oriented roughly parallel to the *c*-axis (Figure 5).

**Table 2:** Values of Short Intermolecular Contacts (H-Bonds) in the Crystals of Isomers **2Ab** and **2Bb** Crystals (Distances in Å, Angles in deg; for Atom Numbering See Figures 5 and 6)­^a^

contact	D–H (Å)	H···A (Å)	D···A (Å)	D–H···A (deg)	
C4–H···O1	0.93(1)	2.44(1)	3.296(2)	153(1)	**2Ab**
C1–H···O1	0.98(1)	2.39(1)	3.350(1)	167(1)	**2Bb**
C6–H···Cl2	0.98(1)	2.97(1)	3.852(2)	149(1)	**2Ab**
C4–H···Cl1	0.95(1)	2.93(1)	3.596(1)	128(2)	**2Bb**

a D and A mean donor and acceptor, respectively. The standard error is grossly underestimated.

**Figure 5:**
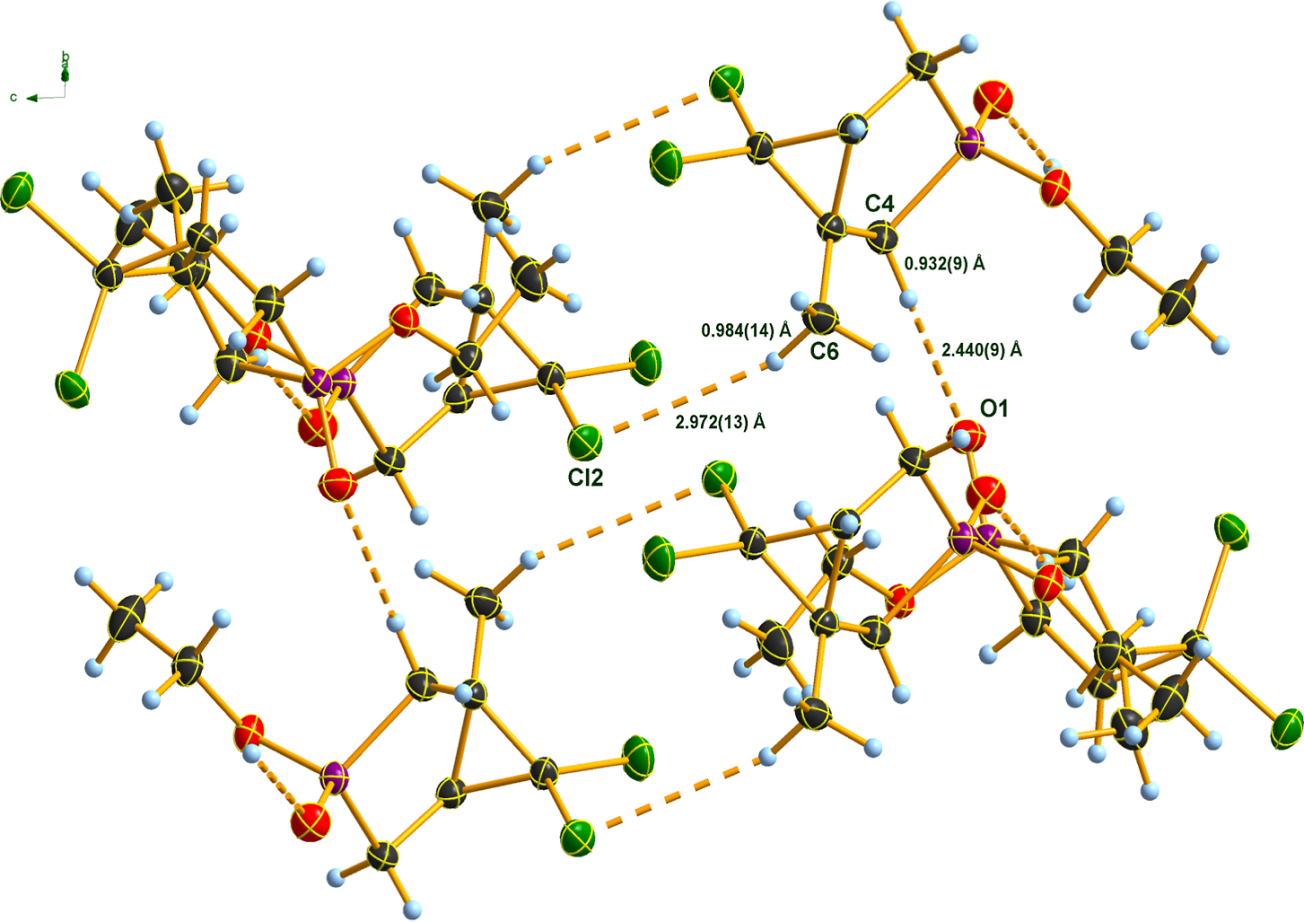
Hydrogen bonding in the crystal of **2Ab**. DIAMOND^35^ representation; thermal ellipsoids are drawn at 50% probability level.

In the case of **2Bb** the arrangement of the molecules in the crystal is also determined by O···H and Cl···H interactions (Table 2). In this case, however, the O···H hydrogen bonds result in the formation of chains with the cyclopropane rings pointing to the same side of the chain. The interaction between the chains is again caused by weak Cl···H bonds, which involve one of the chlorine atoms of one molecule (Cl1) and, in contrast to **2Ab**, one of the protons at the second methylene group (C4) of another molecule, resulting in the formation of double strands interwoven by these H-contacts and parallel to the *c*-axis (Figure 6). Apparently, in the orthorhombic crystal of isomer **2Bb**, mirror planes are the main building symmetry motifs on crystallization, while in species **2Ab**, symmetry centers and a glide plane are decisive. Common to both the monoclinic and the orthorhombic diastereomers is that Cl···Cl contacts exist around the van der Waals radii sums.

**Figure 6:**
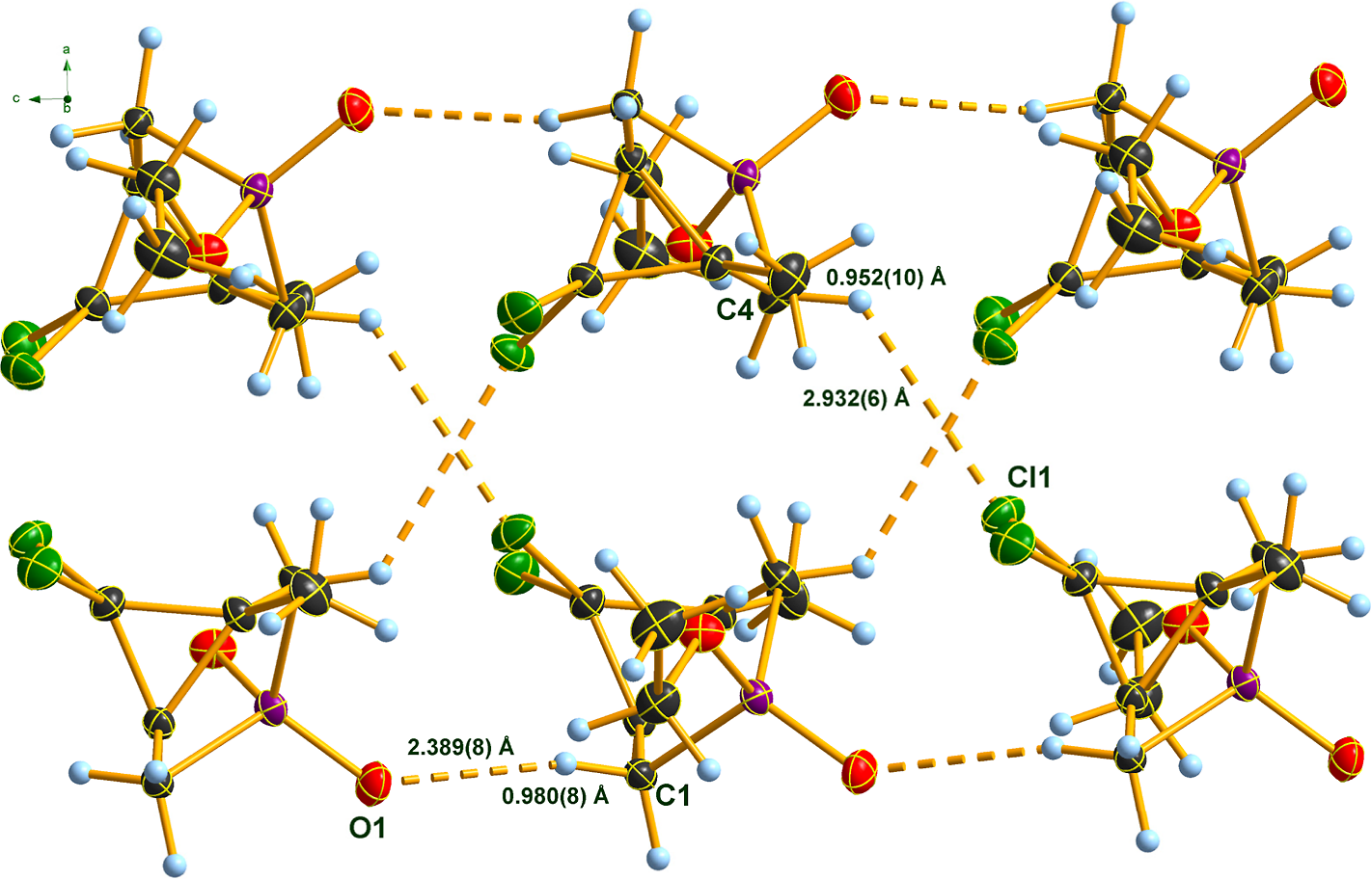
Hydrogen bonding in the crystal of **2Bb**. DIAMOND^35^ representation; thermal ellipsoids are drawn at 50% probability level.

As hydrogen bonding and weak interactions with chlorine atoms may play role in the first contact between APIs and biomolecules, the observed weak interactions in the crystals of **2Ab** and **2Bb** may eventually provide information on tentative attachment/binding loci in further molecular design.

### 2.3 Phosphinoylation and Thiophosphinoylation of 3-Hydroxy-1,2,3,6-tetrahydrophosphinine Oxides

The hydroxy-tetrahydrophosphinine oxides (**3a**–**e**) prepared were then reacted with 1.2 equiv. diphenylphosphinic chloride in the presence of 1.2 equiv of triethylamine in toluene at 25 °C. Starting from the regioisomeric mixture (**A** and **B**) of the tetrahydrophosphinine oxides (**3**), only isomer **3A** took place in the phosphinoylation to afford species **4Aa-e** as a mixture of *trans* and *cis* diastereomers (Scheme 2). Regioisomer **3B** resisted undergoing *O*-phosphinoylation due to sterical hindrance: the hydroxy group is attached to a tertiary carbon atom. The drastically decreased reactivity of tertiary alcohols as compared to primary and secondary alcohols in certain reactions, such as e.g. sulfonylations is well-known.^37^

**Scheme 2:**
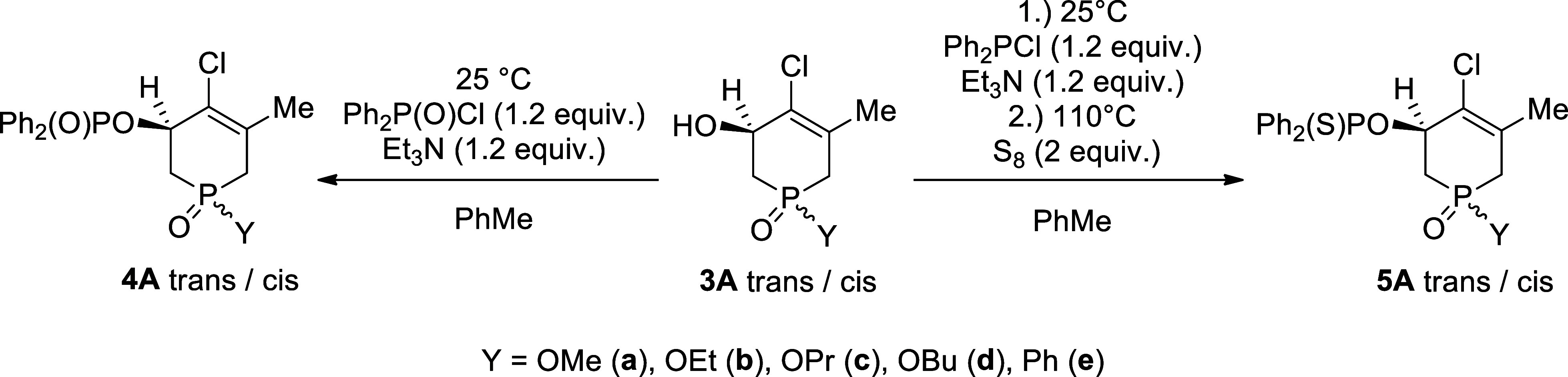
Phosphinoylation and Thiophosphinoylation of 1,2,3,6-Tetrahydrophosphinine Oxides (**3A**)

Moreover, in a few cases, the major diastereomer of product **4A** could be separated from the mixture by chromatography. The analogous thiophosphinoyl derivatives (**5b**, **5c** and **5e**) were synthesized in a one-pot two-step manner. The hydroxy-tetrahydrophosphinine oxides (**3**) were first reacted with 1.2 equiv. of diphenylphosphinous chloride in the presence of 1.2 equiv. triethylamine as above, then 2 equiv of sulfur was added and the temperature was increased to 110 °C to block the introduced trivalent P atom. Eventually, the expected thiophosphinoylated products (**5b**, **5c** and **5e**) were formed, again as diastereoisomeric mixtures (Scheme 2). All doubly functionalized P-ring compounds (**4a**–**e**, **5b**, **5c** and **5e**) were purified by column chromatography. During the chromatography, the major diastereomer was enriched in the mixture, or it was separated. The yields related on the isomeric mixtures fell in the range of 33–60%. If only the useful isomers **A**_**1**_ and **A**_**2**_ are considered, the yields fell in the range of 48–85%. The new compounds were fully characterized by ^31^P, ^13^C and ^1^H NMR data, as well as by HRMS. It is worth mentioning that the phosphinoyl- and thiophosphinoyl-tetrahydrophosphinine oxides exhibited two singlets in the ^31^P NMR spectra.

### 2.4 Cytotoxic Activity of the P-Heterocycles

The results shown in Figure 7 represent the effects of P-heterocycles, such as of 3-phosphabicyclo[3.1.0]­hexane 3-oxides, hydroxy-1,2,3,6-tetrahydrophosphinine oxides and their phosphorylated derivatives on the cell viability of U266 myeloma cells (Figure 7A–D) and A2058 melanoma cells (Figure 7E–H) at two concentrations (10 μM and 100 μM, respectively).

**Figure 7:**
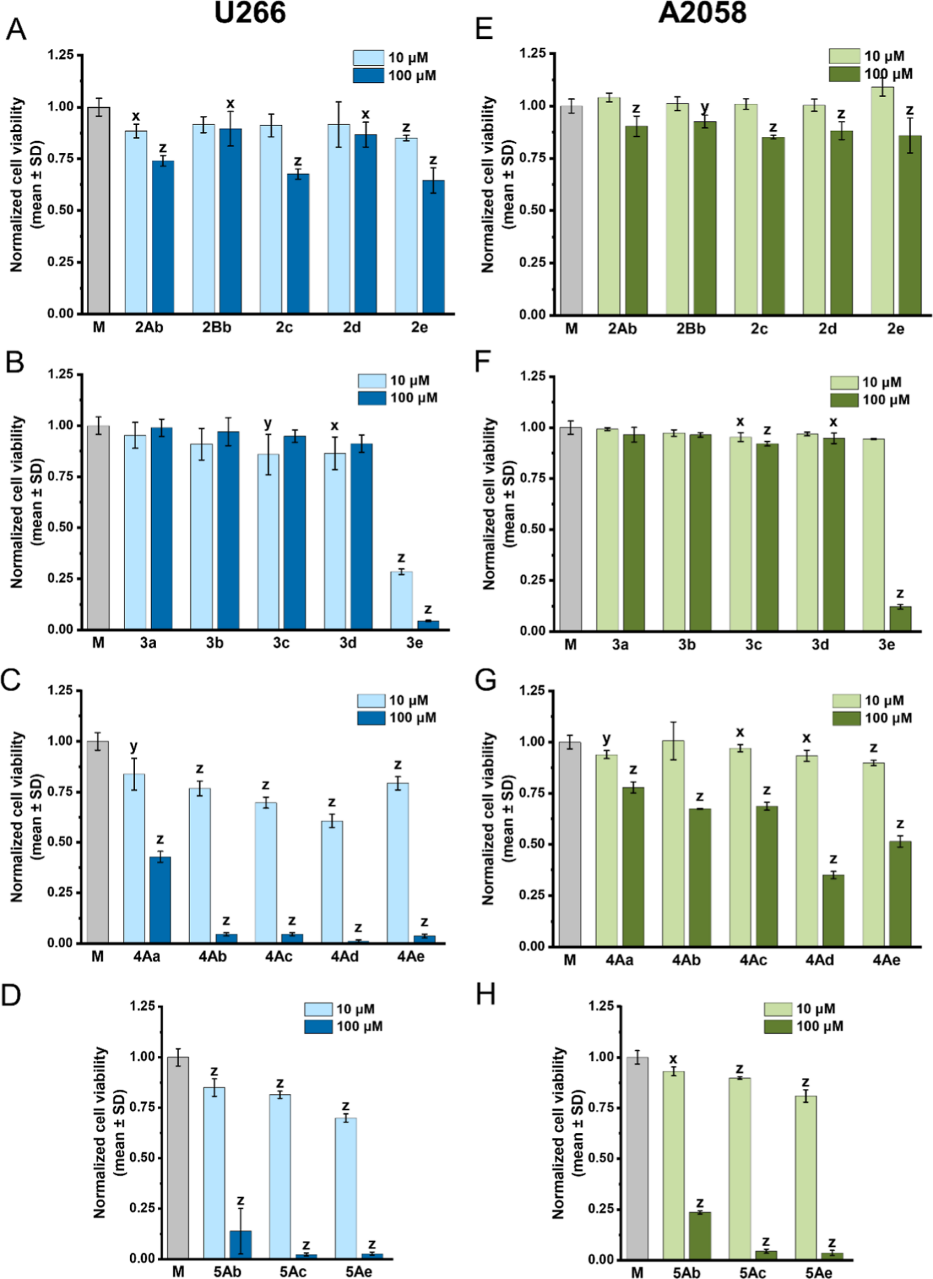
(A–H) In vitro antiproliferative effects of the compounds tested (10 and 100 μM) on U266 (A–D) and on A2058 (E–H) cell line after 72 h. The data are normalized to the control wells. Data represented as the mean ± SD; *n* = 3. The levels of significance are shown as follows: *x*: *p* < 0.05; *y*: *p* < 0.01; *z*: *p* < 0.001, determined by the one-way ANOVA test, followed by Fishers LSD post hoc test.

The members of the 3-phosphabicyclo[3.1.0]­hexane 3-oxide group (**2Ab, 2Bb** and **2c**–**e**) exhibited only mild reductions in cell viability on both cell lines. At 10 μM, viability remained close to control levels for all derivatives, while a treatment with 100 μM caused a moderate decrease, particularly in the case of compound **2c** and **2e**, which induced the strongest reductions within this group (Figure 7A,E). The hydroxy-tetrahydrophosphinine oxide derivatives (**3a–e**) exhibited generally weaker antiproliferative effects as compared with compounds from the previous series. Most members of this group (**3a–d**) showed a similar activity profile, maintaining high cell viability at both tested concentrations in both cell lines. Derivative **3e** represented a notable exception, as it induced a pronounced reduction in cell viability in U266 multiple myeloma cells at both concentrations tested, an effect that was not observed in A2058 melanoma cells at 10 μM. However, at 100 μM, compound **3e** almost completely abolished cell viability in both cell lines (Figure 7B,F). The phosphinoylation of the hydroxy-tetrahydrophosphinine oxides resulted in a set of efficient molecules (**4Aa-e**) regarding their cytotoxic effects, as these molecules had markedly greater impact on the investigated myeloma cell line than the previous P-heterocycles. Already at 10 μM, most compounds reduced the viability, and at 100 μM, nearly all samples resulted in complete loss of the cell viability, except for species **4Aa**, which exhibited only a weaker effect (Figure 7C). In contrast, the A2058 melanoma cells were less sensitive to derivatives **4Aa-4Ae**, with **4Ad** being the only compound capable of reducing cell viability below 50% at 100 μM (Figure 7G). The analogous thiophosphinoyl derivatives (**5Ab**, **5Ac** and **5Ae**) displayed similar effects, as these compounds caused pronounced reductions in the cell viability of the U266 cells, as well as of the A2058 cells, especially at 100 μM (Figure 7D,H).

Based on the IC_50_ values calculated from the dose–response curves (Figure 8.), **3e** and **5Ae** are the most potent compounds, exhibiting the lowest IC_50_ values (15.21 μM and 16.57 μM, respectively). **5Ac** (27.18 μM) and **4Ad** (31.01 μM) show intermediate activity. In contrast, **4Ae**, **4Ab**, **5Ab**, and **4Ac** display lower potency, with IC_50_ values ranging from approximately 40 to 57 μM, with **4Ac** being the least active (56.86 μM). For comparison, in our previous study, bortezomib, a clinically used antimyeloma xenobiotic, exhibited IC_50_ values in the low nanomolar range against A2058 melanoma and U266 cells after 72 h of treatment, demonstrating much higher potency than our compounds, as expected for an established proteasome inhibitor.^38^

**Figure 8:**
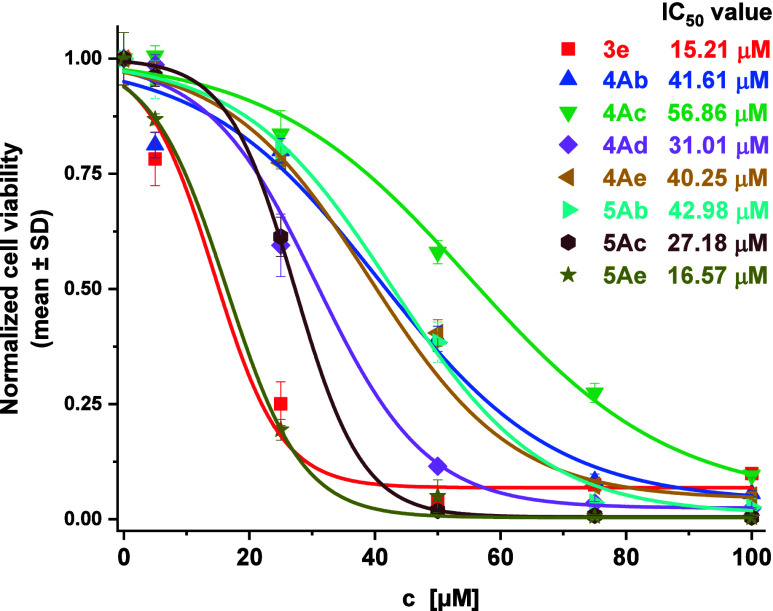
Concentration–response curves for U266 cells treated with compounds **3e**, **4Ab**, **4Ac**, **4Ad**, **4Ae**, **5Ab**, **5Ac** and **5Ae** for 72 h. The data are normalized to the control wells. The IC_50_ value of the derivatives was determined by fitting a sigmoidal dose–response curve to the data, using Origin Pro 2018. The data are given as mean values ± standard deviation (SD), (*n* = 3).

Overall, the cell viability decreased progressively going from 3-phosphabicyclo[3.1.0]­hexane 3-oxide derivatives to phosphinoyl and thiophosphinoyl derivatives, and was consistently lower at 100 μM than at 10 μM on both cell lines. Among the hydroxy-tetrahydrophosphinine oxides (**3**), the phenyl-substituted derivative **3e** showed a distinct activity profile, inducing a marked reduction in viability in U266 multiple myeloma cells at both concentrations tested. In contrast, this effect was not evident in A2058 melanoma cells at 10 μM, that might indicate a cell line–dependent sensitivity. In case of the U266 myeloma cells, similar effects were seen, when the cells were treated with 100 μM phosphinoyl (**4Ab**–**4Ae**) or thiophosphinoyl (**5Ab**, **5Ac** and **5Ae**) derivatives regardless of the substituent on the phosphorus atom in the heterocycle. The A2058 melanoma cells were overall less responsive in terms of antiproliferative effects, as derivatives **4Aa**–**4Ae** induced only moderate reductions in cell viability, even at higher concentrations despite the introduction of substituents expected to increase lipophilicity. Only compound **4Ad** could reduce cell viability lower than 0.5. In contrast, the thiophosphinoylated compounds **5Ab, 5Ac** and **5Ae** displayed a more pronounced antiproliferative activity, particularly at 100 μM, than the phosphinoylated ones. Nevertheless, the overall sensitivity of A2058 cells to the derivatives remained lower than that observed in U266 cells.

According to the literature, the alkoxy- and phenyl substituents differ markedly in their physicochemical properties, which can influence lipophilicity and cellular uptake mechanism, consequently, the impact on the cells. Among the methoxy-, ethoxy-, propoxy- and butoxy substituents, the lipophilicity increases with the length of the alkyl chain, but the highest lipophilicity can be attributed the phenyl group.^39^ The P=O and P=S functionalities may also have an influence on the polarity and lipophilicity. The phosphinoyl group is more polar that may enhance interactions with the target proteins, while the thiophosphinoyl group is more lipophilic that may result in higher intracellular concentrations due to the enhanced cellular uptake through the plasma membrane.^40^ Based on our results, the SAR indicates that increase of the lipophilicity at the phosphorus atom (alkoxy → phenyl) resulted in the most effective derivative (**3e**) on U266 cells. Further modifications, e.g., introduction of a second phosphoryl-type functionality (P=O or P=S) could not contribute to a higher potency on the myeloma cell line. Similar effects were seen on the A2058 melanoma cells, with a notable difference that the thiophosphinoylation appears more advantageous than the phosphinoylation in the less responsive A2058 cells, underscoring the importance of both P=X functions (X = O or S) and cell type in determining biological response. Our findings demonstrate that both the structural modifications across the compound series, and increasing concentrations can contribute significantly to the cytotoxic effects observed. All in all, further investigations are necessary to evaluate the specificity of the studied 1,2,3,6-tetrahydrophosphinine oxide derivatives, and to establish their therapeutic window by comparing their antiproliferative activity in tumorous and non-tumorous cells.

To summarize our results, a new family of P-heterocycles, namely phosphinoylated and thiophosphinoylated 3-hydroxy-1,2,3,6-tetrahydrophosphinine oxides were synthesized in three steps starting from 1-substituted 3-phospholene 1-oxides. The addition of dichlorocarbene to the double bond of the starting materials led to the corresponding 3-phosphabicyclo[3.1.0]­hexane 3-oxides, whose solvolytic ring opening by AgNO_3_/H_2_O afforded 3-hydroxy-tetrahydrophosphinine oxides. Structures of the diastereoisomers of the phospholene oxide–dichlorocarbene adducts were elucidated also by single crystal X-ray analysis. Phosphinoylation and thiophosphinoylation of the hydroxy-tetrahydrophosphinine oxides with Ph_2_P­(O)Cl and Ph_2_PCl + S_8_, respectively, furnished the target compounds comprising two P-functions. The 3-P­(O)­Ph_2_O- and 3-P­(S)­Ph_2_O-substituted tetrahydrophosphinine oxides demonstrated structure-dependent cytotoxic activity, with compounds **3e, 5Ac** and **5Ae** emerging as the most potent derivatives, as reflected by their antiproliferative effects in both tested cell lines and by their low IC_50_ values in U266 multiple myeloma cells. Overall, these compounds exhibited markedly higher antiproliferative effects in U266 cells than in A2058 melanoma cells, indicating a cell line–dependent response. Our results support our hypothesis that chemical modification of the hydroxy-tetrahydrophosphinine oxide scaffold enhances cytotoxic potency.

## 3 Experimental Section

### 3.1 General Information

The ^31^P, ^13^C, and ^1^H NMR spectra were taken on a Bruker DRX-500 or Bruker Avance-300 spectrometer (Bruker, Billerica, MA, USA) operating at 202, 126, and 500 MHz or 122, 75, and 300 MHz, respectively. The couplings are given in Hz. HPLC-MS measurements were performed using a Shimadzu LCMS-2020 device (Shimadzu Corporation, Kyoto, Japan) equipped with a Reprospher 100 C18 (5 μm; 100 × 3 mm) column and positive–negative double ion source (DUIS±) with a quadrupole MS analyzer in a range of 50–1000 *m*/*z*. HRMS measurements were carried out on a Q-TOF Premier mass spectrometer (Waters Corporation, Milford, MA, USA) in positive electrospray ionization mode, using MassLynx 4.1 software.

### 3.2 Preparation of the Starting Materials **2a–e** and **3a–e**

#### 3.2.1 Dichlorocarbene Additions

The dichlorocarbene additions were performed as described earlier.^31,33^ The preparative data and identification were summarized in Table 3.

**Table 3:** Identification of the 3-Phosphabicyclo[3.1.0]­hexane 3-Oxides (**2a–e**)

	diastereomeric composition^a^ (%)					
phosphabicyclo-hexane	**A**	**B**	yield (%)	δ_P_ (CDCl_3_)	δ_P_ (CDCl_3_)^lit.^	HPLC-MS [M + H]^+^
**2a**	95	5	48	88.9 and 86.9	88.1 ^31^	229
**2b**	51	49	52	86.8 and 82.9	86.9 and 82.9 ^33^	243
**2c**	52	48	45	86.8 and 83.0	86.5 and 82.7 ^33^	257
**2d**	52	48	55	86.8 and 83.0	–	271
**2e**	0	100	68	75.9	75.6 ^31^	275

a Determined for the crude products by relative ^31^P NMR intensities.

##### 3.2.1.1 6,6-Dichloro-3-ethoxy-1-methyl-3-phosphabicyclo­[3.1.0]­hexane 3-Oxide (**2b**)

Isomer **2Ab**: ^13^C {^1^H} NMR (75 MHz, CDCl_3_): δ 16.5 (d, *J* = 6.0 Hz, CH_2_ *C*H_3_), 21.5 (d, *J* = 7.6 Hz, C^1^ *C*H_3_), 25.0 (d, *J* = 89.9 Hz, C^4^), 30.8 (d, *J* = 90.1 Hz, C^2^), 31.0 (d, *J* = 13.0 Hz, C^1^), 32.2 (d, *J* = 11.0 Hz, C^5^), 60.6 (d, *J* = 6.4 Hz, OCH_2_), 72.0 (d, *J* = 12.8 Hz, C^6^); ^1^H NMR (300 MHz, CDCl_3_): δ 1.30 (t, *J* = 7.0 Hz, 3H, CH_2_C*H*_3_), 1.50 (s, 3H, C^1^C*H*_3_), 1.63–2.34 (m, 5H, P­(CH_2_)_2_ + CH), 3.97–4.07 (m, 2H, OCH_2_); [M + H]^+^ calcd for C_8_H_14_O_2_Cl_2_P, 243.0103; found, 243.0105; mp: 87–88 °C.

Isomer **2Bb**: ^13^C {^1^H} NMR (75 MHz, CDCl_3_): δ 16.4 (d, *J* = 5.2 Hz, CH_2_ *C*H_3_), 21.8 (d, *J* = 8.0 Hz, C^1^ *C*H_3_), 26.6 (d, *J* = 91.4 Hz, C^4^), 31.8 (d, *J* = 12.8 Hz, C^1^), 32.3 (d, *J* = 93.6 Hz, C^2^), 33.1 (d, *J* = 12.1 Hz, C^5^), 62.1 (d, *J* = 6.8 Hz, OCH_2_), 71.5 (d, *J* = 11.3 Hz, C^6^); ^1^H NMR (300 MHz, CDCl_3_): δ 1.32 (t, *J* = 7.0 Hz, 3H, CH_2_C*H*_3_), 1.61 (s, 3H, C^1^C*H*_3_), 1.81–2.46 (m, 5H, P­(CH_2_)_2_ + CH), 4.04–4.14 (m, 2H, OCH_2_); [M + H]^+^ calcd for C_8_H_14_O_2_Cl_2_P, 243.0103; found, 243.0105; mp: 73–74 °C.

##### 3.2.1.2 6,6-Dichloro-3-butoxy-1-methyl-3-phosphabicyclo­[3.1.0]­hexane 3-Oxide (**2d**)

Isomer **2Ad**: ^13^C {^1^H} NMR (75 MHz, CDCl_3_): δ 13.6 (s, CH_2_ *C*H_3_), 18.65 (s, *C*H_2_CH_3_), 21.5 (d, *J* = 7.6 Hz, C^1^ *C*H_3_), 24.9 (d, *J* = 89.9 Hz, C^4^), 30.7 (d, *J* = 90.5 Hz, C^2^), 31.0 (d, *J* = 13.0 Hz, C^1^), 32.3 (d, *J* = 11.2 Hz, C^5^), 32.5 (d, *J* = 5.9 Hz, OCH_2_ *C*H_2_), 64.4 (d, *J* = 6.7 Hz, OCH_2_), 72.0 (d, *J* = 13.0 Hz, C^6^); ^1^H NMR (300 MHz, CDCl_3_): δ 0.94 (t, *J* = 7.1 Hz, 3H, CH_2_C*H*_3_), 1.36–1.49 (m, “a”, C*H*_2_CH_3_), 1.56 (s, 3H, C^1^C*H*_3_), 1.66–1.88 (m, “b”, OCH_2_C*H*_2_), 1.91–2.50 (m, “c”, P­(CH_2_)_2_ + CH), 3.97–4.07 (m, “d”, OCH_2_).

Isomer **2Bd**: ^13^C {^1^H} NMR (75 MHz, CDCl_3_): δ 13.5 (s, CH_2_ *C*H_3_), 18.74 (s, *C*H_2_CH_3_), 21.7 (d, *J* = 8.2 Hz, C^1^ *C*H_3_), 26.4 (d, *J* = 91.5 Hz, C^4^), 31.8 (d, *J* = 14.4 Hz, C^1^), 32.2 (d, *J* = 91.5 Hz, C^2^), 32.4 (d, *J* = 5.6 Hz, OCH_2_ *C*H_2_), 33.1 (d, *J* = 12.1 Hz, C^5^), 65.7 (d, *J* = 7.1 Hz, OCH_2_), 71.5 (d, *J* = 11.0 Hz, C^6^); ^1^H NMR (300 MHz, CDCl_3_): δ 0.97 (t, *J* = 7.1 Hz, 3H, CH_2_C*H*_3_), 1.36–1.49 (m, “a”, C*H*_2_CH_3_), 1.64 (s, 3H, C^1^C*H*_3_), 1.66–1.88 (m, “b”, OCH_2_C*H*_2_), 1.91–2.50 (m, “c”, P­(CH_2_)_2_ + CH), 3.97–4.07 (m, “d”, OCH_2_); “a”: total int. 4H, “b”: total int. 4H, “c”: total int. 10H, “d”: total int. 4H. [M + Na]^+^ calcd for C_10_H_17_Cl_2_O_2_PNa, 293.0241; found, 293.0241.

#### 3.2.2 Solvolytic Reactions

The solvolytic reactions were carried out as described earlier^30,32^ . The preparative data and identification were summarized in Table 4.

**Table 4:** Identification of the 3-Hydroxy-1,2,3,6-tetrahydrophosphinine 1-Oxide (**3a**–**e**)

	isomeric composition (%)^a^					δ_P_ (CDCl_3_)^b^				δ_P_ (CDCl_3_)^lit^				
compounds	**A**_**1**_	**A**_**2**_	**B**_**1**_	**B**_**2**_	yield (%)	**A**_**1**_	**A**_**2**_	**B**_**1**_	**B**_**2**_	**A**_**1**_	**A**_**2**_	**B**_**1**_	**B**_**2**_	[M + H]^+^
**3a**	37	27	18	18	49	45.2	45.6	47.0	45.0	45.0 ^32^	46.4 ^32^	46.4 ^32^	44.6 ^32^	211
**3b**	37	32	16	15	55	44.5	43.3	44.6	43.0	44.3 ^30^	43.0 ^30^	44.3 ^30^	–	225
**3c**	38	27	21	14	56	44.6	43.6	44.7	43.3	45.5 ^30^	45.5 ^30^	46.3 ^30^	–	239
**3d**	36	30	18	16	68	44.0	42.9	44.2	42.6	–	–	–	–	253
**3e**	45	18	24	13	80	30.4	30.2	30.5	29.1	30.3 ^32^	30.3 ^32^	30.3 ^32^	28.9 ^32^	257

a For the crude mixtures.

b After purification by flash column chromatography.

#### 3.2.3 ^13^C and ^1^H NMR Characterization of 5- and 3-Methyl-4-chloro-1-butoxy-3-hydroxy-1,2,3,6-tetrahydrophosphinine 1-Oxide (**3d**)

See Table 5.

**Table 5:** ^13^C and ^1^H NMR Characterization of 5- and 3-Methyl-4-chloro-1-butoxy-3-hydroxy-1,2,3,6-tetrahydrophosphinine 1-Oxide (**3d**)

^13^C {^1^H} NMR (75 MHz, CDCl_3_) δ				
C	**A_1_**	**A_2_**	**B_1_**	**B_2_**
CH_2_ *C*H_3_	13.5 (bs)^a^			
*C*H_2_CH_3_	18.69 (s)		18.72 (s)	
OCH_2_ *C*H_2_	32.51 (d, *J* = 5.9 Hz)		32.48 (d, *J* = 6.1 Hz)	
OCH_2_	64.7 (d, *J* = 6.7 Hz)	65.1 (d, *J* = 6.4 Hz)	64.8 (d, *J* = 7.0 Hz)	−
C^2^	32.6 (d, *J* = 85.5 Hz)	32.11 (d, *J* = 90.3 Hz)	38.4 (d, *J* = 83.8 Hz)	−
C^3^	69.9 (d, *J* = 3.0 Hz)	70.4 (d, *J* = 4.5 Hz)	73.0 (d, *J* = 2.5 Hz)	−
C^4^	131.1 (d, *J* = 10.2 Hz)	130.6 (d, *J* = 12.1 Hz)	140.6 (d, *J* = 11.5 Hz)	−
C^5^	126.6 (d, *J* = 4.1 Hz)	127.2 (d, *J* = 4.4 Hz)	118.6 (d, *J* = 5.7 Hz)	−
C^6^	32.08 (d, *J* = 89.7 Hz)	32.2 (d, *J* = 85.6 Hz)	26.9 (d, *J* = 89.7 Hz)	−
C*C*H_3_	23.5 (d, *J* = 12.2 Hz)	23.6 (d, *J* = 12.0 Hz)	28.5 (d, *J* = 5.6 Hz)	28.9 (d, *J* = 6.2 Hz)

^1^H NMR (300 MHz, CDCl_3_) δ				
H	**A_1_**	**A_2_**	**B_1_**	**B_2_**
CH_2_C*H*_3_	0.95 (t, *J* = 7.3 Hz, total int. 3H)^a^			
C*H*_2_CH_3_	1.33–1.47 (m, total int. 2H)^a^			
OCH_2_C*H*_2_	1.59–1.74 (m, total int. 2H)^a^			
OCH_2_	4.03−4.16 (m, total int. 2H)^a^			
CCH_3_	1.96 (bs)^b^ ^,^ ^d^		1.55 (bs)^c^ ^,^ ^d^	
P(CH_2_)_2_	2.19–2.66 (m, total int. 4H)^a^ ^,^ ^e^			
OH	2.58 (bs, total int. 1H)^a^ ^,^ ^e^			
CH−O or CH=	4.51–4.68 (m)^b^ ^,^ ^f^		5.81–5.86 and 5.93–5.96 (m)^c^ ^,^ ^f^	

a For all isomers.

b For isomers A_1_/A_2_.

c For isomers B_1_/B_2_.

d Total int. 3H.

e The signals are overlapped.

f Total int. 1H.

### 3.3 General Procedure for the Phosphinoylation of 3-Hydroxy-1,2,3,6-tetrahydrophosphinine Oxides (**3Aa–e**)

To 3-hydroxy-1,2,3,6-tetrahydrophosphinine oxide (1 mmol; **3Aa**: 0.21 g, **3Ab**: 0.23 g, **3Ac**: 0.24 g, **3Ad**: 0.25 g, **3Ae**: 0.26 g), and 1.2 mmol (0.17 mL) of triethylamine in toluene (4.0 mL), 1.2 mmol (0.23 mL) of diphenylphosphinic chloride was added, and the mixture kept at 25 °C for 4 days in a sealed flask under N_2_ atmosphere. The precipitated triethylamine hydrochloride was filtered off, and the solvent was removed in vacuum. The crude product so obtained was purified by column chromatography on silica gel applying DCM-MeOH 95:5 as the eluent to give products **4Aa–e** as yellow oils.

#### 3.3.1 5-Methyl-4-chloro-1-methoxy-3-diphenylphosphinoyl-1,2,3,6-tetrahydrophosphinine 1-Oxide (**4Aa**)

Yield: 0.17 g (41%), Major diastereomer (92%): ^31^P {^1^H} NMR (122 MHz, CDCl_3_): δ_P1_ 33.1 and δ_P2_ 43.2 (s); ^13^C {^1^H} NMR (75 MHz, CDCl_3_): δ 23.8 (d, *J* = 11.8 Hz, C*C*H_3_), 31.6 (d, *J* = 89.5 Hz, C^6^), 32.9 (d, *J* = 85.4 Hz, C^2^), 51.2 (d, *J* = 6.3 Hz, OCH_3_), 72.7 (dd, *J*_1_ = 5.4 Hz, *J*_2_ = 1.4 Hz, C^3^), 127.2 (d, *J*_1_ = 9.5 Hz, *J*_2_ = 7.5 Hz, C^4^), 128.4 and 128.6 (d, *J* = 13.4 Hz, C_β_), 129.7 (d, *J* = 4.7 Hz, C^5^), 130.7 and 131.59 (d, *J* = 136.7 Hz, C_α_), 131.60 and 131.9 (d, *J* = 10.5 Hz, C_γ_), 132.4 and 132.5 (d, *J* = 3.0 Hz, C_δ_); ^1^H NMR (300 MHz, CDCl_3_): δ 1.96 (br s, “a”, CCH_3_), 2.32–2.80 (m, “b”, P­(CH_2_)_2_), 3.69 (d, *J* = 11.1 Hz, “c”, OCH_3_), 5.24–5.31 (m, “d”, CH–O), 7.35–7.58, 7.69–7.80 and 7.84–7.93 (m, “e”, ArH).

Minor diastereomer (8%): ^31^P {^1^H} NMR (122 MHz, CDCl_3_): δ_P1_ 32.9 and δ_P2_ 41.2 (s); ^1^H NMR (300 MHz, CDCl_3_): δ 1.98 (br s, “a”, CCH_3_), 2.32–2.80 (m, “b”, P­(CH_2_)_2_), 3.83 (d, *J* = 11.2 Hz, “c” OCH_3_), 5.24–5.31 (m, “d”, CH–O), 7.35–7.58, 7.69–7.80 and 7.84–7.93 (m, “e”, ArH). “a”: total int. 3H; “b”: total int. 4H; “c”: total int. 3H; “d”: total int. 1H; “e”: total int. 10H. [M + H]^+^ = 411; [M + Na]^+^ calcd for C_19_H_21_ClO_4_P_2_Na, 433.0501; found, 433.0503.

#### 3.3.2 5-Methyl-4-chloro-1-ethoxy-3-diphenylphosphinoyl-1,2,3,6-tetrahydrophosphinine 1-Oxide (**4Ab**)

Yield: 0.14 g (33%); ^31^P {^1^H} NMR (202 MHz, CDCl_3_): δ_P1_ 33.0 and δ_P2_ 41.3 (s); ^13^C {^1^H} NMR (126 MHz, CDCl_3_): δ 16.6 (d, *J* = 5.5 Hz, CH_2_ *C*H_3_), 23.9 (d, *J* = 11.9 Hz, C*C*H_3_), 32.4 (d, *J* = 89.6 Hz, C^6^), 33.5 (d, *J* = 85.4 Hz, C^2^), 61.0 (d, *J* = 6.3 Hz, OCH_2_), 72.8 (d, *J*_1_ = 5.5 Hz, *J*_2_ = 1.5 Hz, C^3^), 127.2 (d, *J*_1_ = 9.2 Hz, *J*_2_ = 7.7 Hz, C^4^), 128.4 and 128.6 (d, *J* = 13.5 Hz, C_β_), 129.8 (d, *J* = 4.7 Hz, C^5^), 130.8 and 131.59 (d, *J* = 136.7 Hz, C_α_), 131.64 and 132.0 (d, *J* = 10.6 Hz, C_γ_), 132.4 and 132.5 (d, *J* = 2.9 Hz, C_δ_); ^1^H NMR (500 MHz, CDCl_3_): δ 1.31 (t, *J* = 7.1 Hz, 3H, CH_2_C*H*_3_), 1.98 (br s, 3H, CCH_3_), 2.35–2.79 (m, 4H, P­(CH_2_)_2_), 4.05–4.10 (m, 2H, OCH_2_), 5.26–5.32 (m, 1H, CH–O), 7.47–7.59 and 7.86–7.91 (m, 10H, ArH); [M + H]^+^ = 425; [M + H]^+^ calcd for C_20_H_24_ClO_4_P_2_, 425.0833; found, 425.0852.

#### 3.3.3 5-Methyl-4-chloro-1-propoxy-3-diphenylphosphinoyl-1,2,3,6-tetrahydrophosphinine 1-Oxide (**4Ac**)

Yield: 0.19 g (43%), Major diastereomer (90%): ^31^P {^1^H} NMR (122 MHz, CDCl_3_): δ_P1_ 32.9 and δ_P2_ 41.2 (s); ^13^C {^1^H} NMR (75 MHz, CDCl_3_): δ 10.0 (s, CH_2_ *C*H_3_), 23.86 (d, *J* = 7.5 Hz, *C*H_2_CH_3_) 23.94 (d, *J* = 10.2 Hz, C*C*H_3_), 32.2 (d, *J* = 89.8 Hz, C^6^), 33.4 (d, *J* = 87.2 Hz, C^2^), 66.5 (d, *J* = 6.5 Hz, OCH_2_), 72.8 (d, *J*_1_ = 6.7 Hz, *J*_2_ = 1.4 Hz, C^3^), 127.2 (d, *J*_1_ = 9.2 Hz, *J*_2_ = 7.6 Hz, C^4^), 128.4 and 128.6 (d, *J* = 13.4 Hz, C_β_), 129.8 (d, *J* = 4.7 Hz, C^5^), 130.8 and 131.6 (d, *J* = 136.6 Hz, C_α_), 131.7 and 132.0 (d, *J* = 10.6 Hz, C_γ_), 132.4 and 132.5 (d, *J* = 3.1 Hz, C_δ_); ^1^H NMR (300 MHz, CDCl_3_) δ 0.90 (t, *J* = 7.4 Hz, “a”, CH_2_C*H*_3_), 1.61–1.80 (m, “b”, C*H*_2_CH_3_), 1.97 (br s, “c”, CCH_3_), 2.32–2.81 (m, “d”, P­(CH_2_)_2_), 3.90–3.98 (m, “e”, OCH_2_), 5.22–5.33 (m, “f”, CH–O), 7.36–7.55, 7.73–7.80 and 7.85–7.91 (m, “g”, ArH).

Minor diastereomer (10%): ^31^P {^1^H} NMR (122 MHz, CDCl_3_): δ_P1_ 32.9 and δ_P2_ 42.6 (s); ^1^H NMR (300 MHz, CDCl_3_): δ 0.99 (t, *J* = 7.2 Hz, “a”, CH_2_C*H*_3_), 1.61–1.80 (m, “b”, C*H*_2_CH_3_), 1.99 (br s, “c”, CCH_3_), 2.32–2.81 (m, “d”, P­(CH_2_)_2_), 4.01–4.11 (m, “e”, OCH_2_), 5.22–5.33 (m, “f”, CH–O), 7.36–7.55, 7.73–7.80 and 7.85–7.91 (m, “g”, ArH).“a”: total int. 3H; “b”: total int. 2H; “c”: total int. 3H; “d”: total int. 4H; “e”: total int.2H; “f”: total int. 1H; “g”: total int. 10H. [M + H]^+^ = 439; [M + Na]^+^ calcd for C_21_H_25_ClO_4_P_2_Na, 461.0814; found, 461.0820.

#### 3.3.4 5-Methyl-4-chloro-1-butoxy-3-diphenylphosphinoyl-1,2,3,6-tetrahydrophosphinine 1-Oxide (**4Ad**)

Yield: 0.17 g (38%), Major diastereomer (93%): ^31^P {^1^H} NMR (122 MHz, CDCl_3_-MeOH 95:5): δ_P1_ 32.9 and δ_P2_ 41.2 (s); ^13^C {^1^H} NMR (75 MHz, CDCl_3_-MeOH 95:5): δ 13.4 (s, CH_2_ *C*H_3_), 18.6 (s, *C*H_2_CH_3_), 23.7 (d, *J* = 11.8 Hz, C*C*H_3_), 32.1 (d, *J* = 89.9 Hz, C^6^), 32.4 (d, *J* = 5.7 Hz, OCH_2_ *C*H_2_), 33.3 (d, *J* = 86.5 Hz, C^2^), 64.6 (d, *J* = 6.4 Hz, OCH_2_), 72.7 (dd, *J*_1_ = 5.5 Hz, *J*_2_ = 1.3 Hz, C^3^), 127.1 (d, *J*_1_ = 9.5 Hz, *J*_2_ = 7.5 Hz, C^4^), 128.3 and 128.5 (d, *J* = 13.4 Hz, C_β_), 129.8 (d, *J* = 4.5 Hz, C^5^), 130.7 and 131.50 (d, *J* = 136.6 Hz, C_α_), 131.52 and 131.9 (d, *J* = 10.5 Hz, C_γ_), 132.34 and 132.27 (d, *J* = 3.0 Hz, C_δ_); ^1^H NMR (300 MHz, CDCl_3_-MeOH 95:5): δ 0.93 (t, *J* = 7.4 Hz, “a”, CH_2_C*H*_3_), 1.25–1.47 (m, “b”, C*H*_2_CH_3_), 1.60–1.72 (m, “c”, OCH_2_C*H*_2_), 1.82 (br s, “d”, CCH_3_), 2.29–2.98 (m, “e”, P­(CH_2_)_2_), 3.94–4.13 (m, “f”, OCH_2_), 5.83–5.97 (m, “g”, CH–O), 7.35–7.56 and 7.73–7.93 (m, “h”, ArH).

Minor diastereomer (7%): ^31^P {^1^H} NMR (122 MHz, CDCl_3_-MeOH 95:5): δ_P1_ 32.0 and δ_P2_ 42.7 (s); ^1^H NMR (300 MHz, CDCl_3_-MeOH 95:5): δ 0.94 (t, *J* = 7.4 Hz, “a”, CH_2_C*H*_3_), 1.25–1.47 (m, “b”, C*H*_2_CH_3_), 1.60–1.72 (m, “c”, OCH_2_C*H*_2_), 2.05 (br s, “d”, CCH_3_), 2.29–2.98 (m, “e”, P­(CH_2_)_2_), 3.94–4.13 (m, “f”, OCH_2_), 6.05–6.13 (m, “g”, CH–O), 7.35–7.56 and 7.73–7.93 (m, “h”, ArH). “a”: total int. 3H; “b”: total int. 2H; “c”: total int. 2H; “d”: total int. 3H; “e”: total int. 4H; “f”: total int. 2H; “g”: total int. 1H; “h”: total int. 10H. [M + H]^+^ = 453; [M + Na]^+^ calcd for C_22_H_27_ClO_4_P_2_Na, 475.0971; found, 475.0971.

#### 3.3.5 5-Methyl-4-chloro-1-phenyl-3-diphenylphosphinoyl-1,2,3,6-tetrahydrophosphinine 1-Oxide (**4Ae**)

Yield: 0.18 g (40%), ^31^P {^1^H} NMR (122 MHz, CDCl_3_): δ_P1_ 26.5 and δ_P2_ 32.5 (s); ^13^C {^1^H} NMR (75 MHz, CDCl_3_): δ 24.0 (d, *J* = 9.0 Hz, C*C*H_3_), 34.3 (d, *J* = 65.3 Hz, C^6^), 35.2 (d, *J* = 64.8 Hz, C^2^), 73.2 (dd, *J*_1_ = *J*_2_ = 5.4 Hz, C^3^), 127.2 (dd, *J*_1_ = 11.4 Hz, *J*_2_ = 7.7 Hz, C^4^), 128.5 (d, *J* = 13.3 Hz, C_β_), 128.5 and 128.7 (d, *J* = 12.8 Hz, C_β′_), 129.8 (d, *J* = 91.4 Hz, C_α_), 129.9 and 130.0 (d, *J* = 133.9 Hz, C_α′_), 130.6 and 131.4 (d, *J* = 10.2 Hz, C_γ′_), 130.9 (d, *J* = 6.2 Hz, C^5^), 132.25 (d, *J* = 10.8 Hz, C_γ_), 132.32 (br s, C_δ_), 132.34 and 132.6 (d, *J* = 2.7 Hz, C_δ′_), ^1^H NMR (300 MHz, CDCl_3_): δ 2.19 (br s, 3H, CCH_3_), 2.71–3.08 (m, 4H, P­(CH_2_)_2_), 5.32–5.45 (m, 1H, CH–O), 7.35–7.91 (m, 15H, ArH); [M + H]^+^ = 457; [M + Na]^+^ calcd for C_24_H_23_ClO_3_P_2_Na, 479.0709; found, 479.0713.

### 3.4 General Procedure for the Thiophosphinoylation of 3-Hydroxy-1,2,3,6-tetrahydrophosphinine Oxides (**3Ab**, **3Ac** and **3Ae**)

To 3-hydroxy-1,2,3,6-tetrahydrophosphinine oxide (1 mmol; **3Ab**: 0.23 g, **3Ac**: 0.24 g, **3Ae**: 0.26 g), and 1.2 mmol (0.17 mL) of triethylamine in toluene (4.0 mL), 1.2 mmol (0.23 mL) of diphenylphosphinous chloride was added, and the mixture kept at 25 °C for 1 h under N_2_ atmosphere in a sealed tube. Then 2 mmol (64 mg) of S_8_ was added to the reaction mixture and stirred for 8 h at 110 °C. The precipitated triethylamine hydrochloride was filtered off, and the solvent was removed in vacuum. The crude product so obtained was purified by column chromatography on silica gel applying DCM-MeOH 95:5 as the eluent to give products **5Ab**, **5Ac** and **5Ae** as yellow oils.

#### 3.4.1 5-Methyl-4-chloro-1-ethoxy-3-diphenylthiophosphinoyl-1,2,3,6-tetrahydrophosphinine 1-Oxide (**5Ab**)

Yield: 0.24 g (55%), Major diastereomer (80%): ^31^P {^1^H} NMR (202 MHz, CDCl_3_): δ_P1_ 41.3 and δ_P2_ 82.7 (s); ^13^C {^1^H} NMR (126 MHz, CDCl_3_): δ 16.6 (d, *J* = 5.5 Hz, CH_2_ *C*H_3_), 23.85 (d, *J* = 12.1 Hz, C*C*H_3_), 32.4 (d, *J* = 90.2 Hz, C^6^), 33.1 (d, *J* = 83.4 Hz, C^2^), 61.1 (d, *J* = 6.4 Hz, OCH_2_), 73.0 (dd, *J*_1_ = 4.9 Hz, *J*_2_ = 1.4 Hz, C^3^), 127.2 (d, *J*_1_ = *J*_2_ = 8.8 Hz, C^4^), 128.22 and 128.5 (d, *J* = 13.8 Hz, C_β_), 129.8 (d, *J* = 4.6 Hz, C^5^), 131.2 and 131.3 (d, *J* = 11.5 Hz, C_γ_), 131.9 and 132.1 (d, *J* = 3.1 Hz, C_δ_), 134.4 and 134.81 (d, *J* = 111.3 Hz, C_α_); ^1^H NMR (500 MHz, CDCl_3_) δ 1.34 (t, *J* = 7.1 Hz, “a”, CH_2_C*H*_3_), 1.95 (br s, “b”, CCH_3_), 2.35–2.84 (m, “c”, P­(CH_2_)_2_), 4.03–4.14 (m, “d”, OCH_2_), 5.57–5.65 (m, “e”, CH–O), 7.41–7.55 and 7.85–7.96 (m, “f”, ArH).

Minor diastereomer (20%): ^31^P {^1^H} NMR (202 MHz, CDCl_3_): δ_P1_ 40.9 and δ_P2_ 82.7 (s); ^13^C {^1^H} NMR (126 MHz, CDCl_3_): δ 16.5 (d, *J* = 6.3 Hz, CH_2_ *C*H_3_), 23.91 (d, *J* = 11.2 Hz, C*C*H_3_), 31.9 (d, *J* = 87.6 Hz, C^2^), 32.8 (d, *J* = 91.5 Hz, C^6^), 61.0 (d, *J* = 6.5 Hz, OCH_2_), 74.0 (dd, *J*_1_ = *J*_2_ = 5.5 Hz, C^3^), 126.5 (d, *J*_1_ = 13.2 Hz, *J*_2_ = 7.2 Hz, C^4^), 128.17 and 128.9 (d, *J* = 13.3 Hz, C_β_), 130.7 and 131.0 (d, *J* = 11.4 Hz, C_γ_), 131.5 (d, *J* = 5.0 Hz, C^5^), 132.0 and 132.6 (d, *J* = 2.9 Hz, C_δ_), 133.5 and 134.82 (d, *J* = 110.2 Hz, C_α_); ^1^H NMR (500 MHz, CDCl_3_): δ 1.23 (t, *J* = 7.0 Hz, “a”, CH_2_C*H*_3_), 1.94 (br s, “b”, CCH_3_), 2.14–2.23 (m, “c”, P­(CH_2_)_2_), 4.03–4.14 (m, “d”, OCH_2_), 5.57–5.65 (m, “e”, CH–O), 7.41–7.55 and 7.85–7.96 (m, “f”, ArH). “a”: total int. 3H; “b”: total int. 3H; “c”: total int. 4H; “d”: total int. 2H; “e”: total int. 1H; “f”: total int. 10H. [M + H]^+^ = 441; [M + Na]^+^ calcd for C_20_H_23_ClO_3_P_2_Na, 463.0429; found, 463.0431.

#### 3.4.2 5-Methyl-4-chloro-1-propoxy-3-diphenylthiophosphinoyl-1,2,3,6-tetrahydrophosphinine 1-Oxide (**5Ac**)

Yield: 0.20 g (45%), Major diastereomer (85%): ^31^P {^1^H} NMR (122 MHz, CDCl_3_): δ_P1_ 41.3 and δ_P2_ 82.7 (s); ^13^C {^1^H} NMR (75 MHz, CDCl_3_): δ 10.07 (s, CH_2_ *C*H_3_), 23.88 (d, *J* = 11.5 Hz, C*C*H_3_), 23.92 (d, *J* = 6.2 Hz, *C*H_2_CH_3_), 32.3 (d, *J* = 90.3 Hz, C^6^), 33.0 (d, *J* = 84.3 Hz, C^2^), 66.5 (d, *J* = 6.6 Hz, OCH_2_), 73.0 (d, *J*_1_ = 4.7 Hz, *J*_2_ = 1.4 Hz, C^3^), 127.3 (d, *J*_1_ = *J*_2_ = 8.7 Hz, C^4^), 128.2 and 128.5 (d, *J* = 13.7 Hz, C_β_), 129.7 (d, *J* = 4.6 Hz, C^5^), 131.2 and 131.3 (d, *J* = 8.7 Hz, C_γ_), 131.9 and 132.1 (d, *J* = 3.1 Hz, C_δ_), 134.48 and 134.8 (d, *J* = 111.3 Hz, C_α_); ^1^H NMR (300 MHz, CDCl_3_): δ 0.96 (t, *J* = 7.4 Hz, “a”, CH_2_C*H*_3_), 1.64–1.73 (m, “b”, C*H*_2_CH_3_), 1.96 (br s, “c”, CCH_3_), 2.38–2.84 (m, “d”, P­(CH_2_)_2_), 3.90–4.05 (m, “e”, OCH_2_), 5.55–5.68 (m, “f”, CH–O), 7.41–7.60 and 7.81–8.05 (m, “g”, ArH).

Minor diastereomer (15%): ^31^P {^1^H} NMR (122 MHz, CDCl_3_): δ_P1_ 41.2 and δ_P2_ 82.7 (s); ^13^C {^1^H} NMR (75 MHz, CDCl_3_): δ 10.13 (s, CH_2_ *C*H_3_), 23.8 (d, *J* = 11.0 Hz, C*C*H_3_), 24.0 (d, *J* = 5.2 Hz, *C*H_2_CH_3_), 31.4 (d, *J* = 86.9 Hz, C^2^), 32.7 (d, *J* = 90.9 Hz, C^6^), 66.4 (d, *J* = 7.4 Hz, OCH_2_), C^3^ and C^4^ are overlapped, 128.6 and 128.7 (d, *J* = 13.1 Hz, C_β_), 130.1 (d, *J* = 5.3 Hz, C^5^), 131.5 (d, *J* = 9.4 Hz, C_γ_), the other C_γ_ is overlapped, 131.7 and 132.2 (d, *J* = 3.1 Hz, C_δ_); ^1^H NMR (300 MHz, CDCl_3_): δ 0.95 (t, *J* = 7.3 Hz, “a”, CH_2_C*H*_3_), 1.64–1.73 (m, “b”, C*H*_2_CH_3_), 1.92 (br s, “c”, CCH_3_), 2.13–2.26 (m, “d”, P­(CH_2_)_2_), 3.90–4.05 (m, “e”, OCH_2_), 5.55–5.68 (m, “f”, CH–O), 7.41–7.60 and 7.81–8.05 (m, “g”, ArH). “a”: total int. 3H; “b”: total int. 2H; “c”: total int. 3H; “d”: total int. 4H; “e”: total int. 2H; “f”: total int. 1H; “g”: total int. 10H. [M + H]^+^ = 455; [M + Na]^+^ calcd for C_21_H_25_ClO_3_P_2_SNa, 477.0586; found, 477.0587.

#### 3.4.3 5-Methyl-4-chloro-1-phenyl-3-diphenylthiophosphinoyl-1,2,3,6-tetrahydrophosphinine 1-Oxide (**5Ae**)

Yield: 0.28 g (60%), Major diastereomer (70%): ^31^P {^1^H} NMR (122 MHz, CDCl_3_): δ_P1_ 25.9 and δ_P2_ 83.3 (s); ^13^C {^1^H} NMR (75 MHz, CDCl_3_): δ 23.6 (d, *J* = 9.6 Hz, C*C*H_3_), 34.4 (d, *J* = 65.6 Hz, C^6^), 34.6 (d, *J* = 63.9 Hz, C^2^), 72.9 (dd, *J*_1_ = *J*_2_ = 4.8 Hz, C^3^), C^4^ is overlapped, 128.2 and 128.6 (d, *J* = 13.9 Hz, C_β’_), 129.1 (d, *J* = 12.1 Hz, C_β_), 129.7 (d, *J* = 9.6 Hz, C_γ_), 130.5 (d, *J* = 5.8 Hz, C^5^), 131.3 and 131.4 (d, *J* = 11.8 Hz, C_γ’_), 131.69 (br s, C_δ_), 132.0 and 132.89 (d, *J* = 3.1 Hz, C_δ’_), 134.2 and 134.8 (d, *J* = 113.6 Hz, C_α’_), 134.6 (d, *J* = 89.8 Hz, C_α_); ^1^H NMR (300 MHz, CDCl_3_): δ 1.96 (br s, bs, “a”, CCH_3_), 2.57–3.12 (m, “b”, P­(CH_2_)_2_), 5.51–5.69 (m, “c”, CH–O), 7.36–8.09 (m, “d”, ArH).

Minor diastereomer (30%): ^31^P {^1^H} NMR (122 MHz, CDCl_3_): δ_P1_ 26.0 and δ_P2_ 83.3 (s); ^13^C {^1^H} NMR (75 MHz, CDCl_3_): δ 24.1 (d, *J* = 9.1 Hz, C*C*H_3_), 34.7 (d, *J* = 65.2 Hz, C^2^), 34.9 (d, *J* = 66.2 Hz, C^6^), C^3^ and C^4^ are overlapped, 128.7 and 128.8 (d, *J* = 13.6 Hz, C_β’_), C_β_ is overlapped, 130.1 (d, *J* = 9.6 Hz, C_γ_), C^5^ is overlapped, 131.67 and 131.90 (d, *J* = 10.8 Hz, C_γ’_), 132.2 and 132.38 (d, *J* = 2.8 Hz, C_δ’_), 132.40 (br s, C_δ_), C_α_ and C_α’_ are overlapped; ^1^H NMR (300 MHz, CDCl_3_): δ 1.94 (br s, bs, “a”, CCH_3_), 2.57–3.12 (m, “b”, P­(CH_2_)_2_), 5.51–5.69 (m, “c”, CH–O), 7.36–8.09 (m, “d”, ArH). “a”: total int. 3H; “b”: total int. 4H; “c”: total int. 1H; “d”: total int. 15H. [M + H]^+^ = 473; [M + Na]^+^ calcd for C_24_H_23_ClO_2_P_2_SNa, 495.0480; found, 495.0483.

### 3.5 Single Crystal X-ray Experimental

Single crystals of compound **2Ab** and **2Bb**, suitable for X-ray diffraction, were obtained by slow evaporation of acetone solution. The crystals were introduced into perfluorinated oil (FOMBLIN Y LVAC grade 25/6 Perfluorinated Polyether, SPI Supplier) and a suitable single crystal was carefully mounted on the top of a thin glass wire. Data collection was performed with an Oxford Xcalibur 3 diffractometer equipped with a Spellman generator (50 kV, 40 mA) and a Kappa CCD detector, operating with Mo–K_α_ radiation (λ = 0.71071 Å).

Data collection and data reduction were performed with the CrysAlisPro software.^41^ Absorption correction using the multiscan method^41^ was applied. The structures were solved with SHELXS-97,^42^ refined with SHELXL-97 ^43^ and finally checked using PLATON.^44^ Details for data collection and structure refinement are summarized in Table 6.

CCDC-2492690–2492691 contains supplementary crystallographic data for this compound. These data can be obtained free of charge from The Cambridge Crystallographic Data Centre via www.ccdc.cam.ac.uk/data_request/cif.

### 3.6 Bioactivity Experimental

#### 3.6.1 Cell Culturing

The U266 multiple myeloma (85051003, ECACC, Salisbury, UK) and the A2058 metastatic melanoma cell line (91100402, ECACC, Salisbury, UK) were selected to perform our cytotoxicity experiments. The U266 cell line grows in suspension, while the A2058 is an adherent cell line. They were both cultured RPMI 1640 (Sigma Ltd., St. Louis, MO, USA) supplemented 10% fetal bovine serum (Invitrogen Corporation, New York, NY, USA), 1% l-glutamine (Invitrogen Corporation, New York, NY, USA), and 1% penicillin/streptomycin (Invitrogen Corporation, New York, NY, USA).

#### 3.6.2 Cell Viability Assay

The CellTiter-Glo Luminescent Cell Viability Assay (Promega, Madison, WI, USA) was performed following the protocol described in our previous publication.^18^ All experiments were conducted in triplicate within a single experimental run, and results were normalized to the DMSO vehicle control using OriginPro 8 software (OriginLab Corporation, Northampton, MA, USA) and reported as mean ± standard deviation (SD).

**Table 6:** Details for X-ray Data Collection and Structure Refinement for Compound **2Ab** and **2Bb**

	**2Ab**	**2Bb**
empirical formula	C_8_H_13_C_l2_O_2_P	C_8_H_13_C_l2_O_2_P
formula mass	243.05	243.05
*T* [K]	123(2)	123(2)
crystal size [mm]	0.20 × 0.20 × 0.02	0.35 × 0.15 × 0.05
crystal description	colorless platelet	colorless block
crystal system	monoclinic	orthorhombic
space group	*P*2_1_/*n*	*Pccn*
*a* [Å]	8.1971(3)	17.4791(4)
*b* [Å]	7.0439(3)	11.0173(2)
*c* [Å]	18.8656(7)	11.0732(2)
α [deg]	90.0	90.0
β [deg]	94.934(4)	90.0
γ [deg]	90.0	90.0
*V* [Å^3^]	1085.25(7)	2132.39(7)
*Z*	4	8
ρ_calcd._ [g cm^–3^]	1.488	1.514
μ [mm^–1^]	0.712	0.724
*F*(000)	504	1008
Θ range [deg]	2.63–25.24	2.33–25.24
index ranges	–11 ≤ *h* ≤ 11	–24 ≤ *h* ≤ 24
	–10 ≤ *k* ≤ 10	–15 ≤ *k* ≤ 15
	–26 ≤ *l* ≤ 26	–15 ≤ *l* ≤ 15
Reflns. collected	21,426	41,788
Reflns. obsd	2743	2892
Reflns. unique	3315 (*R*_int_ = 0.0577)	3258 (*R*_int_ = 0.0301)
*R*_1_, w*R*_2_ (2σ data)	0.0363, 0.0885	0.0252, 0.0647
*R*_1_, w*R*_2_ (all data)	0.0475, 0.0942	0.0303, 0.0676
GOOF on *F* ^2^	1.068	1.116
peak/hole [e Å^–3^]	0.537/–0.358	0.404/–0.227

## Supplementary Material



## Notes

Geometrical data for phosphabicyclo[3.1.0]­hexane 3-oxide **2Ab** and **2Bb** obtained from the X-ray measurements; Details for data collection and structure refinement as well as selected bond lengths and bond angles for both compounds can be found in the Tables S1–S6; ^31^P, ^13^C, ^1^H NMR spectra for the compounds **2a**, **2Ab**, **2bB**, **2d**–**e**, **3a**–**e**, **4Aa-e**, **5Ab**, **5Ac** and **5Ae** (DOCX)
