# Supplementary material for: Cytotoxic Agents by the Phosphinoylation and Thiophosphinoylation of 3‑Hydroxy-1,2,3,6-tetrahydrophosphinine 1‑Oxides as β‑Hydroxyphosphonates and -Phosphine Oxides
Source: ACS Omega. 2026 Feb 26;11(9):14964–75. doi: 10.1021/acsomega.5c11546 (PMC12980232; doi:10.1021/acsomega.5c11546)

**Supporting Information**

**Cytotoxic Agents by the Phosphinoylation and Thiophosphinoylation of 3-Hydroxy-1,2,3,6-tetrahydrophosphinine 1-Oxides as β-Hydroxyphosphonates and -phosphine Oxides**

**Zsuzsanna Szalai,^1^ Kristóf Szloboda,^1^ Konstantin Karaghiosoff,^2^ Mátyás Czugler,^1^ Angéla Takács,^3^ László Kőhidai,^3^ Ágnes Gömöry,^4^ László Drahos,^4^ György Keglevich^1,*^**

^1^Department of Organic Chemistry and Technology, Faculty of Chemical Technology and Biotechnology, Budapest University of Technology and Economics, Műegyetem rkp. 3, 1111 Budapest, Hungary; [szalai.zsuzsanna@edu.bme.hu](mailto:szalai.zsuzsanna@edu.bme.hu) (Z.S.); [kristof.szloboda@edu.bme.hu](file:///C:\Users\Zsuzsi\Desktop\8.félév\Publikációk\ACS%20Omega\SI\kristof.szloboda@edu.bme.hu) (K.S.); [amatyasczx@protonmail.com](mailto:amatyasczx@protonmail.com) (C.M.); [keglevich.gyorgy@vbk.bme.hu](file:///C:\Users\Zsuzsi\Desktop\8.félév\Publikációk\ACS%20Omega\SI\keglevich.gyorgy@vbk.bme.hu)

^2^Department Chemie, Ludwig-Maximilians-Universität München, Butenandtstr. 5-13., D-81377 München, Germany; [klk@cup.uni-muenchen.de](file:///C:\Users\Zsuzsi\Desktop\8.félév\Publikációk\ACS%20Omega\SI\klk@cup.uni-muenchen.de)

^3^Department of Genetics, Cell and Immunobiology, Semmelweis University, Nagyvárad tér 4, 1089 Budapest, Hungary; [takacs.angela@semmelweis.hu](file:///C:\Users\Keglevich%20György\AppData\Local\Microsoft\Windows\INetCache\Content.Outlook\B539O69B\takacs.angela@semmelweis.hu) (A.T.); [kohidai.laszlo@semmelweis.hu](file:///C:\Users\Keglevich%20György\AppData\Local\Microsoft\Windows\INetCache\Content.Outlook\B539O69B\kohidai.laszlo@semmelweis.hu) (L.K.)

^4^MS Proteomics Research Group, Research Centre for Natural Sciences, 1117 Budapest, Hungary; [drahos.laszlo@ttk.hu](mailto:drahos.laszlo@ttk.hu) (L.D.); [gomory.agnes@ttk.hu](mailto:gomory.agnes@ttk.hu) (A.G.)

**Table of contents**

[1. Geometrical data for phosphabicyclo[3.1.0]hexane 3-oxides **2Ab** and **2Bb** obtained from the X-ray measurements S2](#_Toc211504533)

[2. ^31^P, ^13^C, ^1^H NMR spectra for the compounds **2a**, **2Ab**, **2bB**, **2d-e**, **3a-e**, **4Aa-e**, **5Ab**, **5Ac** and **5Ae** synthesized S8](#_Toc211504534)

# Geometrical data for phosphabicyclo[3.1.0]hexane 3-oxide 2Ab and 2Bb obtained from the X-ray measurements

**Table S1.** Selected bond lengths (Å) of phosphabicyclo[3.1.0]hexane 3-oxide **2Ab**.

| Cl2 – C5 | 1.758(2) | C3 – C2 | 1.552(2) |
| --- | --- | --- | --- |
| P1 – O1 | 1.475(1) | C7 – C8 | 1.497(3) |
| P1 – O2 | 1.590(1) | C5 – C2 | 1.498(2) |
| P1 – C1 | 1.809(2) | C5 – C3 | 1.504(2) |
| P1 – C4 | 1.813(2) | C1 – C2 | 1.514(2) |
| Cl1 – C5 | 1.757(2) | C3 – C6 | 1.504(2) |
| O2 – C7 | 1.461(2) | C3 – C4 | 1.523(2) |

**Table S2.** Selected bond angles (°) of phosphabicyclo[3.1.0]hexane 3-oxide **2Ab**.

| O1 – P1 – O2 | 114.5(1) | O2 – C7 – C8 | 107.8(1) |
| --- | --- | --- | --- |
| O1 – P1 – C1 | 118.5(1) | C3 – C4 – P1 | 106.0(1) |
| O2 – P1 – C1 | 100.1(1) | C2 – C1 – P1 | 105.5(1) |
| O1 – P1 – C4 | 117.0(1) | C6 – C3 – C5 | 119.9(1) |
| O2 – P1 – C4 | 105.9(1) | C6 – C3 – C4 | 117.0(1) |
| C1 – P1 – C4 | 98.3(1) | C5 – C3 – C4 | 116.2(1) |
| C7 – O2 – P1 | 118.9(1) | C6 – C3 – C2 | 120.2(1) |
| C2 – C5 – C3 | 62.3(1) | C5 – C3 – C2 | 58.7(1) |
| C2 – C5 – Cl1 | 117.7(1) | C4 – C3 – C2 | 111.9(1) |
| C3 – C5 – Cl1 | 118.9(1) | C5 – C2 – C1 | 119.6(1) |
| C2 – C5 – Cl2 | 119.5(1) | C5 – C2 – C3 | 59.1(1) |
| C3 – C5 – Cl2 | 120.6(1) | C1 – C2 – C3 | 114.2(1) |
| Cl1 – C5 – Cl2 | 110.6(1) |  |  |

**Table S3.** Selected torsion angles (°) of phosphabicyclo[3.1.0]hexane 3-oxide **2Ab**.

| O1 – P1 – O2 – C7 | -50.8(1) | Cl1 – C5 – C2 – C3 | 109.9(1) |
| --- | --- | --- | --- |
| C1 – P1 – O2 – C7 | -178.7(1) | Cl2 – C5 – C2 – C3 | -111.3(1) |
| C4 – P1 – O2 – C7 | 79.6(1) | P1 – C1 – C2 – C5 | -77.6(2) |
| O1 – P1 – C1 – C2 | 144.5(1) | P1 – C1 – C2 – C3 | -10.8(2) |
| O2 – P1 – C1 – C2 | -90.3(1) | C6 – C3 – C2 – C5 | -108.8(2) |
| C4 – P1 – C1 – C2 | 17.6(1) | C4 – C3 – C2 – C5 | 108.2(2) |
| C2 – C5 – C3 – C6 | 109.2(2) | C6 – C3 – C2 – C1 | 140.0(2) |
| Cl1 – C5 – C3 – C6 | 1.2(2) | C5 – C3 – C2 – C1 | -111.2(2) |
| Cl2 – C5 – C3 – C6 | -141.3(1) | C4 – C3 – C2 – C1 | -3.0(2) |
| C2 – C5 – C3 – C4 | -100.8(2) | P1 – O2 – C7 – C8 | -167.7(1) |
| Cl1 – C5 – C3 – C4 | 151.2(1) | C6 – C3 – C4 – P1 | -129.1(1) |
| Cl2 – C5 – C3 – C4 | 8.8(2) | C5 – C3 – C4 – P1 | 80.0(2) |
| Cl1 – C5 – C3 – C2 | -108.0(1) | C2 – C3 – C4 – P1 | 15.2(2) |
| Cl2 – C5 – C3 – C2 | 109.6(1) | O1 – P1 – C4 – C3 | -147.3(1) |
| C3 – C5 – C2 – C1 | 102.0(2) | O2 – P1 – C4 – C3 | 83.7(1) |
| Cl1 – C5 – C2 – C1 | -148.1(1) | C1 – P1 – C4 – C3 | -19.4(1) |
| Cl2 – C5 – C2 – C1 | -9.3(2) |  |  |

**Table S4.** Selected bond lengths (Å) of phosphabicyclo[3.1.0]hexane 3-oxide **2Bb**.

| Cl2 – C5 | 1.761(1) | C5 – C3 | 1.508(2) |
| --- | --- | --- | --- |
| P1 – O1 | 1.480(1) | C2 – C3 | 1.550(1) |
| P1 – O2 | 1.587(1) | C7 – C8 | 1.503(2) |
| P1 – C4 | 1.816(1) | C4 – C3 | 1.526(2) |
| P1 – C1 | 1.820(1) | C6 – C3 | 1.508(2) |
| Cl1 – C5 | 1.757(1) | C5 – C2 | 1.497(1) |
| C1 – C2 | 1.519(1) | O2 – C7 | 1.455(1) |

**Table S5.** Selected bond angles (°) of phosphabicyclo[3.1.0]hexane 3-oxide **2Bb**.

| O1 – P1 – O2 | 115.0(1) | O2 – C7 – C8 | 108.1(1) |
| --- | --- | --- | --- |
| O1 – P1 – C4 | 115.8(1) | C3 – C4 – P1 | 106.5(1) |
| O2 – P1 – C4 | 103.2(1) | C5 – C3 – C6 | 119.7(1) |
| O1 – P1 – C1 | 113.0(1) | C5 – C3 – C4 | 116.4(1) |
| O2 – P1 – C1 | 109.3(1) | C6 – C3 – C4 | 116.7(1) |
| C4 – P1 – C1 | 99.0(1) | C5 – C3 – C2 | 58.6(1) |
| C2 – C1 – P1 | 105.4(1) | C6 – C3 – C2 | 120.4(1) |
| C7 – O2 – P1 | 119.6(1) | C4 – C3 – C2 | 112.2(1) |
| C2 – C5 – C3 | 62.1(1) | Cl1 – C5 – Cl2 | 110.7(1) |
| C2 – C5 – Cl1 | 119.7(1) | C5 – C2 – C1 | 119.3(1) |
| C3 – C5 – Cl1 | 120.2(1) | C5 – C2 – C3 | 59.3(1) |
| C2 – C5 – Cl2 | 117.9(1) | C1 – C2 – C3 | 114.8(1) |
| C3 – C5 – Cl2 | 118.7(1) |  |  |

**Table S6.** Selected torsion angles (°) of phosphabicyclo[3.1.0]hexane 3-oxide **2Bb**.

| O1 – P1 – C1 – C2 | 110.7(1) | C2 – C5 – C3 – C6 | -109.5(1) |
| --- | --- | --- | --- |
| O2 – P1 – C1 – C2 | -119.8(1) | Cl1 – C5 – C3 – C6 | 140.7(1) |
| C4 – P1 – C1 – C2 | -12.3(1) | Cl2 – C5 – C3 – C6 | -1.2(1) |
| O1 – P1 – O2 – C7 | 59.1(1) | C2 – C5 – C3 – C4 | 101.0(1) |
| C4 – P1 – O2 – C7 | -173.8(1) | Cl1 – C5 – C3 – C4 | -8.8(1) |
| C1 – P1 – O2 – C7 | -69.3(1) | Cl2 – C5 – C3 – C4 | -150.7(1) |
| C3 – C5 – C2 – C1 | -103.2(1) | Cl1 – C5 – C3 – C2 | -109.8(1) |
| Cl1 – C5 – C2 – C1 | 7.6(1) | Cl2 – C5 – C3 – C2 | 108.3(1) |
| Cl2 – C5 – C2 – C1 | 147.3(1) | P1 – C4 – C3 – C5 | -75.7(1) |
| Cl1 – C5 – C2 – C3 | 110.7(1) | P1 – C4 – C3 – C6 | 133.8(1) |
| Cl2 – C5 – C2 – C3 | -109.5(1) | P1 – C4 – C3 – C2 | -10.9(1) |
| P1 – C1 – C2 – C5 | 74.8(1) | C1 – C2 – C3 – C5 | 110.6(1) |
| P1 – C1 – C2 – C3 | 7.5(1) | C5 – C2 – C3 – C6 | 108.4(1) |
| P1 – O2 – C7 – C8 | -157.0(1) | C1 – C2 – C3 – C6 | -141.0(1) |
| O1 – P1 – C4 – C3 | -107.3(1) | C5 – C2 – C3 – C4 | -108.3(1) |
| O2 – P1 – C4 – C3 | 126.2(1) | C1 – C2 – C3 – C4 | 2.3(1) |
| C1 – P1 – C4 – C3 | 13.8(1) |  |  |


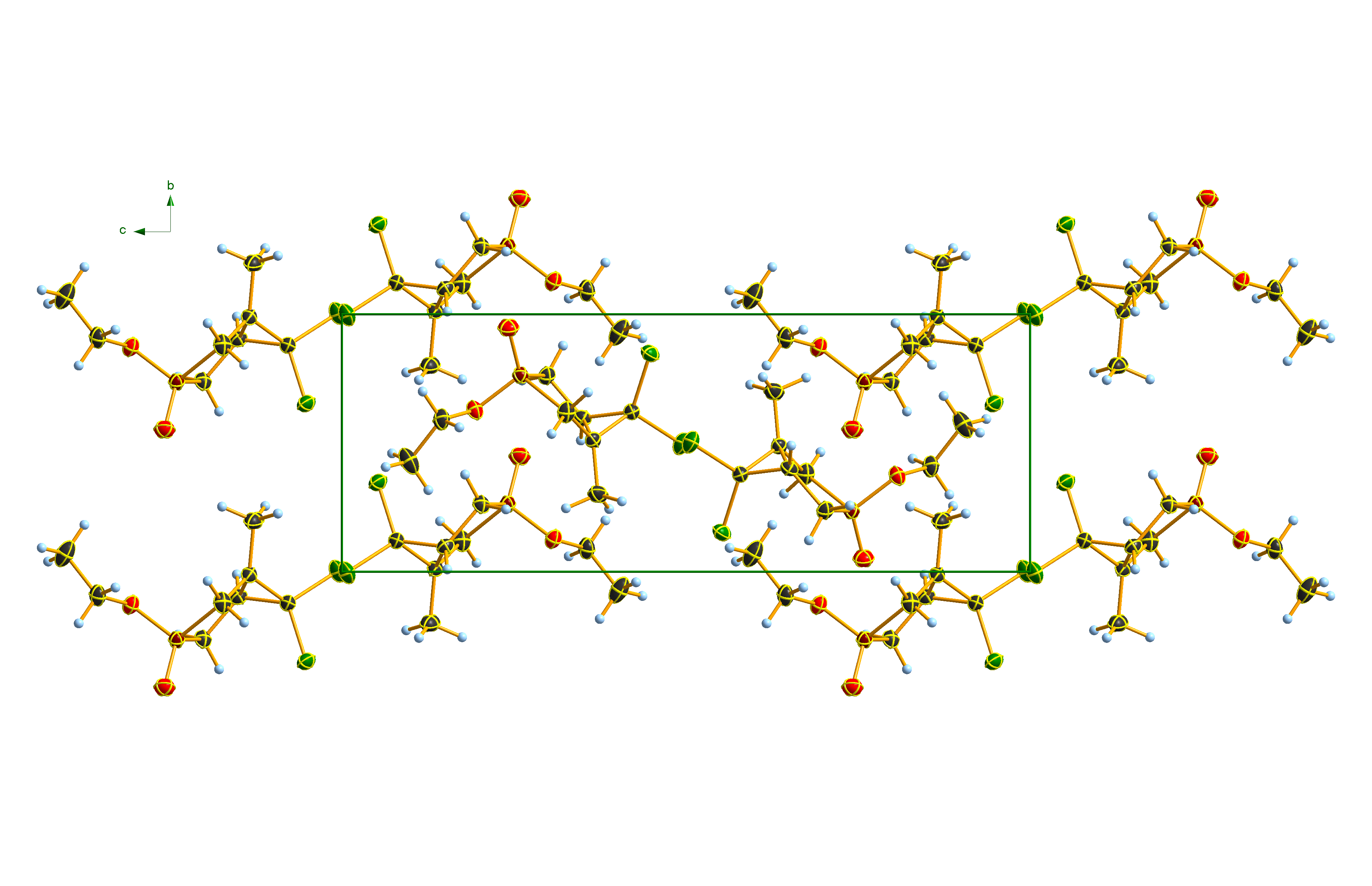


**Figure S1.** Crystal structure of phosphabicyclo[3.1.0]hexane 3-oxide **2Ab**, view of the unit cell along the *a*-axis. DIAMOND representation; thermal ellipsoids are drawn at 50 % probability level.


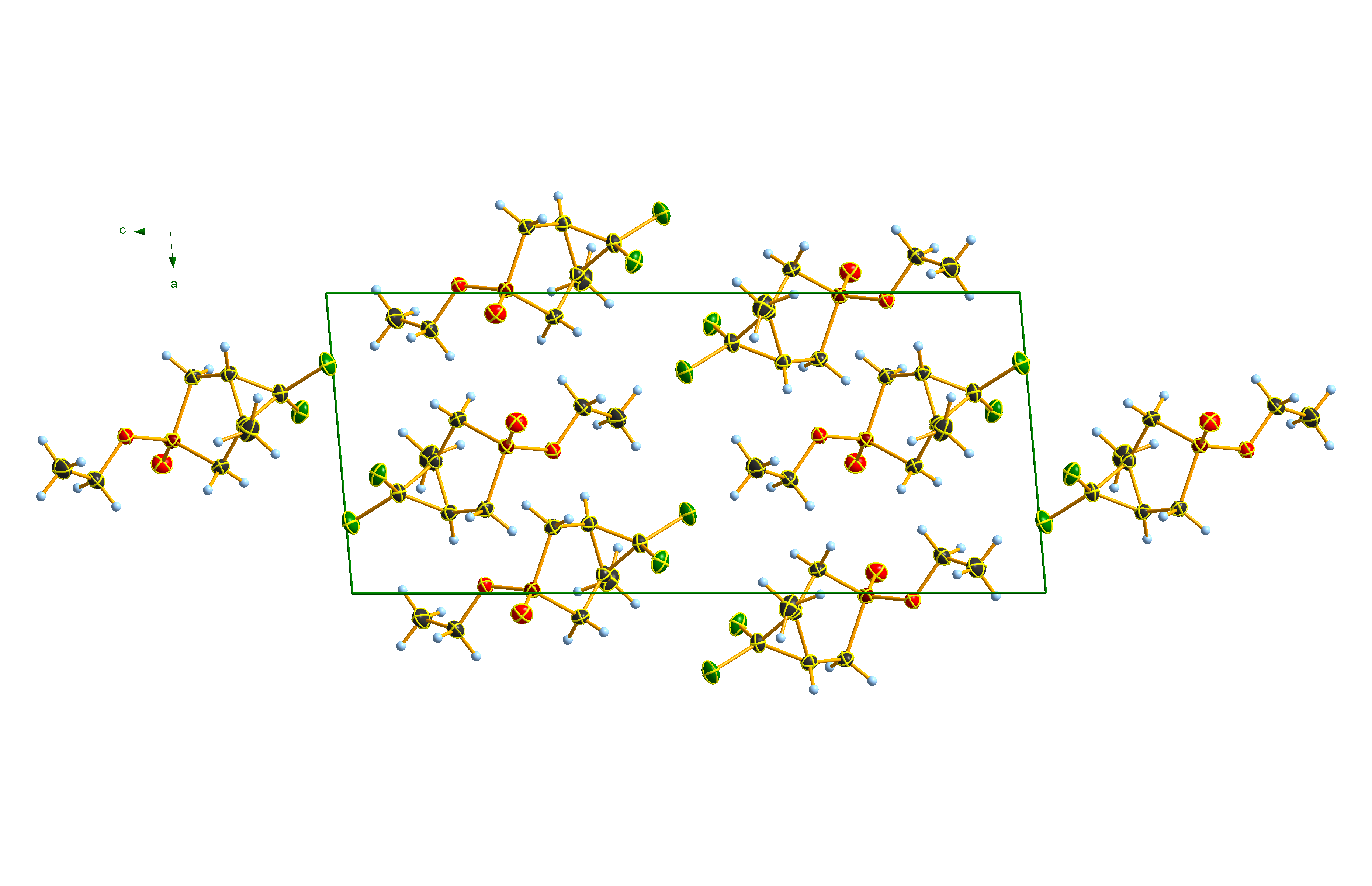


**Figure S2.** Crystal structure of phosphabicyclo[3.1.0]hexane 3-oxide **2Ab**, view of the unit cell along the *b*-axis. DIAMOND representation; thermal ellipsoids are drawn at 50 % probability level.


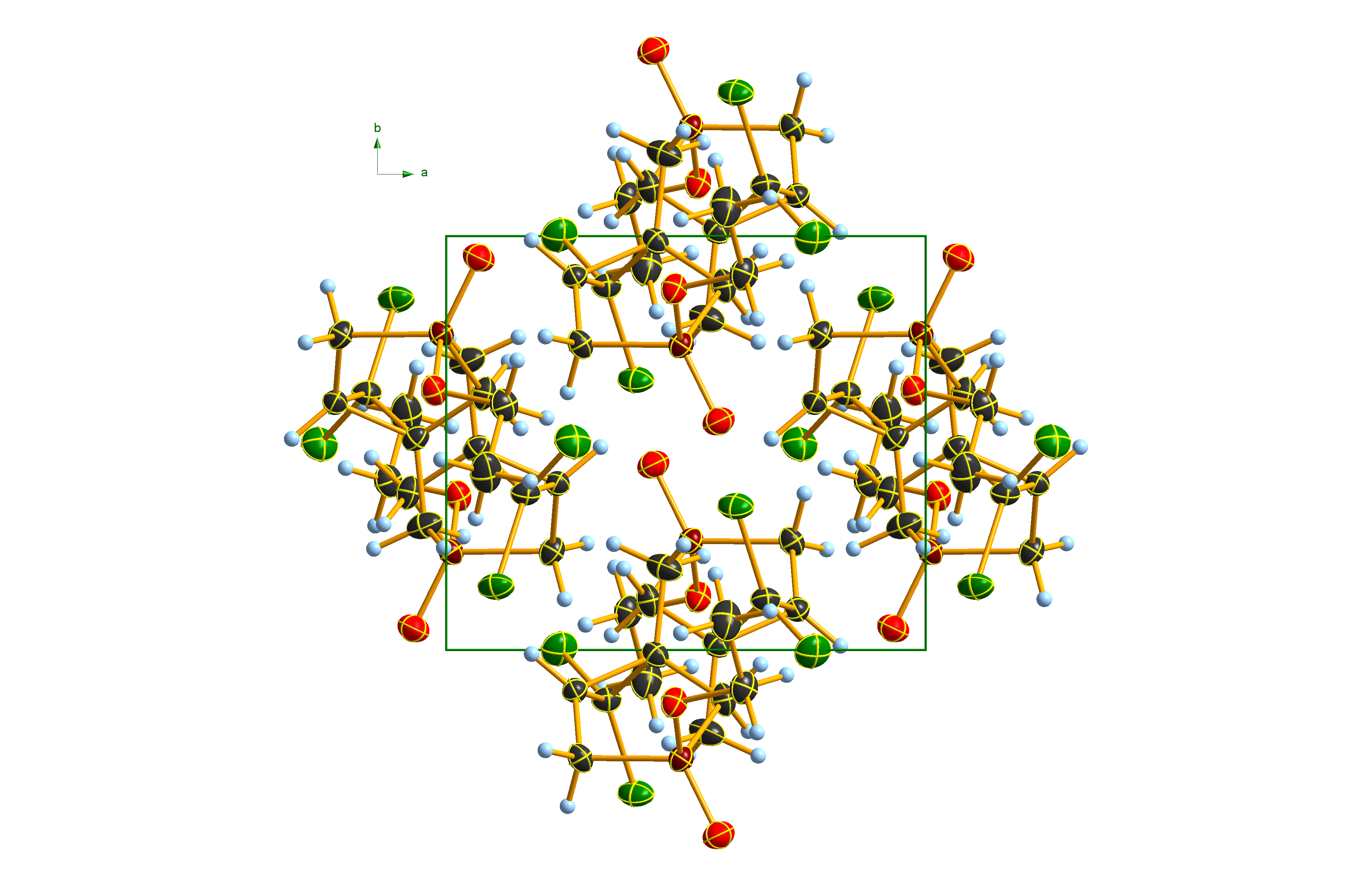


**Figure S3.** Crystal structure of phosphabicyclo[3.1.0]hexane 3-oxide **2Ab**, view of the unit cell along the *c*-axis. DIAMOND representation; thermal ellipsoids are drawn at 50 % probability level.


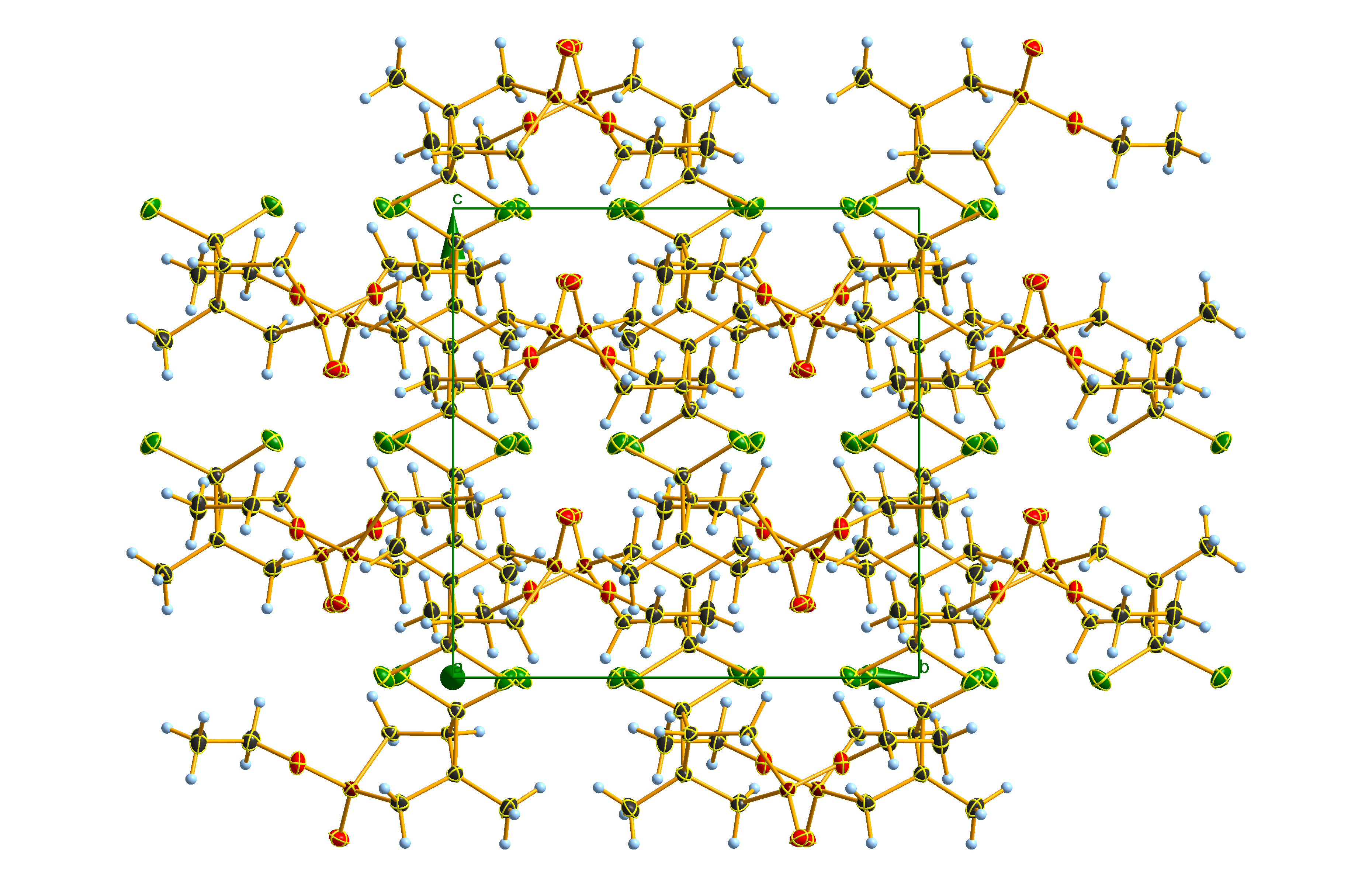


**Figure S4.** Crystal structure of phosphabicyclo[3.1.0]hexane 3-oxide **2Bb**; view of the unit cell along the *a*-axis. DIAMOND representation; thermal ellipsoids are drawn at 50 % probability level.


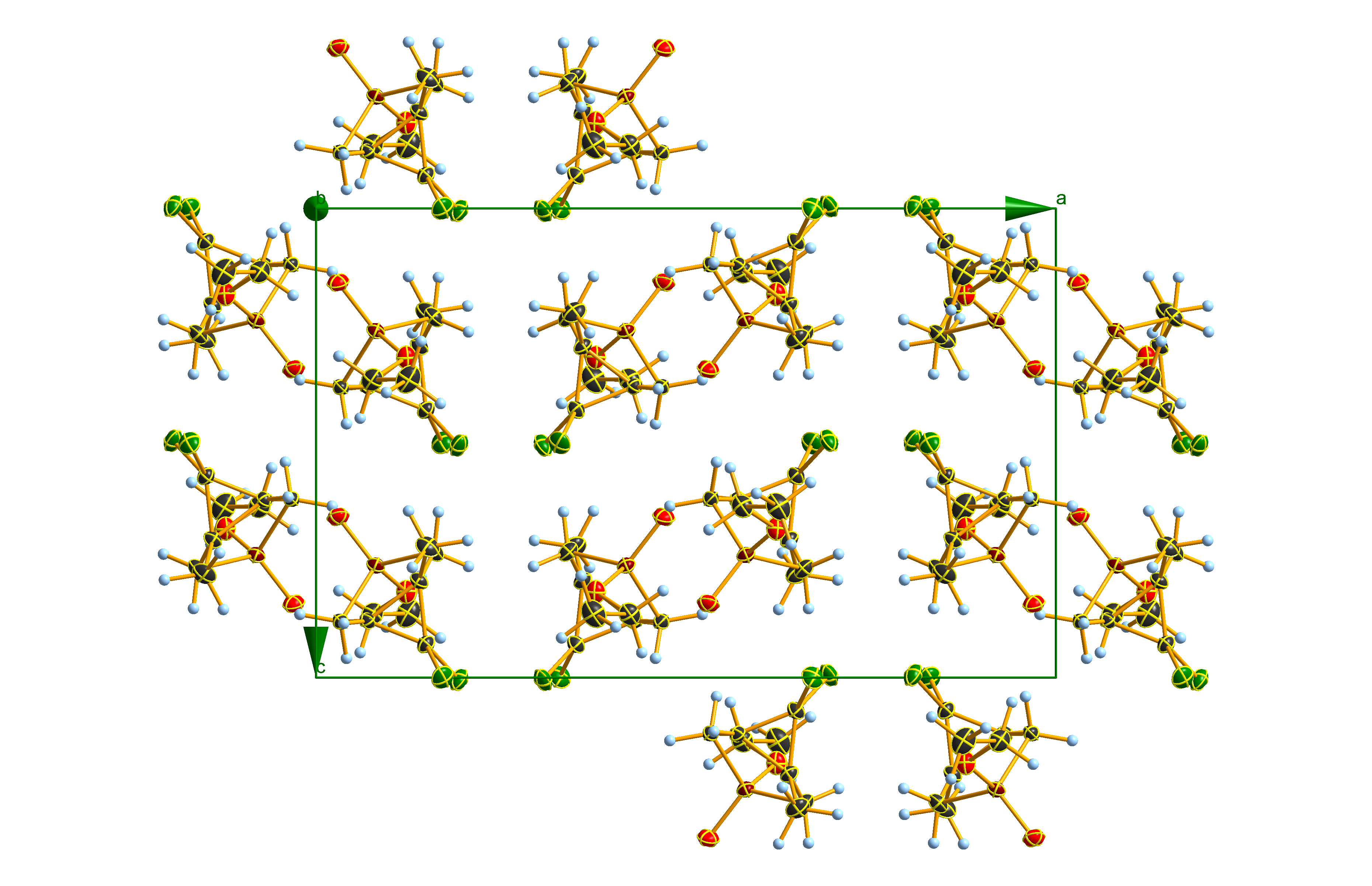


**Figure S5.** Crystal structure of phosphabicyclo[3.1.0]hexane 3-oxide **2Bb**; view of the unit cell along the *b*-axis. DIAMOND representation; thermal ellipsoids are drawn at 50 % probability level.


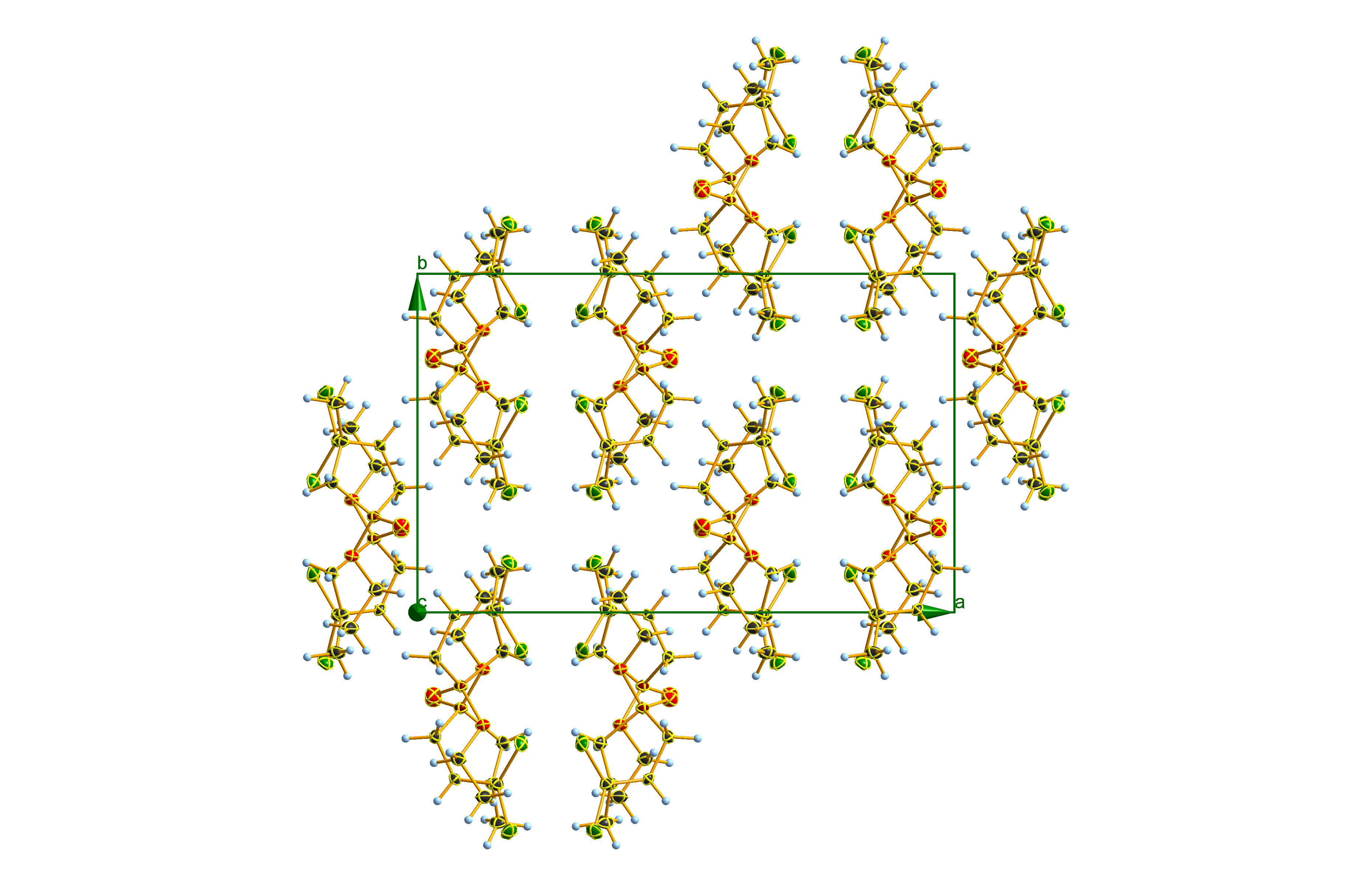
**Figure S6.** Crystal structure of phosphabicyclo[3.1.0]hexane 3-oxide **2Bb**; view of the unit cell along the *c*-axis. DIAMOND representation; thermal ellipsoids are drawn at 50 % probability level.

# ^31^P, ^13^C, ^1^H NMR spectra for the compounds 2a, 2Ab, 2bB, 2d-e, 3a-e, 4Aa-e, 5Ab, 5Ac and 5Ae synthesized

**
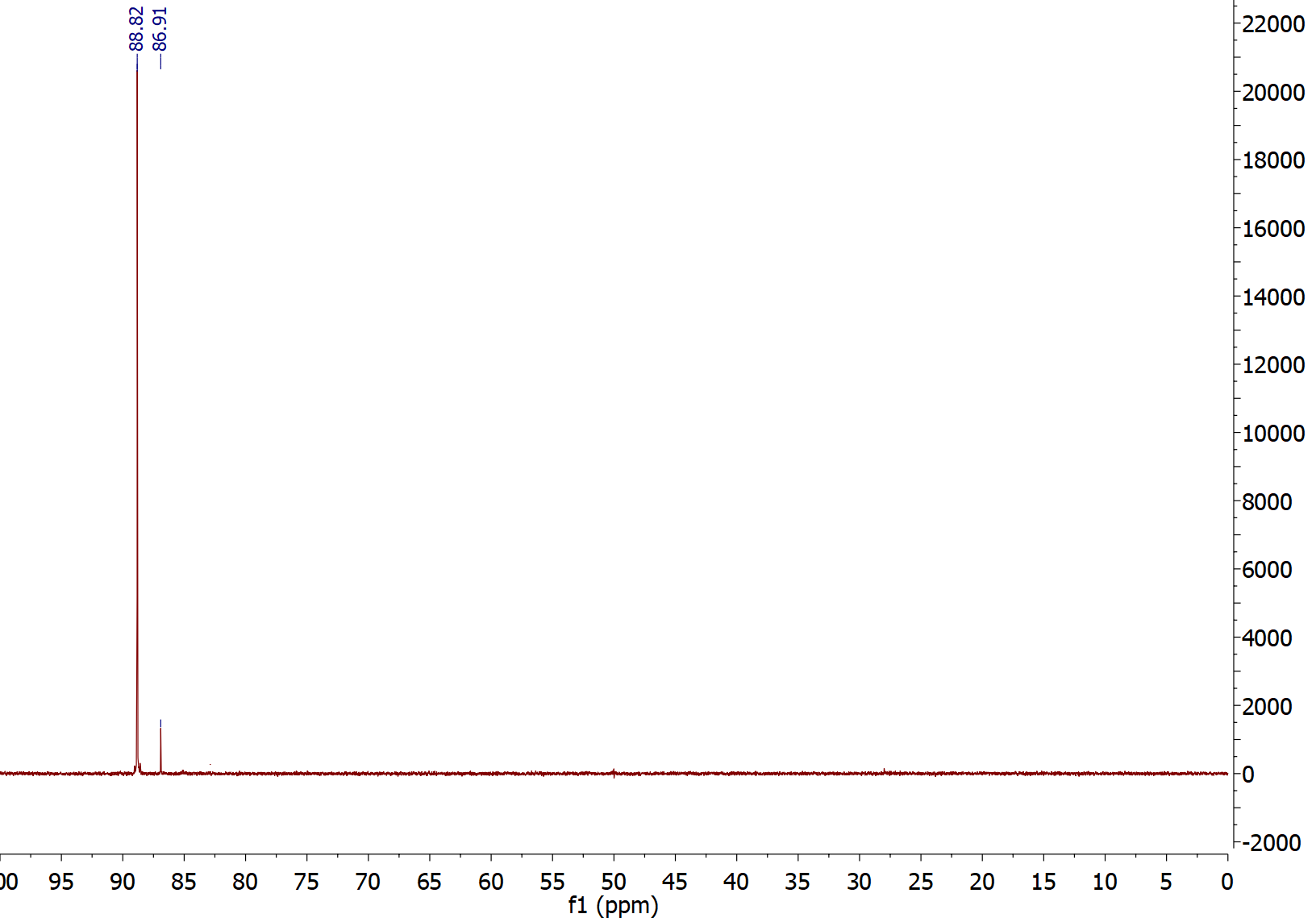
****Figure S7. ^31^P {^1^H} NMR (202 MHz, CDCl_3_) spectra for compound 2Aa and 2Ba**

**Figure S8. ^31^P {^1^H} NMR (122 MHz, CDCl_3_) spectra for compound 2Ab**

**
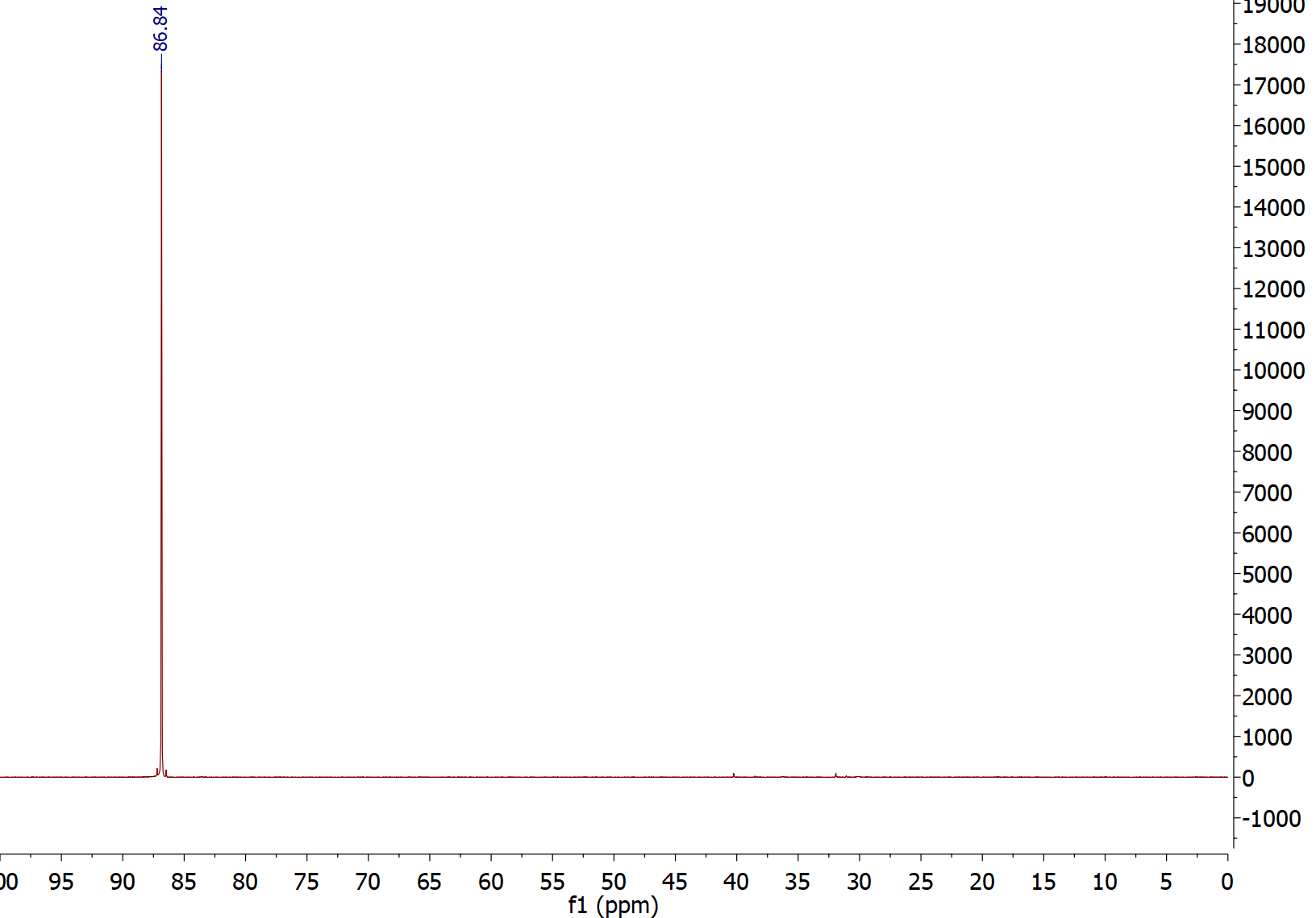
**

**Figure S9. ^13^C {^1^H} NMR (75 MHz, CDCl_3_) spectra for compound 2Ab**

**
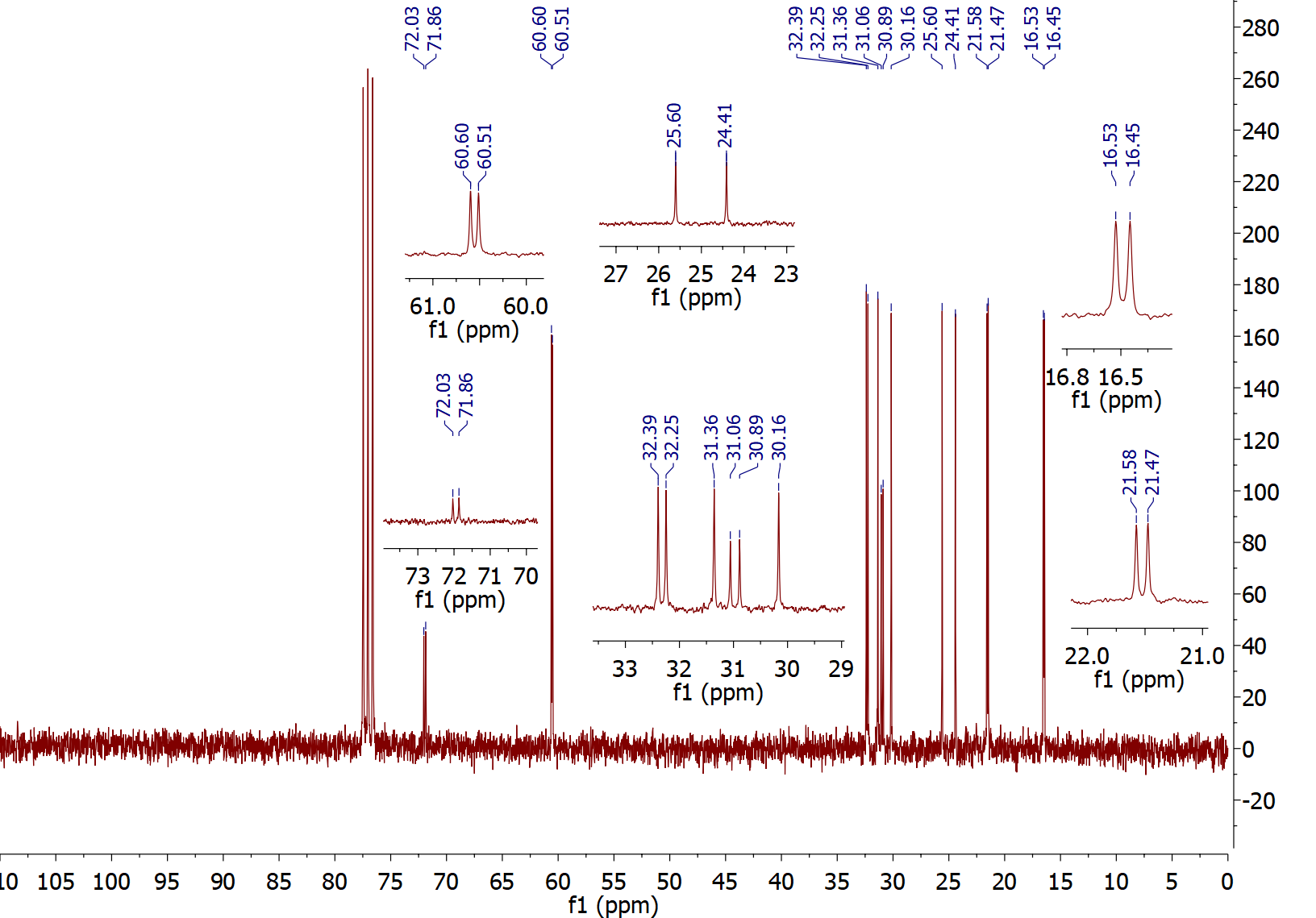
**

**Figure S10. ^1^H NMR (300 MHz, CDCl_3_) spectra for compound 2Ab**

**
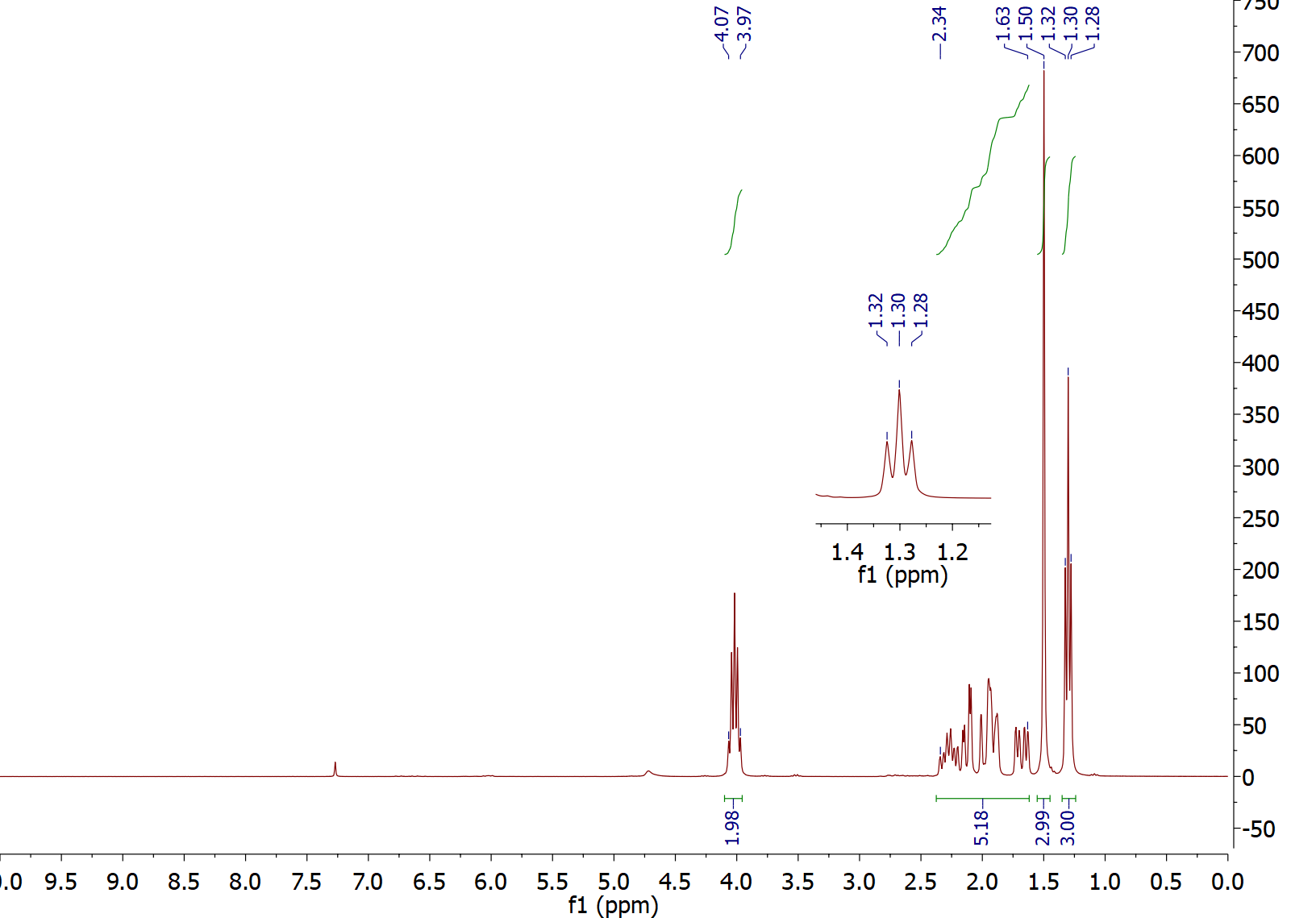
**

**Figure S11. ^31^P {^1^H} NMR (202 MHz, CDCl_3_) spectra for compound 2Bb**

**
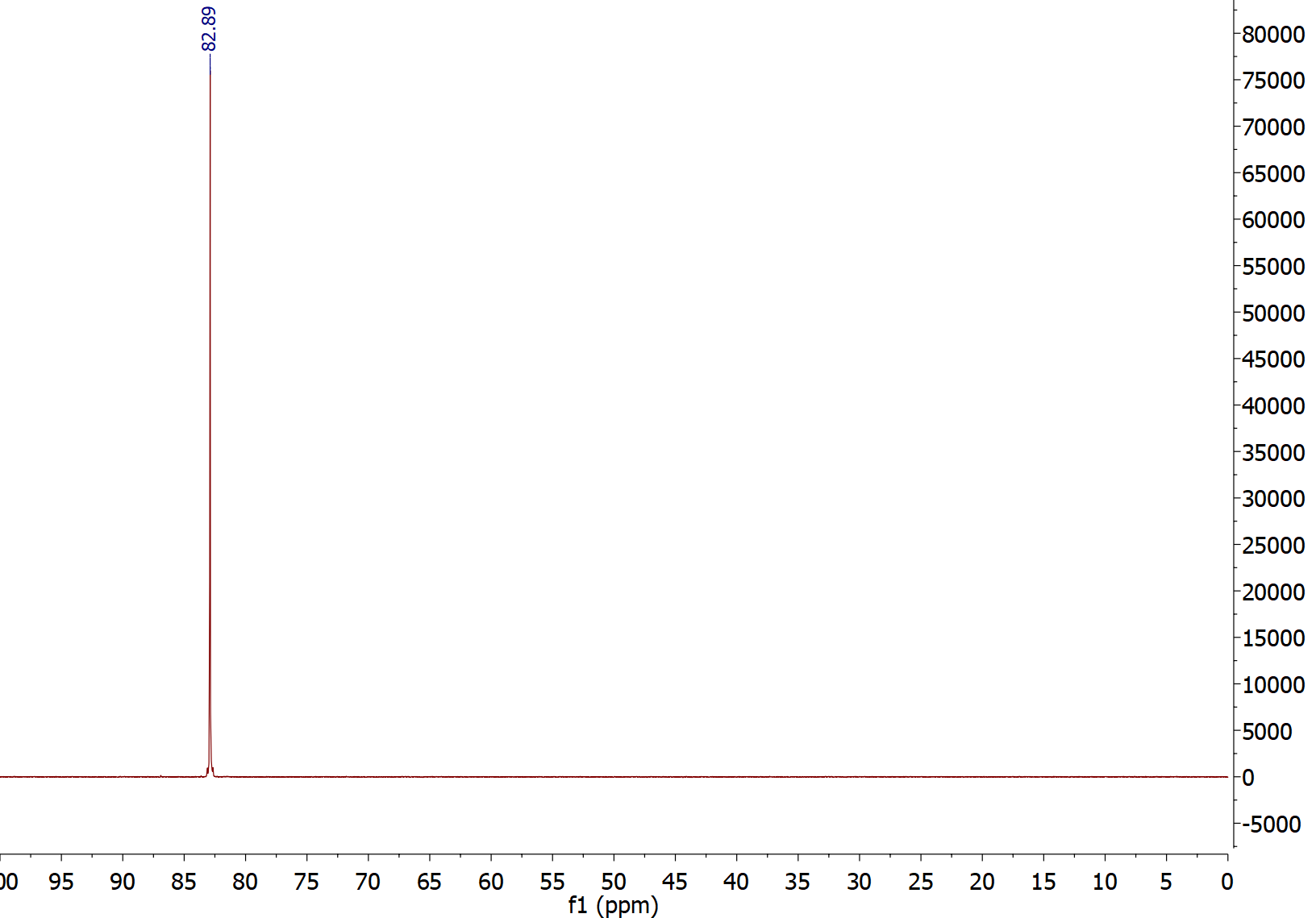
**

**Figure S12. ^13^C {^1^H} NMR (75 MHz, CDCl_3_) spectra for compound 2Bb**

**
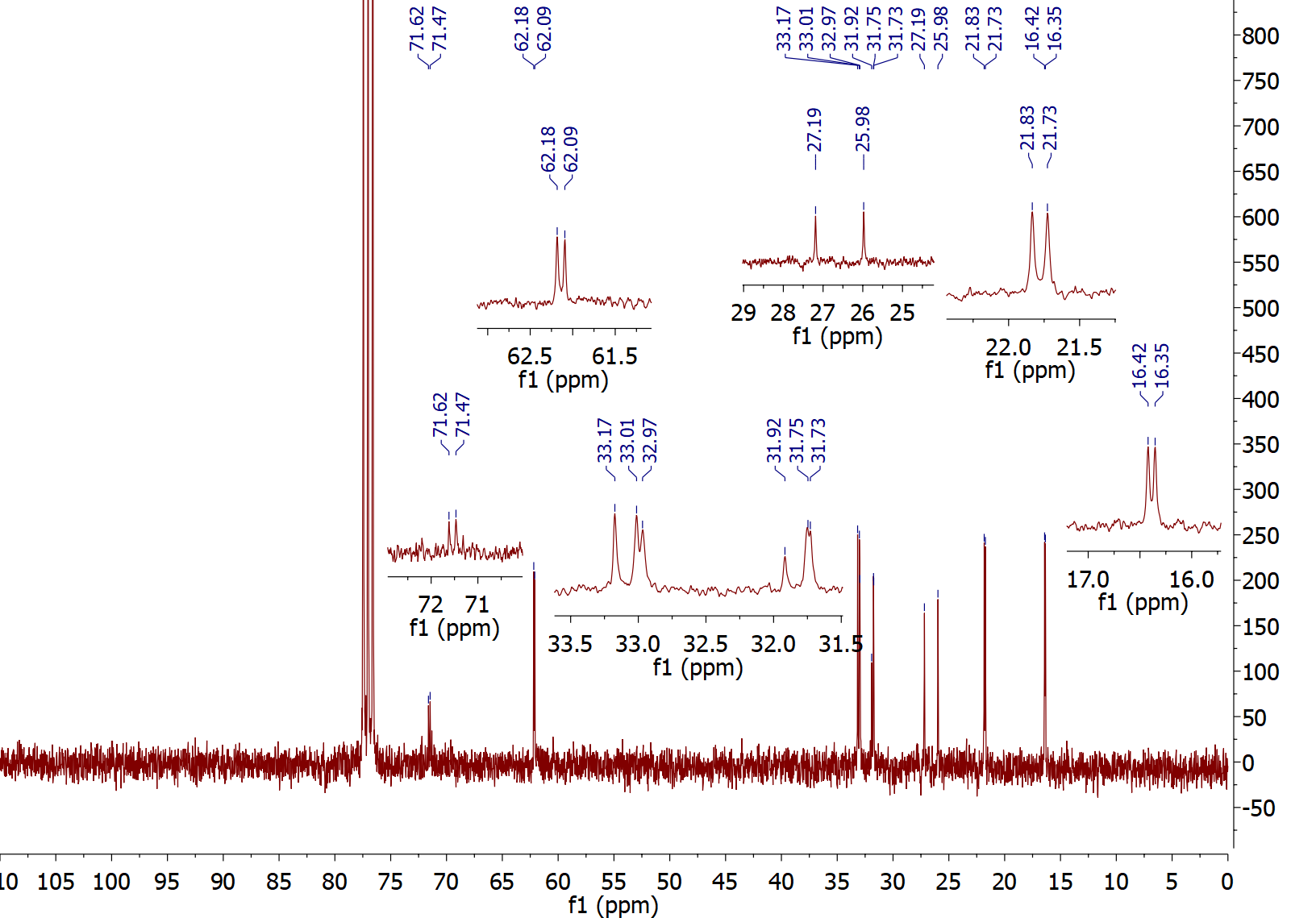
**

**Figure S13. ^1^H NMR (300 MHz, CDCl_3_) spectra for compound 2Bb**

**
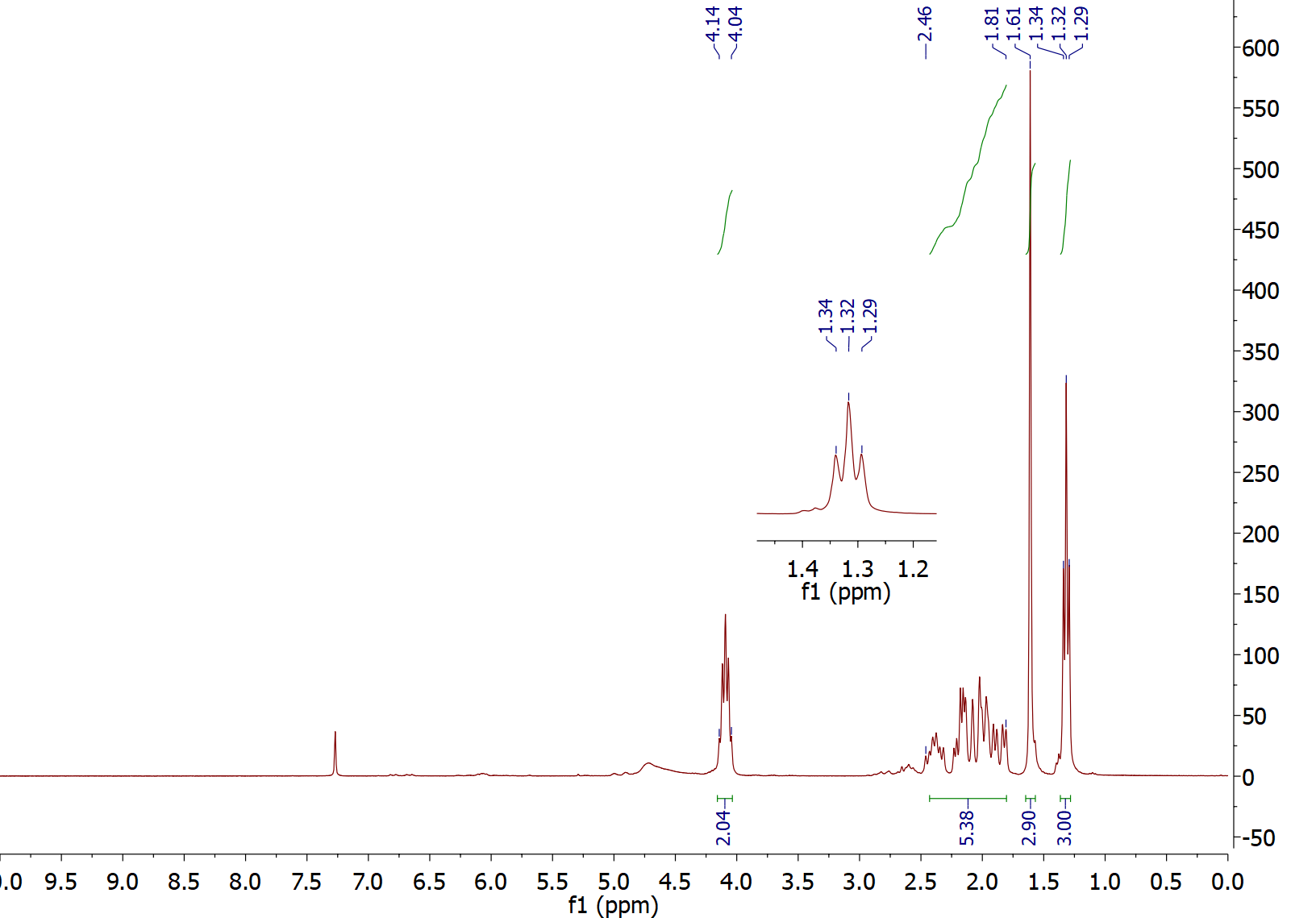
**

**Figure S14. ^31^P {^1^H} NMR (202 MHz, CDCl_3_) spectra for compound 2Ac and 2Bc**

**
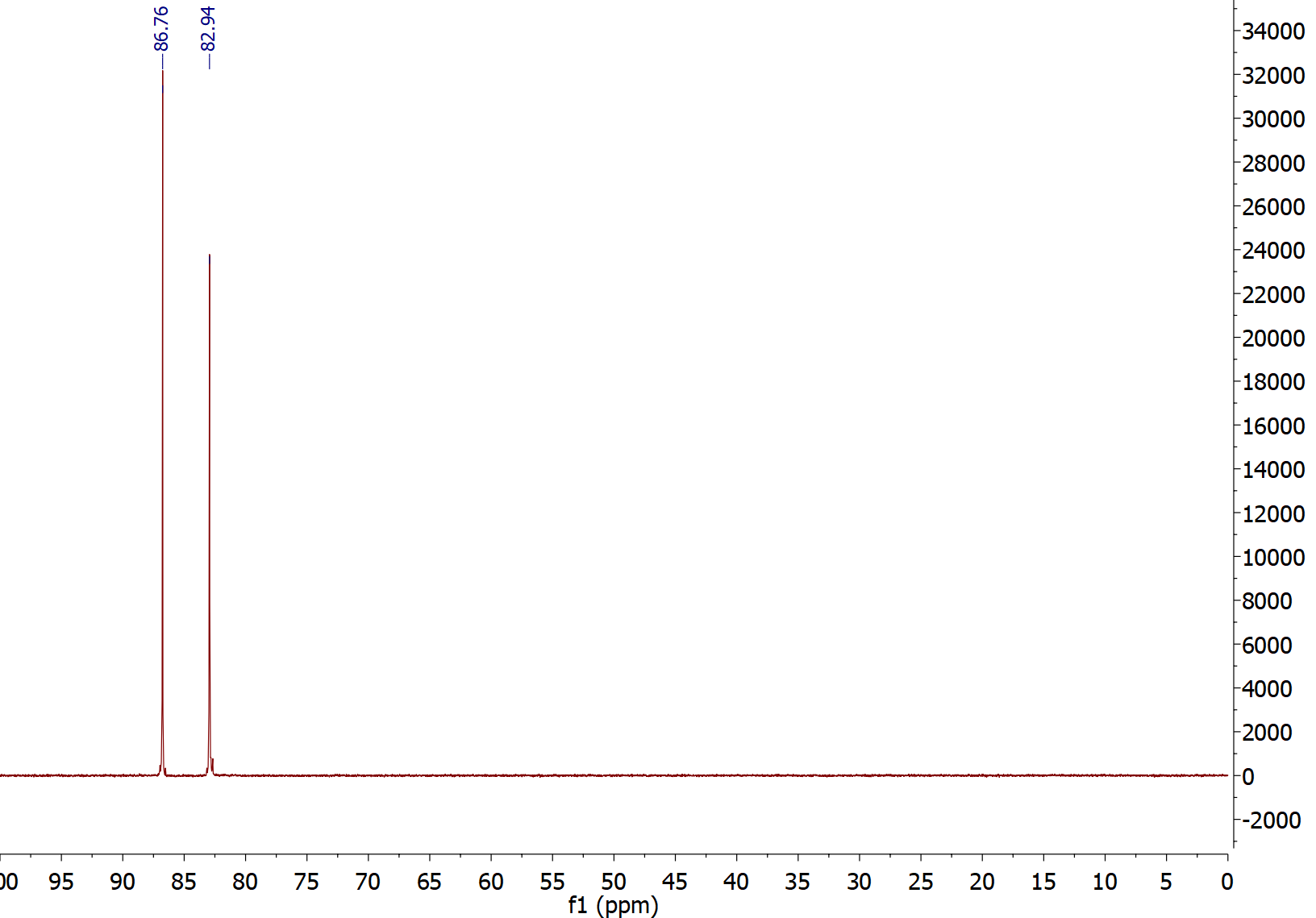
**

**Figure S15. ^31^P {^1^H} NMR (122 MHz, CDCl_3_) spectra for compound 2Ad and 2Bd**

**
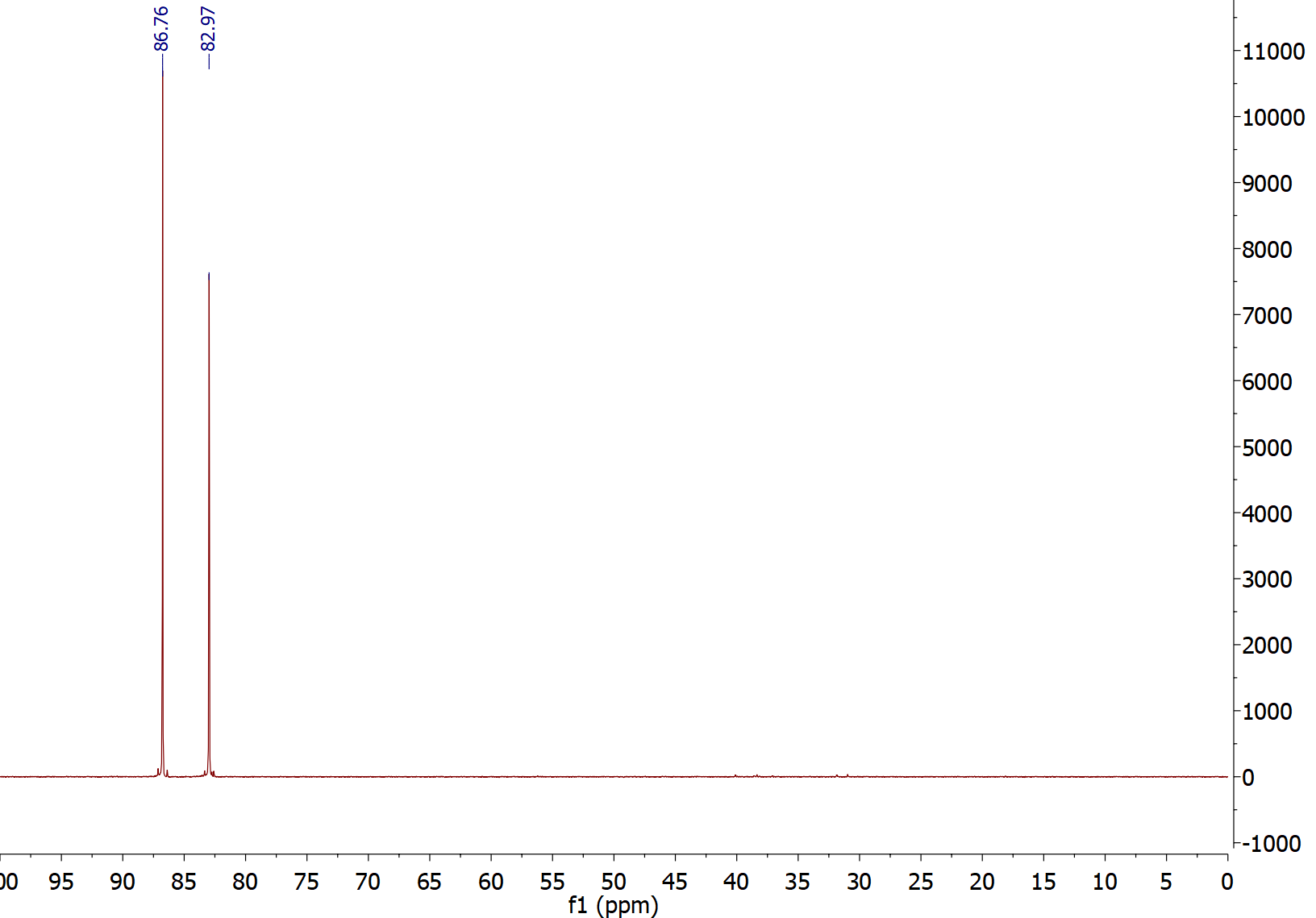
**

**Figure S16. ^13^C {^1^H} NMR (75 MHz, CDCl_3_) spectra for compound 2Ad and 2Bd**

**
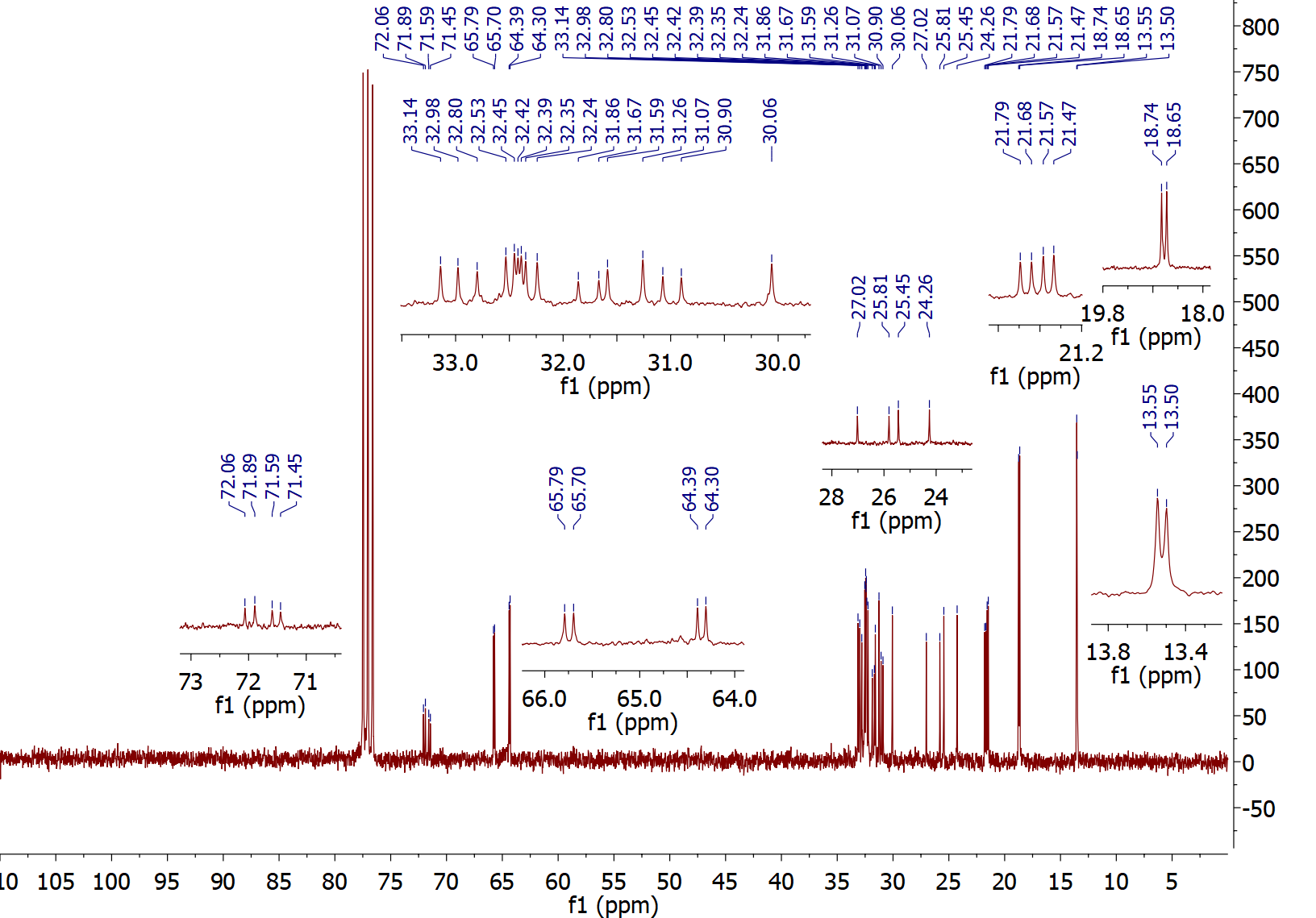
**

**Figure S17. ^1^H NMR (300 MHz, CDCl_3_) spectra for compound 2Ad and 2Bd**

**
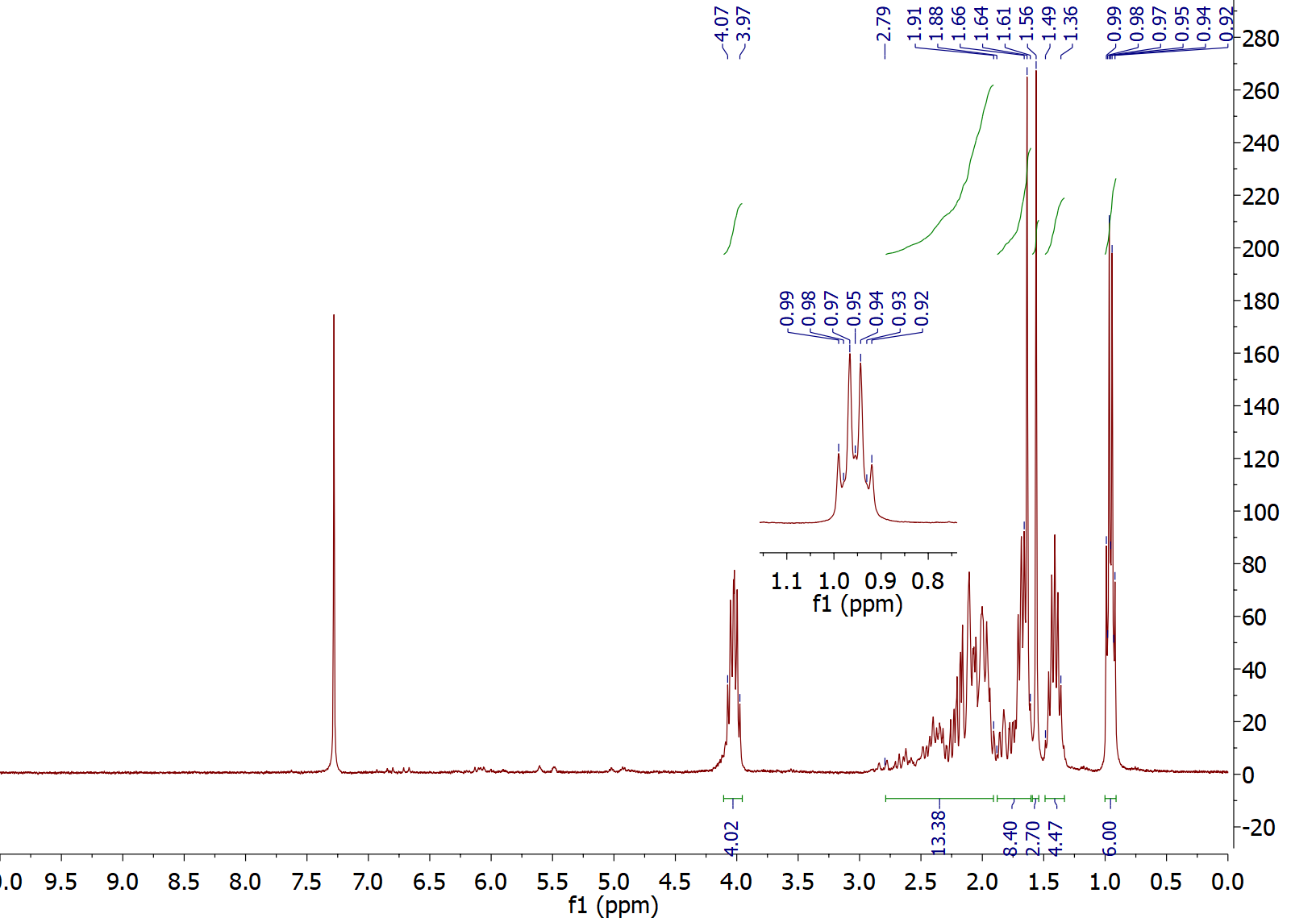
**

**Figure S18. ^31^P {^1^H} NMR (122 MHz, CDCl_3_) spectra for compound 2Be**

**
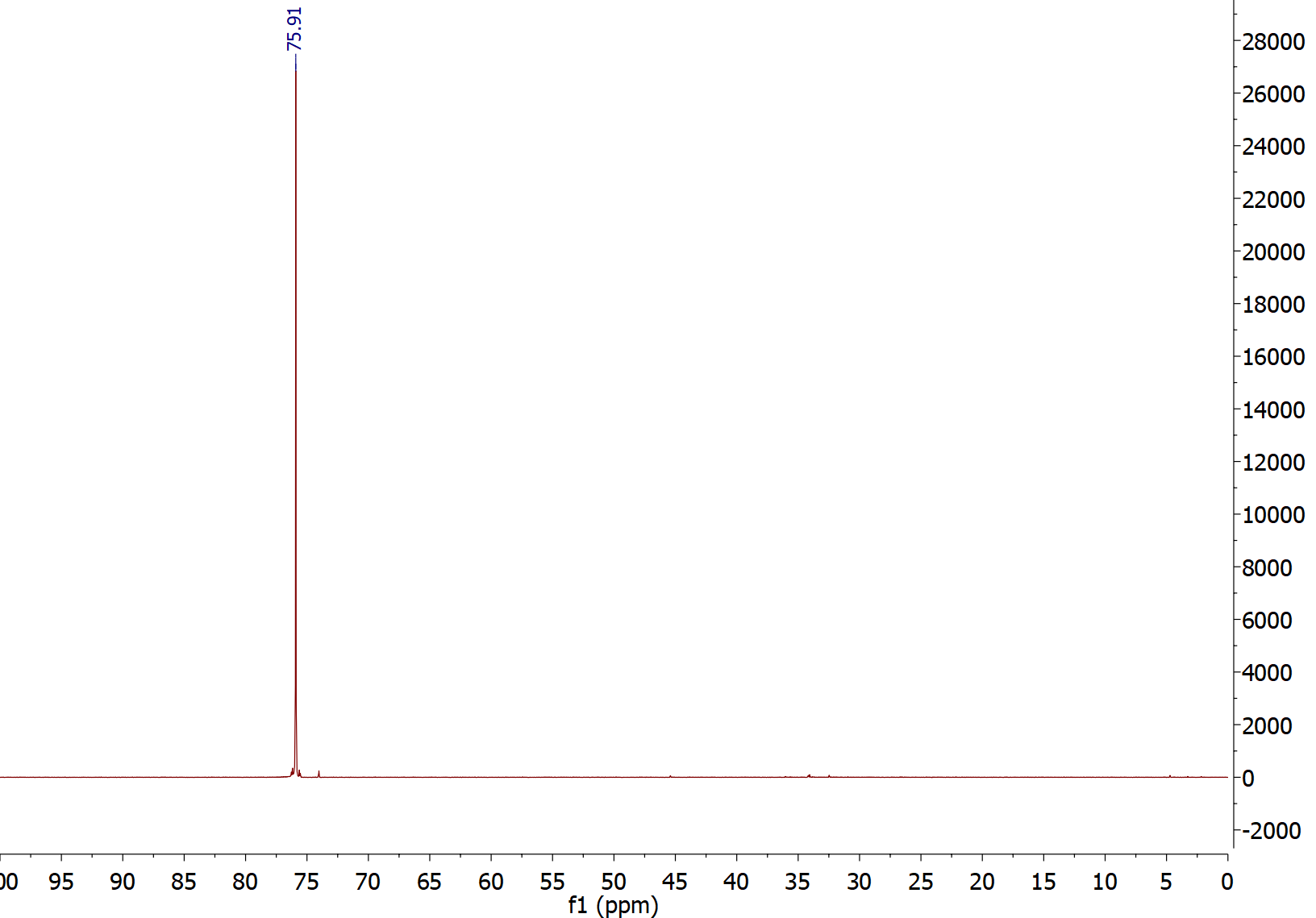
**

**Figure S19. ^31^P {^1^H} NMR (122 MHz, CDCl_3_) spectra for compound 3Aa and 3Ba**

**
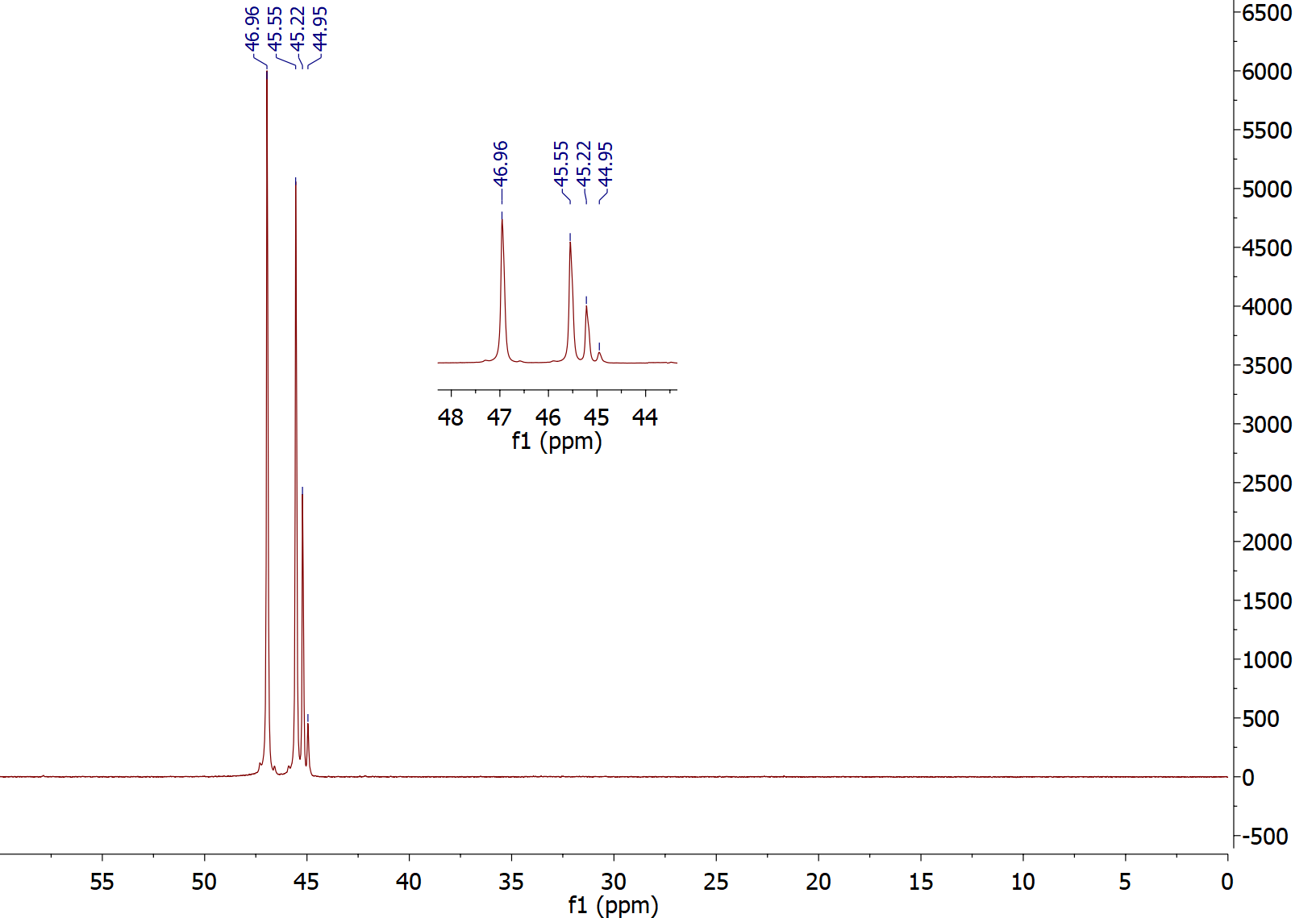
**

**Figure S20. ^31^P {^1^H} NMR (202 MHz, CDCl_3_) spectra for compound 3Ab and 3Bb**

**
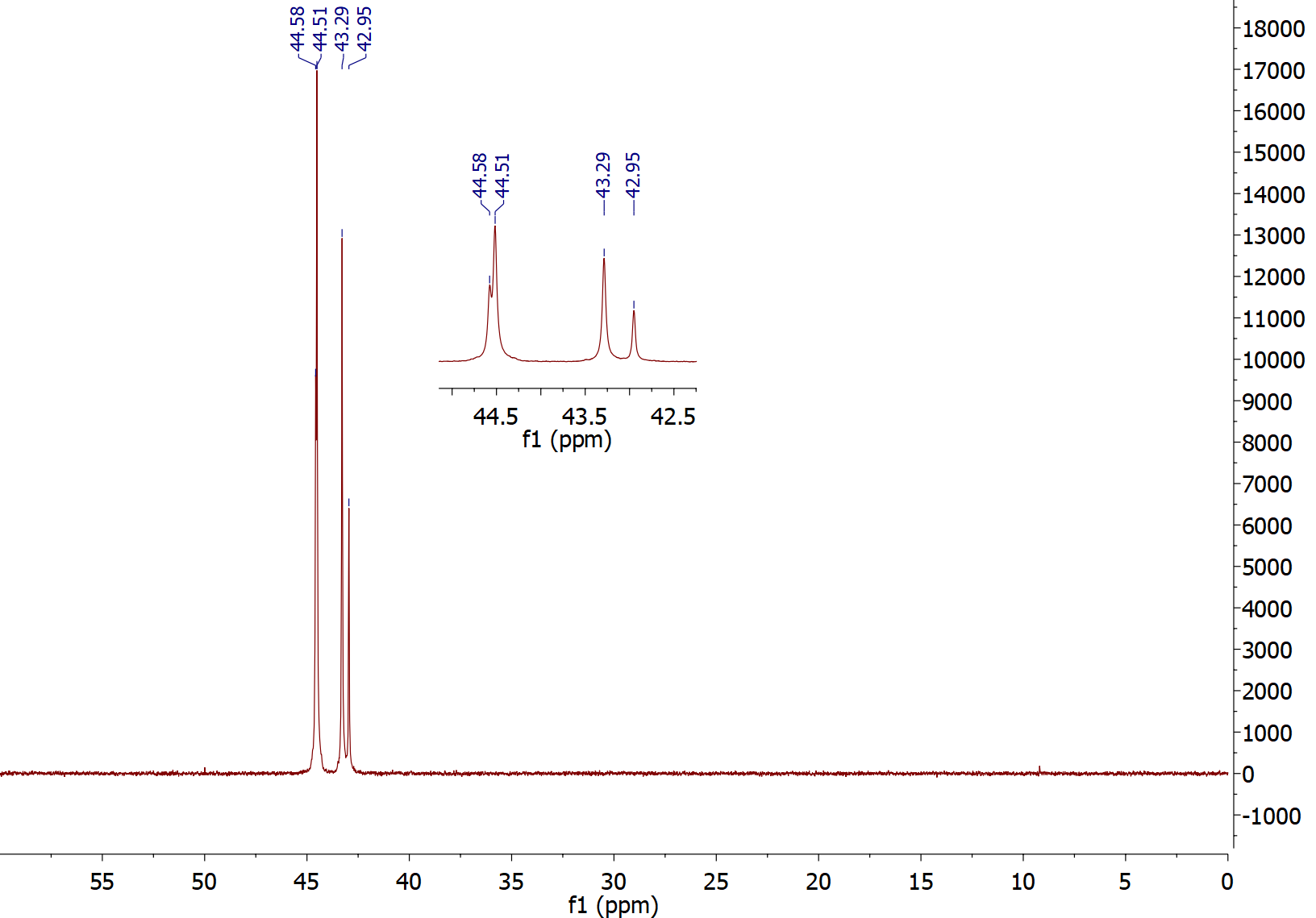
**

**Figure S21. ^31^P {^1^H} NMR (202 MHz, CDCl_3_) spectra for compound 3Ac and 3Bc**


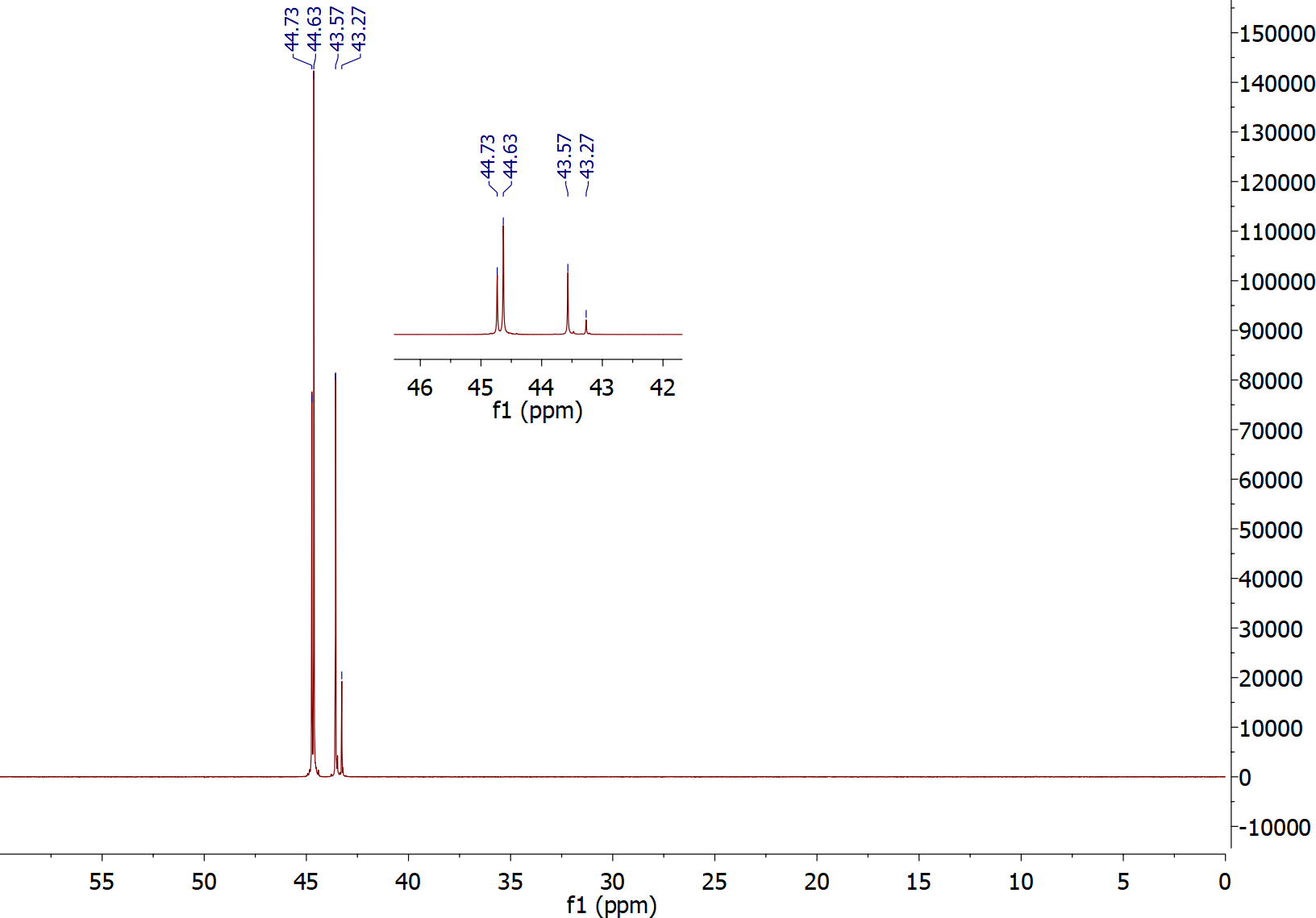


**Figure S22. ^31^P {^1^H} NMR (122 MHz, CDCl_3_) spectra for compound 3Ad and 3Bd**


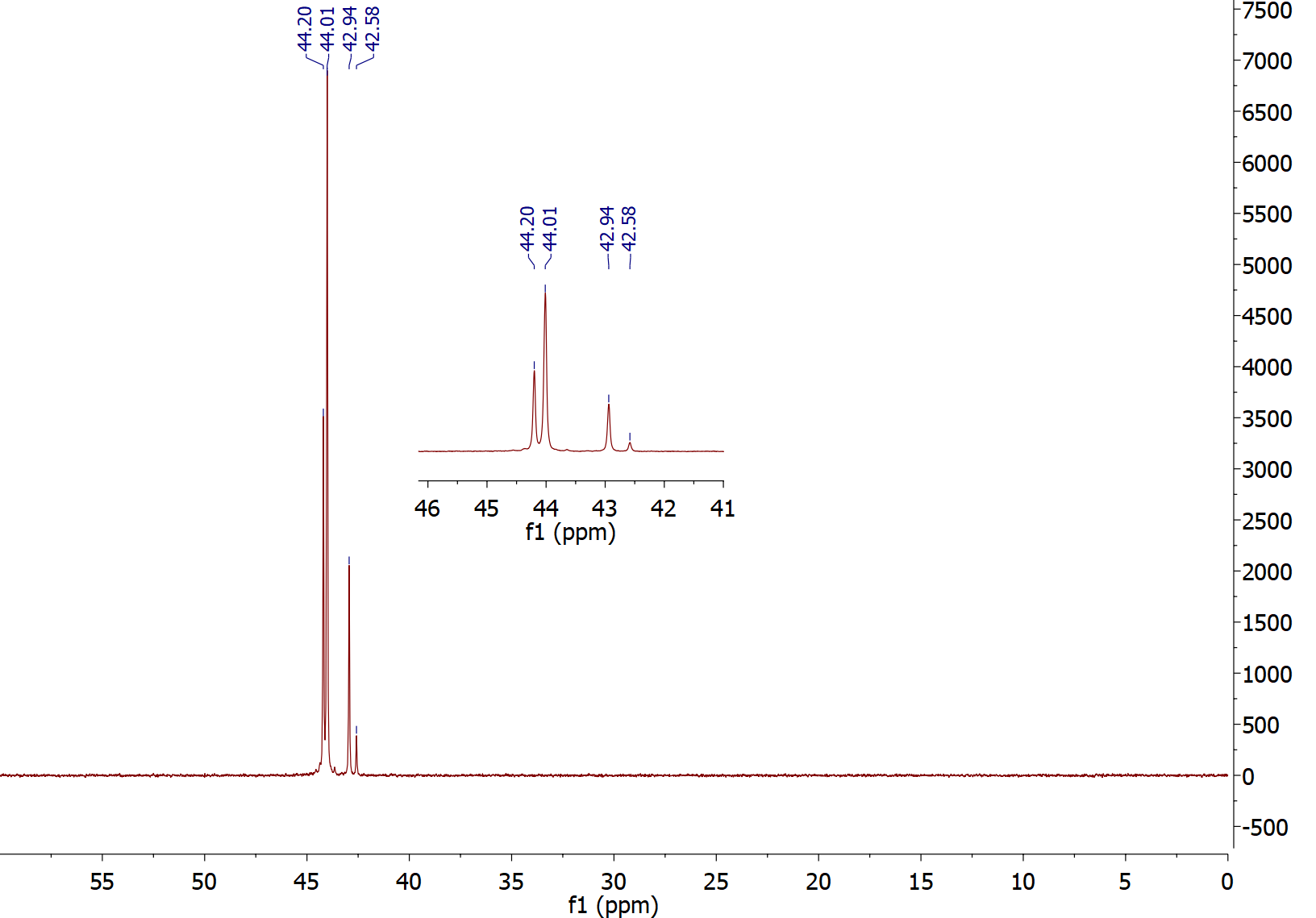


**Figure S23. ^13^C {^1^H} NMR (75 MHz, CDCl_3_) spectra for compound 3Ad and 3Bd**


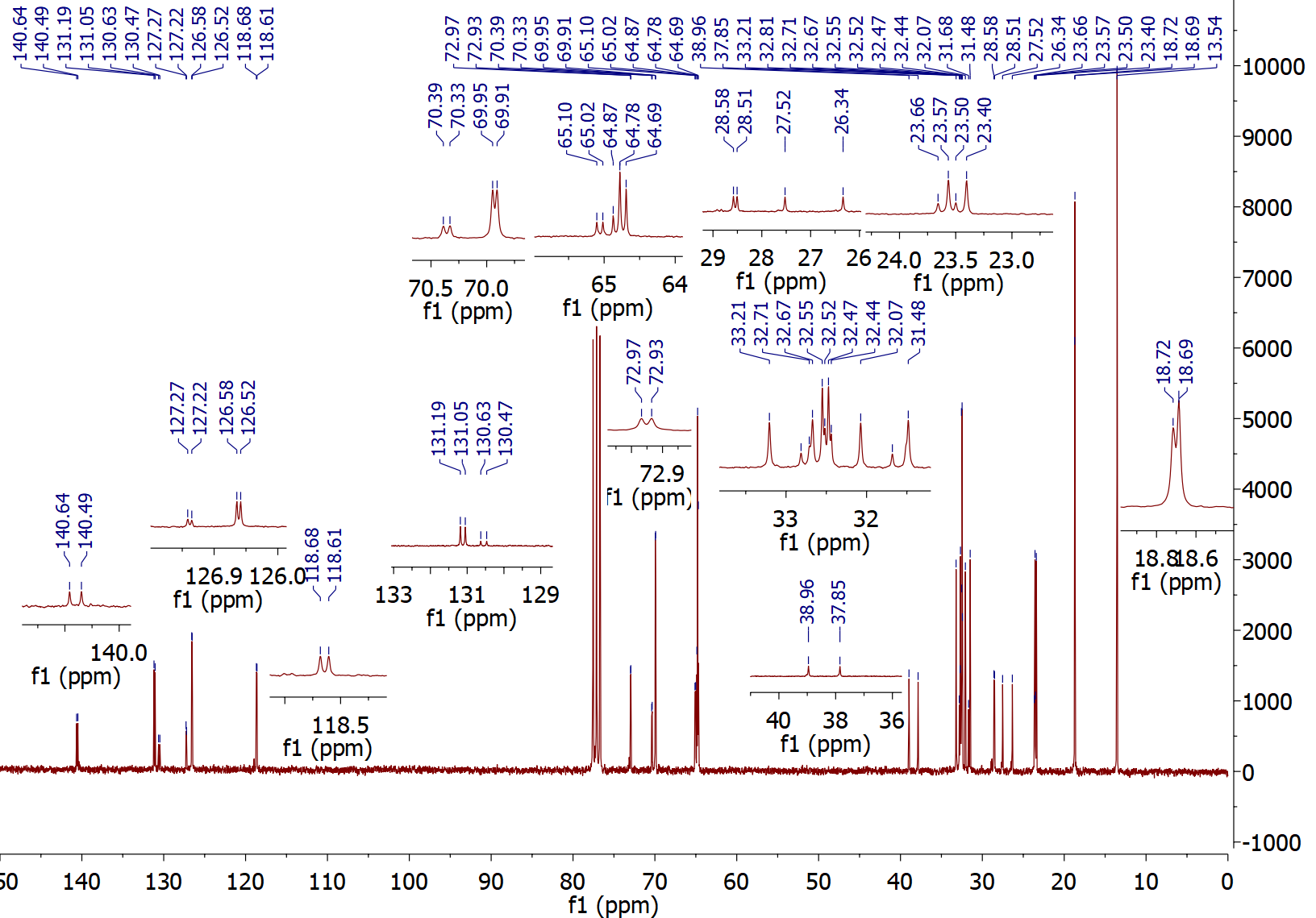


**Figure S24. ^1^H NMR (300 MHz, CDCl_3_) spectra for compound 3Ad and 3Bd**


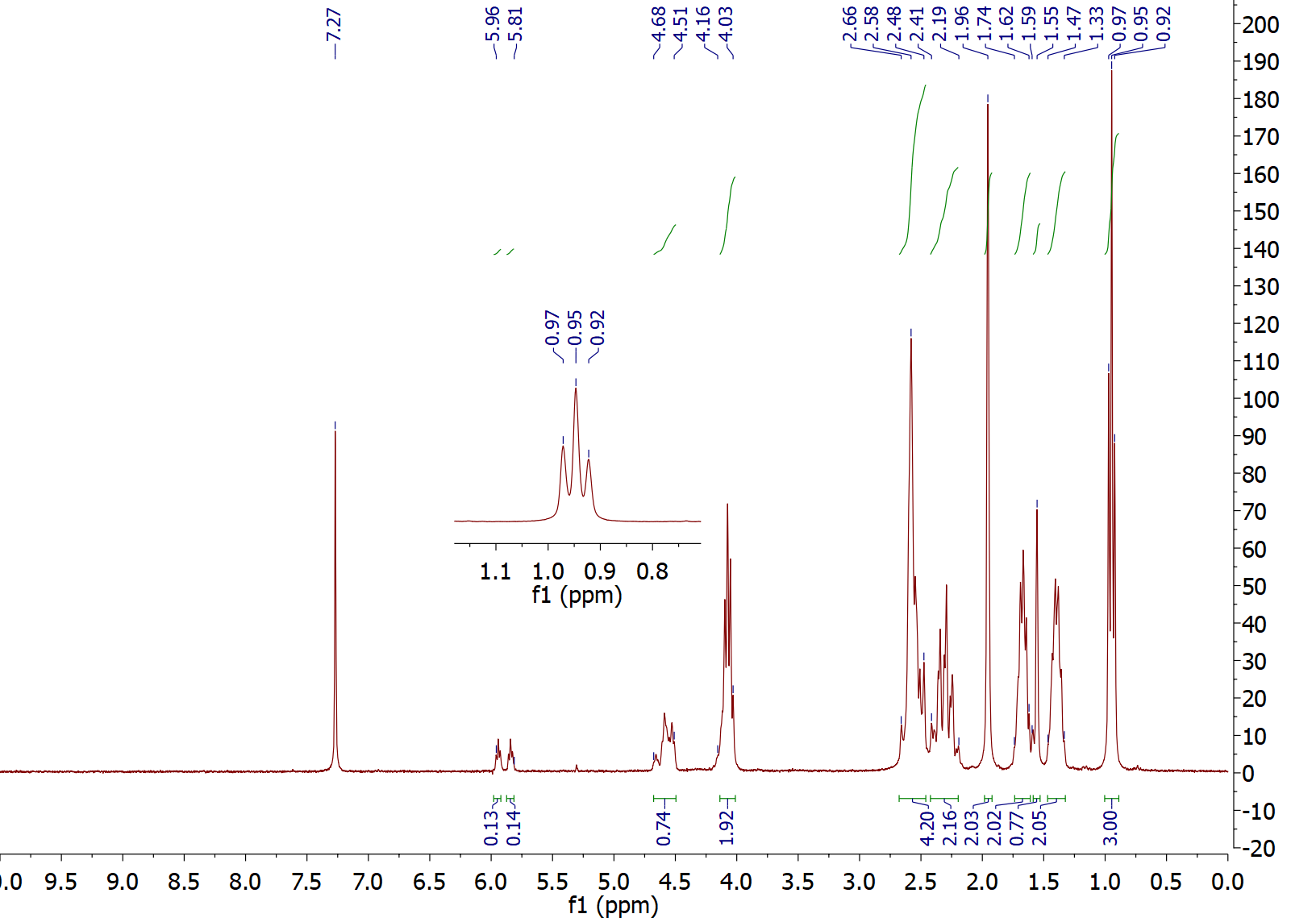


**Figure S25. ^31^P {^1^H} NMR (122 MHz, CDCl_3_) spectra for compound 3Ae and 3Be**


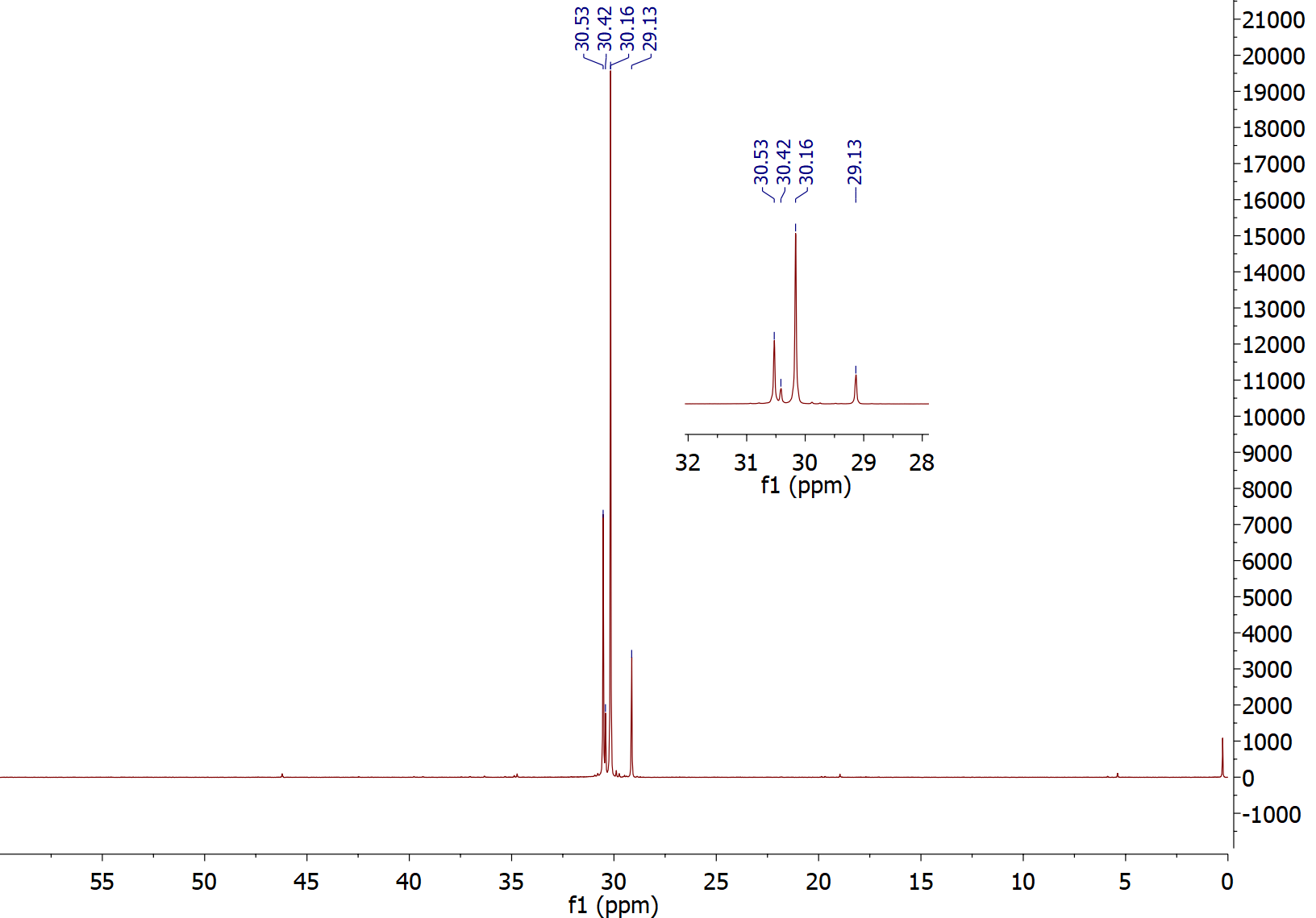


**Figure S26. ^31^P {^1^H} NMR (122 MHz, CDCl_3_) spectra for compound 4Aa**


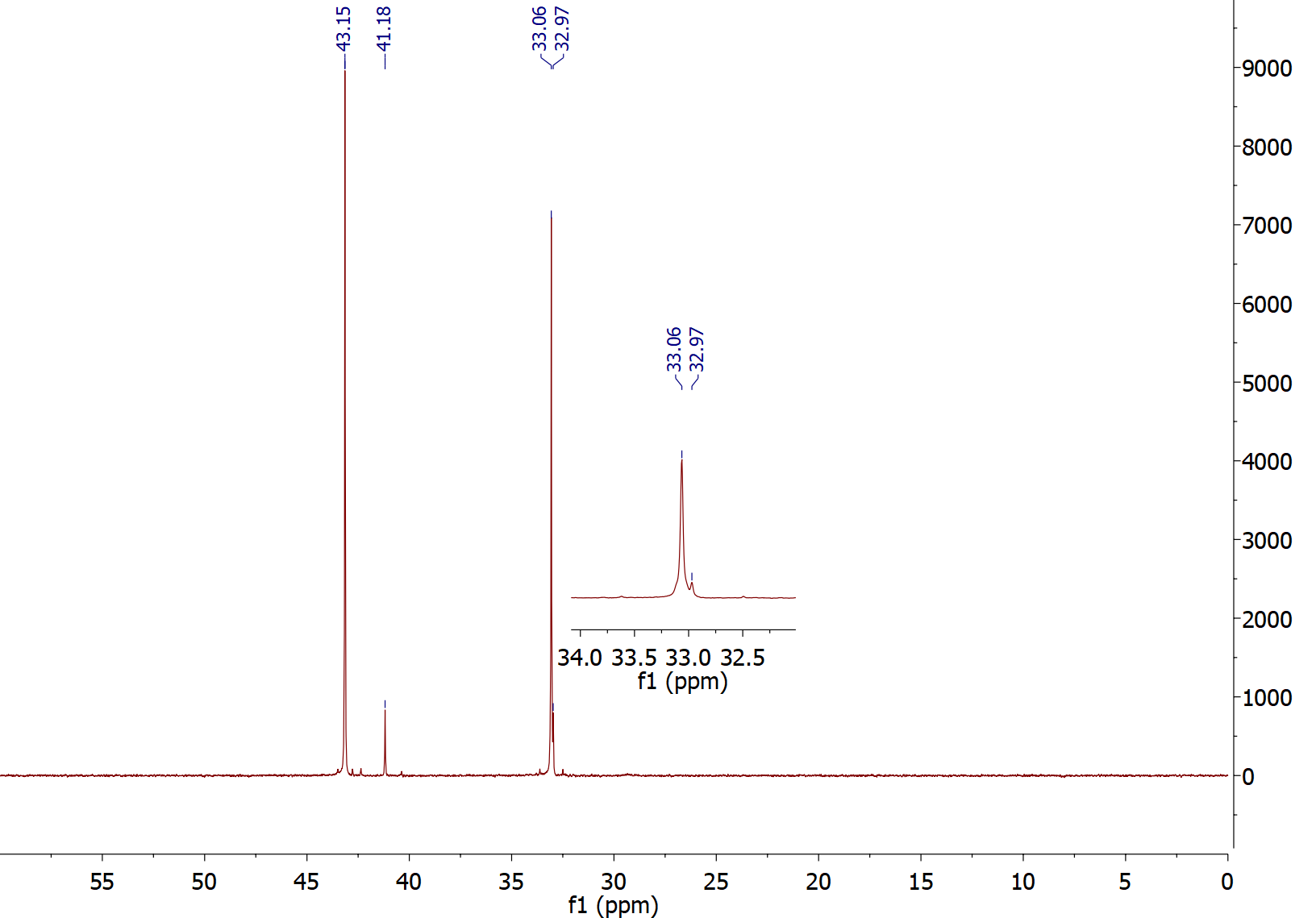


**Figure S27. ^13^C {^1^H} NMR (75 MHz, CDCl_3_) spectra for compound 4Aa**


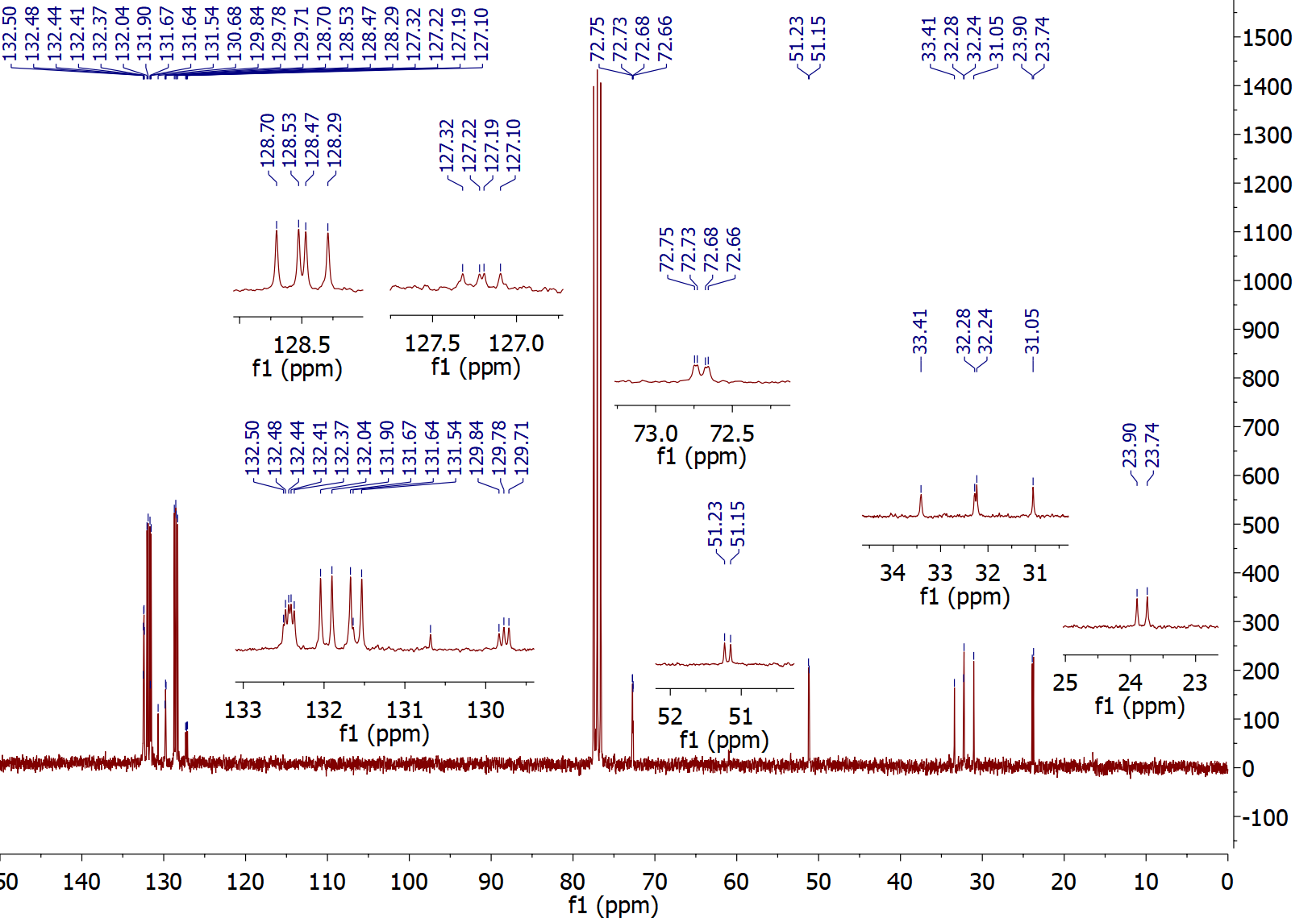


**Figure S28. ^1^H NMR (300 MHz, CDCl_3_) spectra for compound 4Aa**


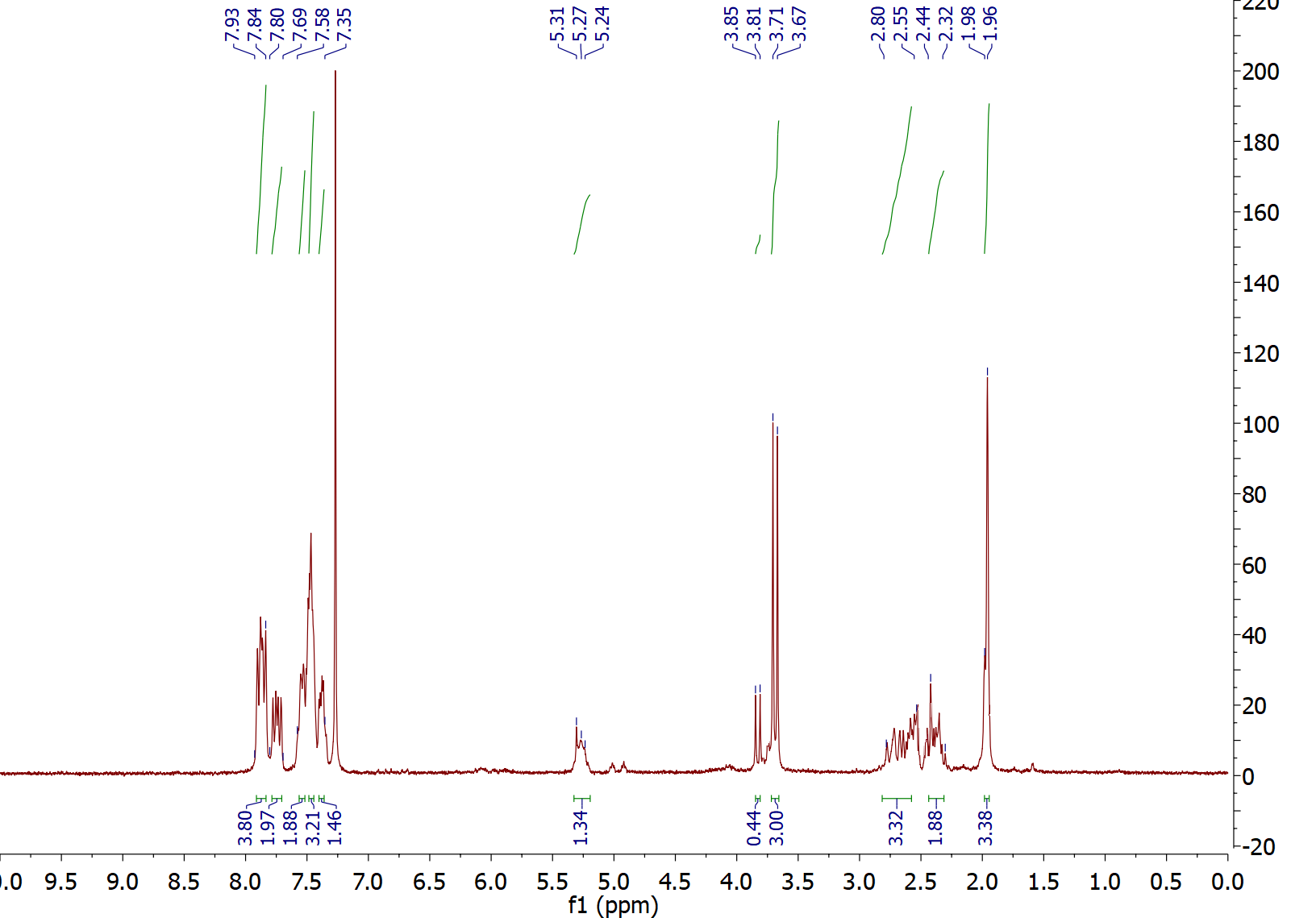


**Figure S29. ^31^P {^1^H} NMR (202 MHz, CDCl_3_) spectra for compound 4Ab**


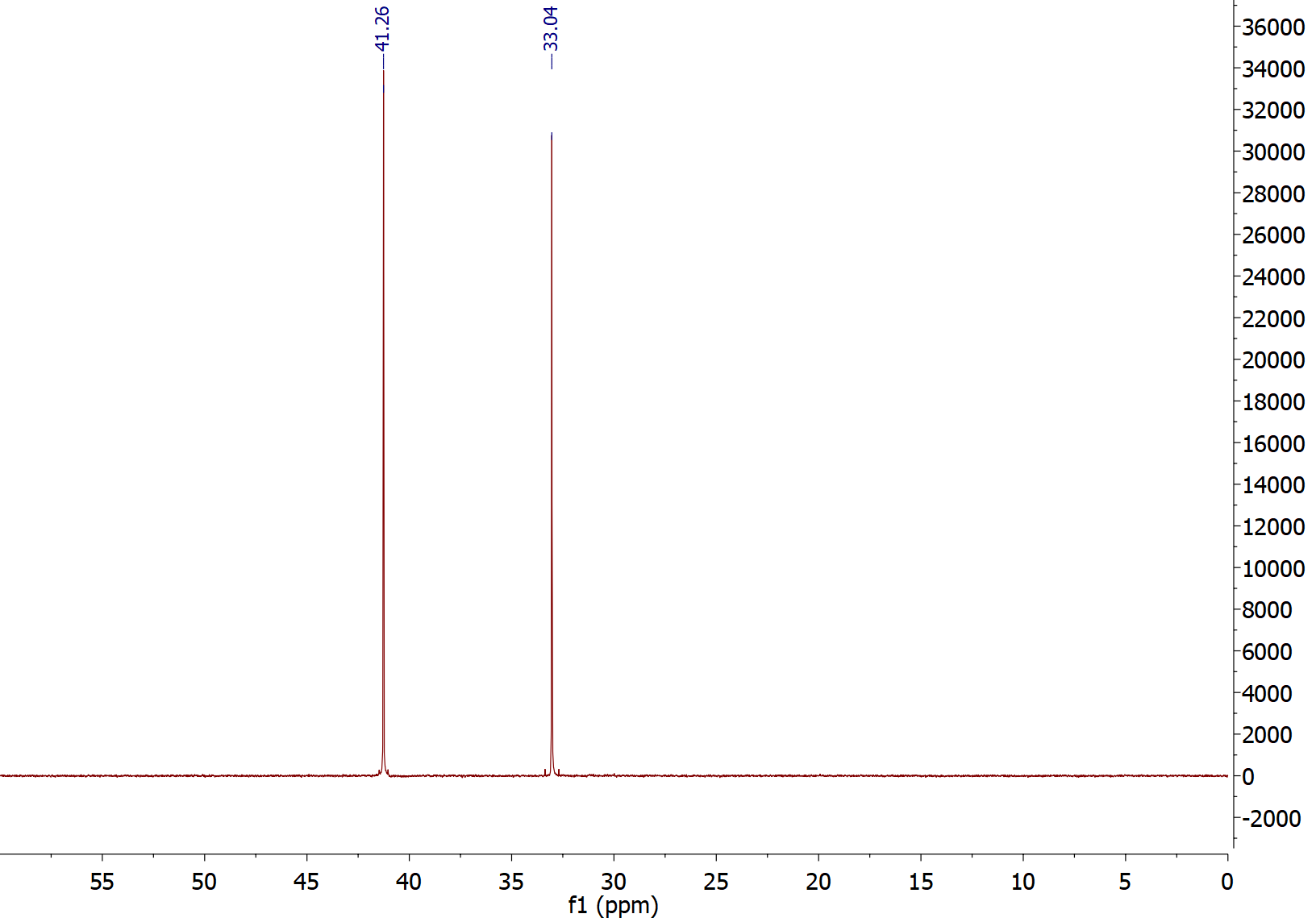


**Figure S30. ^13^C {^1^H} NMR (126 MHz, CDCl_3_) spectra for compound 4Ab**


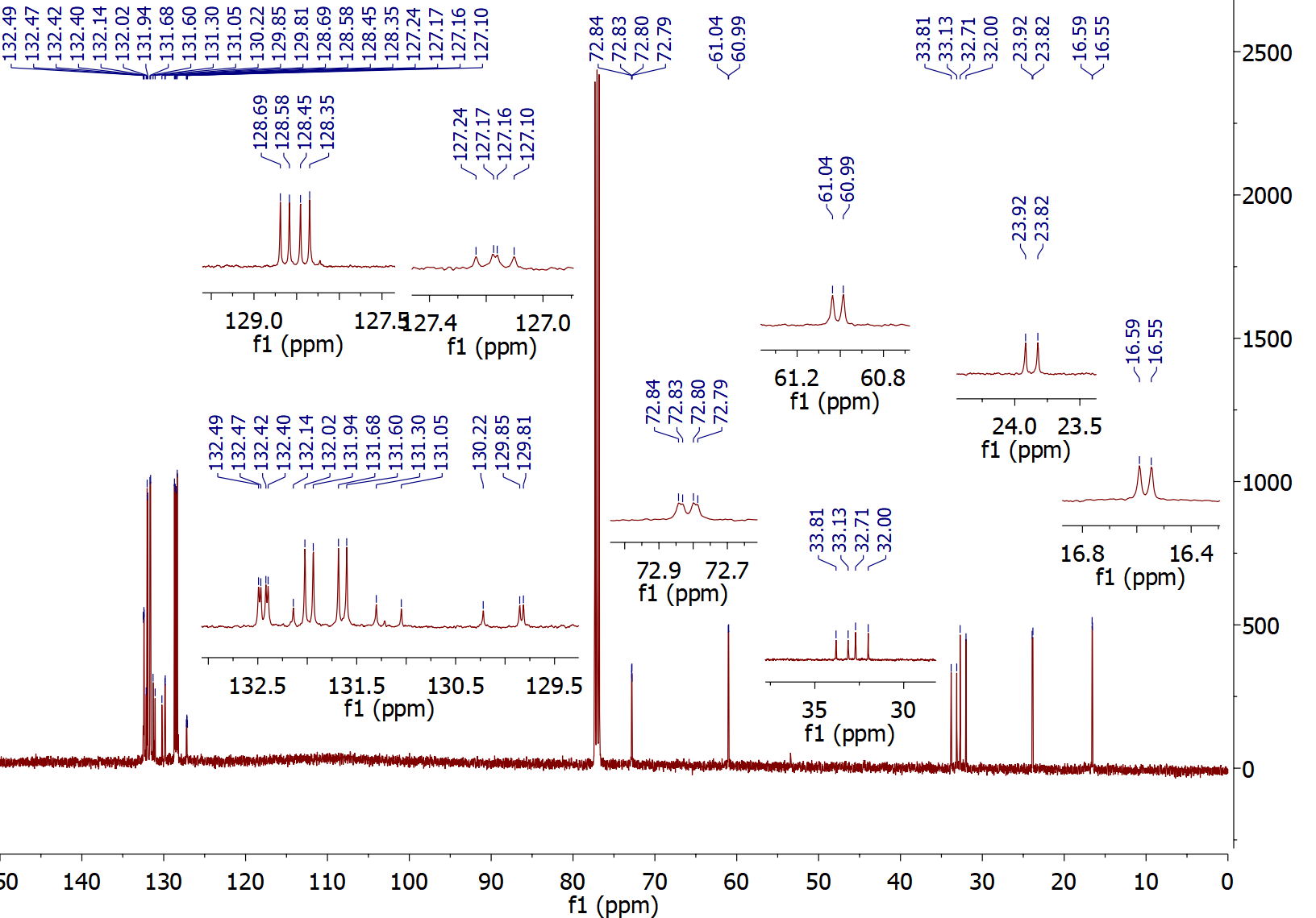


**Figure S31. ^1^H NMR (500 MHz, CDCl_3_) spectra for compound 4Ab**


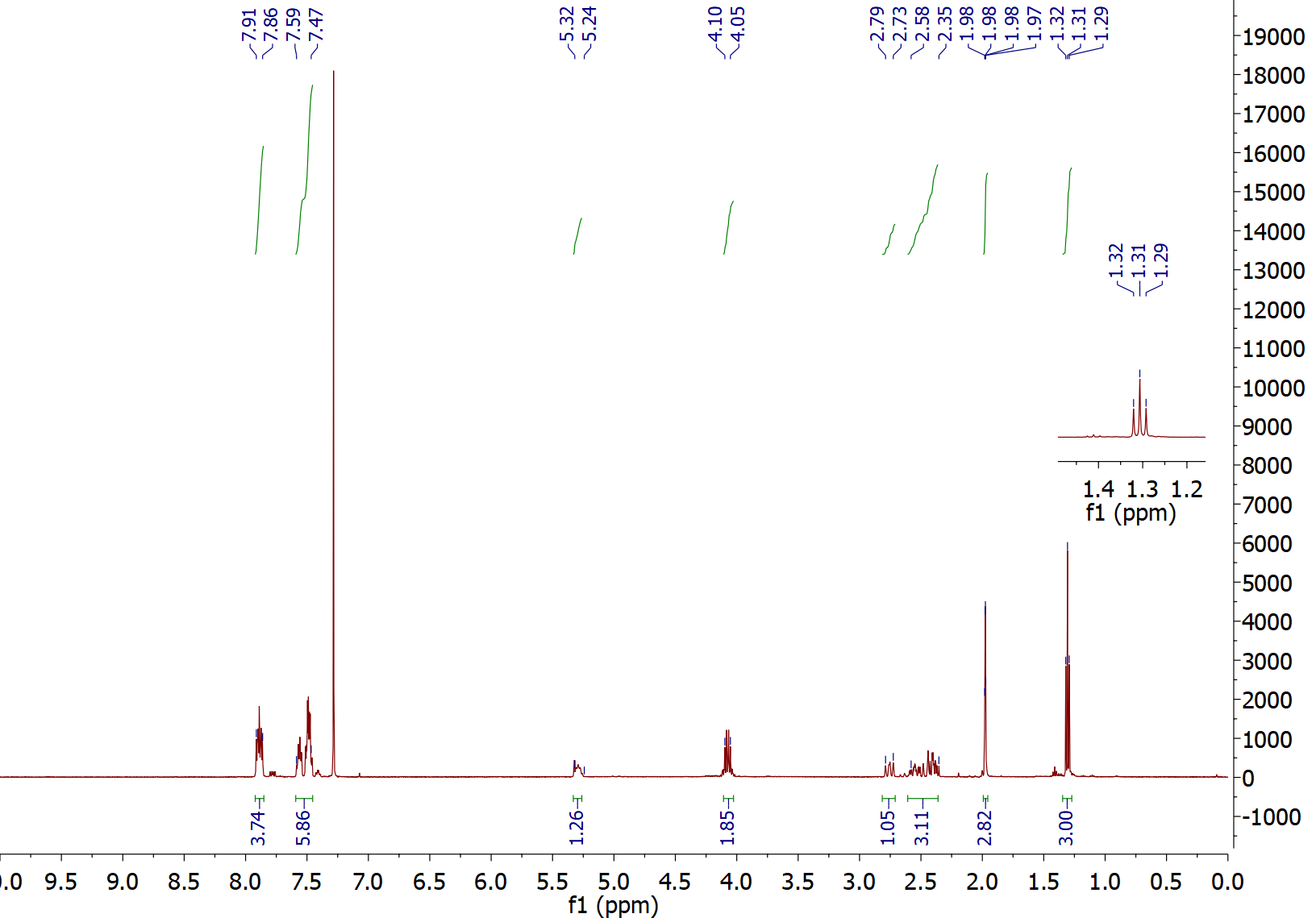


**Figure S32. ^31^P {^1^H} NMR (122 MHz, CDCl_3_) spectra for compound 4Ac**


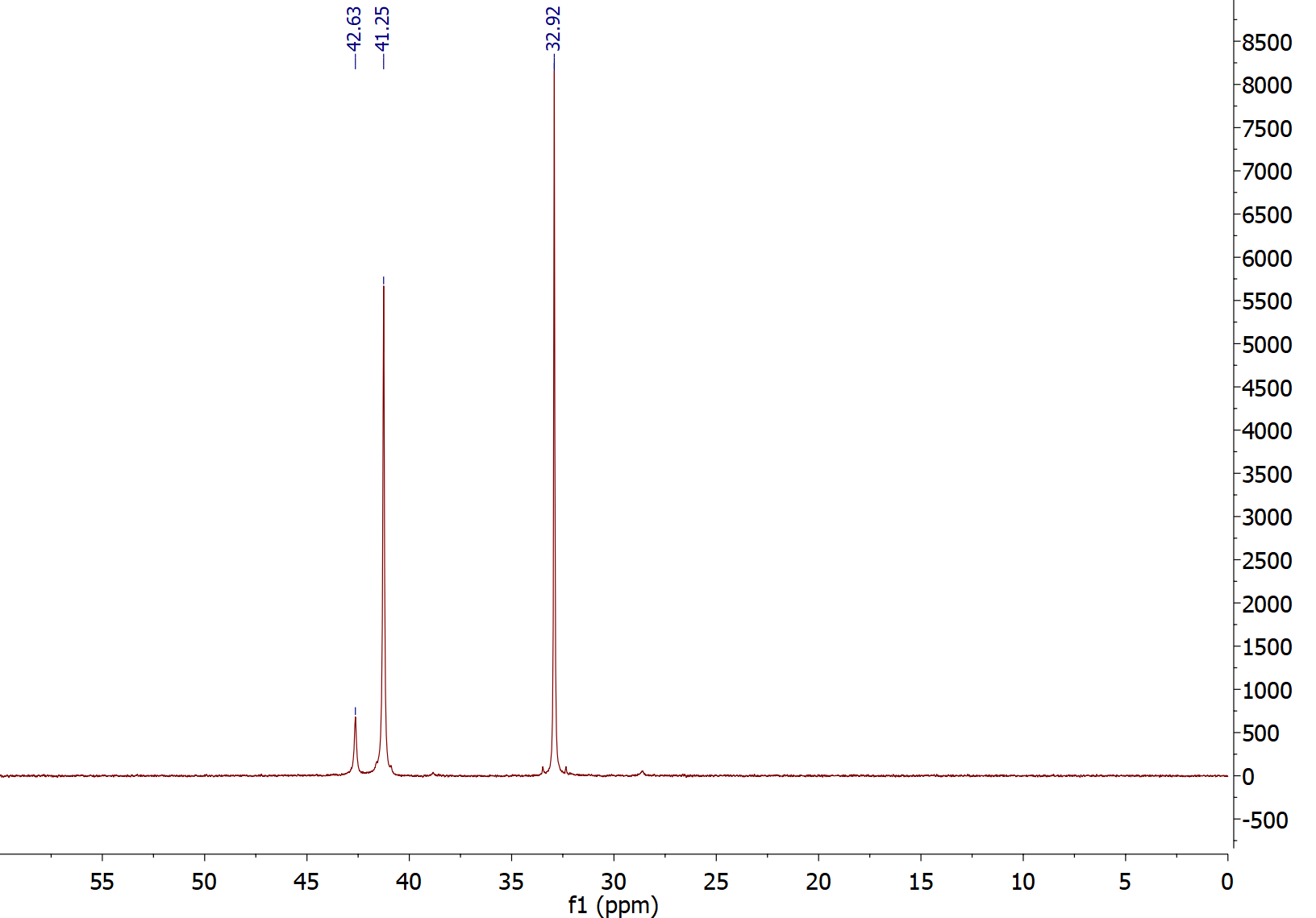


**Figure S33. ^13^C {^1^H} NMR (75 MHz, CDCl_3_) spectra for compound 4Ac**


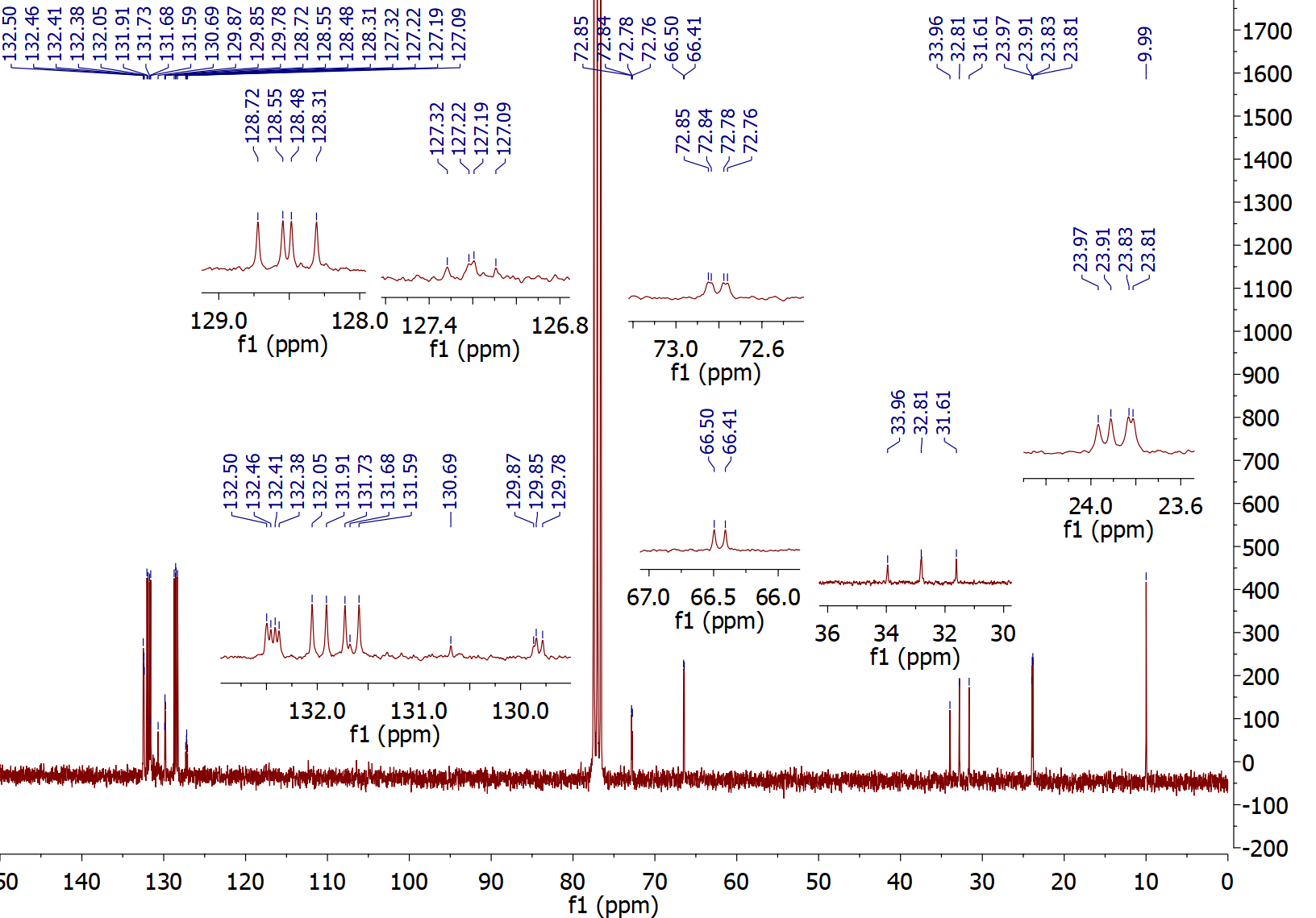


**Figure S34. ^1^H NMR (300 MHz, CDCl_3_) spectra for compound 4Ac**


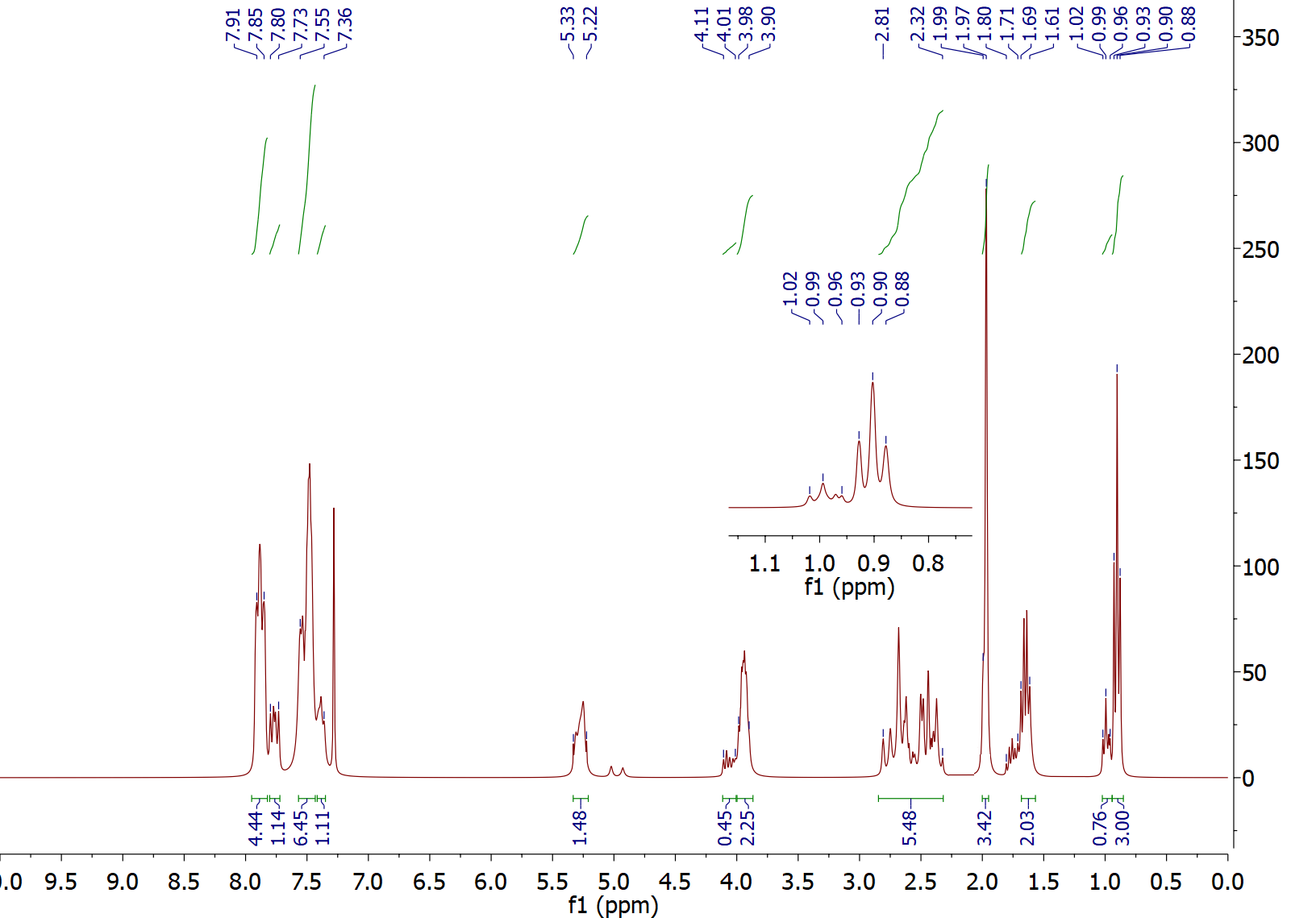


**Figure S35. ^31^P {^1^H} NMR (122 MHz, CDCl_3_) spectra for compound 4Ad**


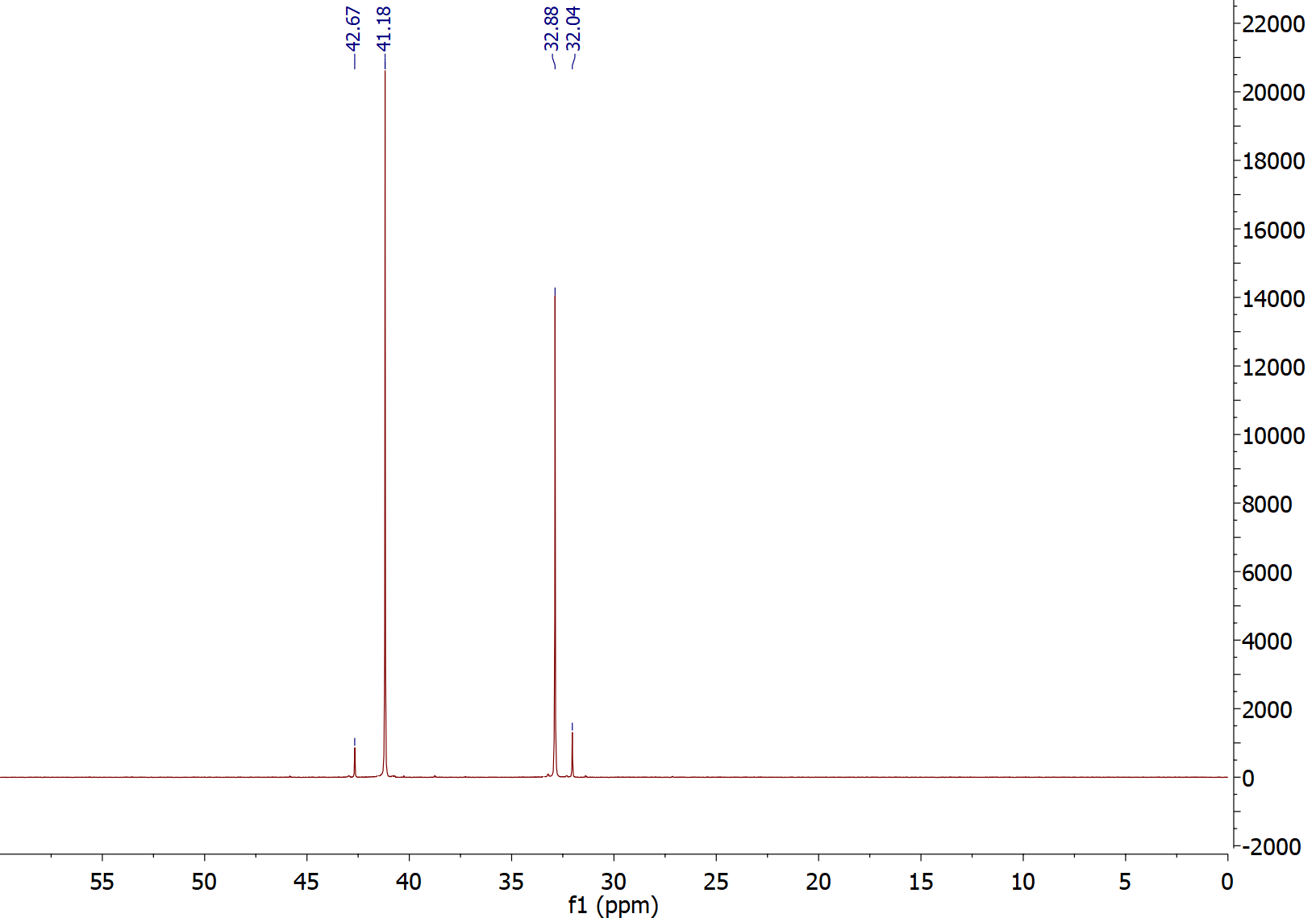


**Figure S36. ^13^C {^1^H} NMR (75 MHz, CDCl_3_-MeOH 95:5) spectra for compound 4Ad**


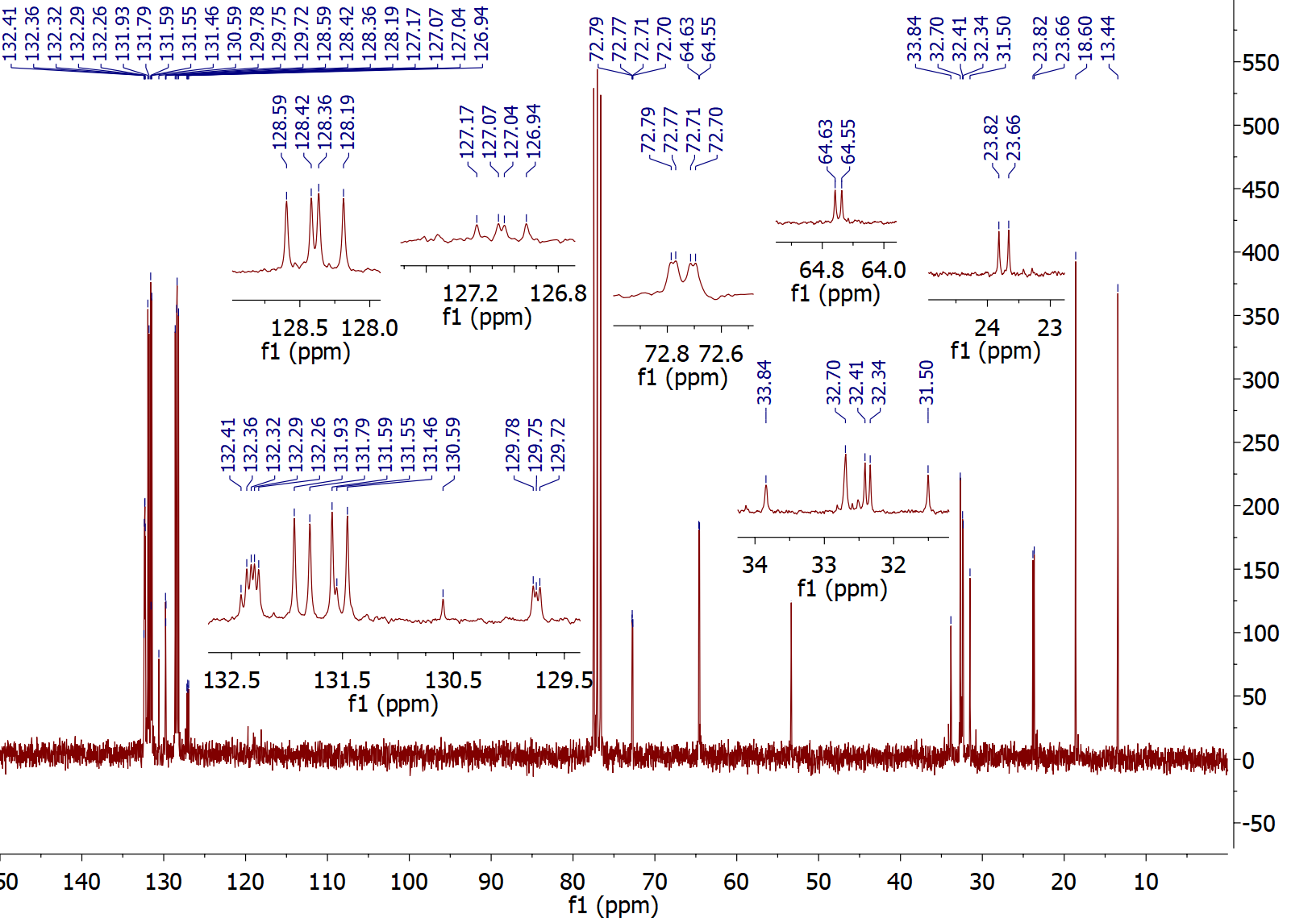


**Figure S37. ^1^H NMR (300 MHz, CDCl_3_-MeOH 95:5) spectra for compound 4Ad**


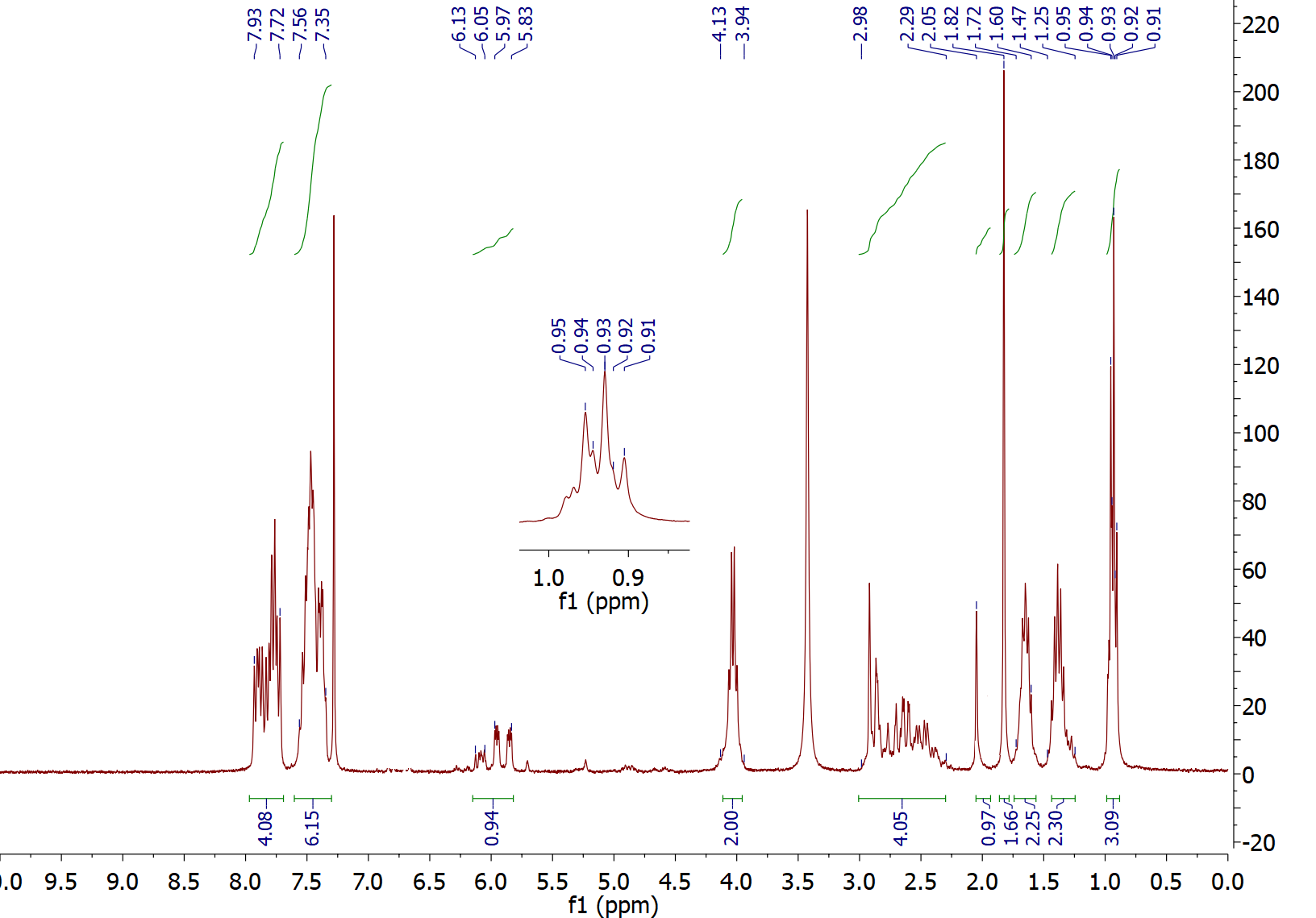


**Figure S38. ^31^P {^1^H} NMR (122 MHz, CDCl_3_) spectra for compound 4Ae**


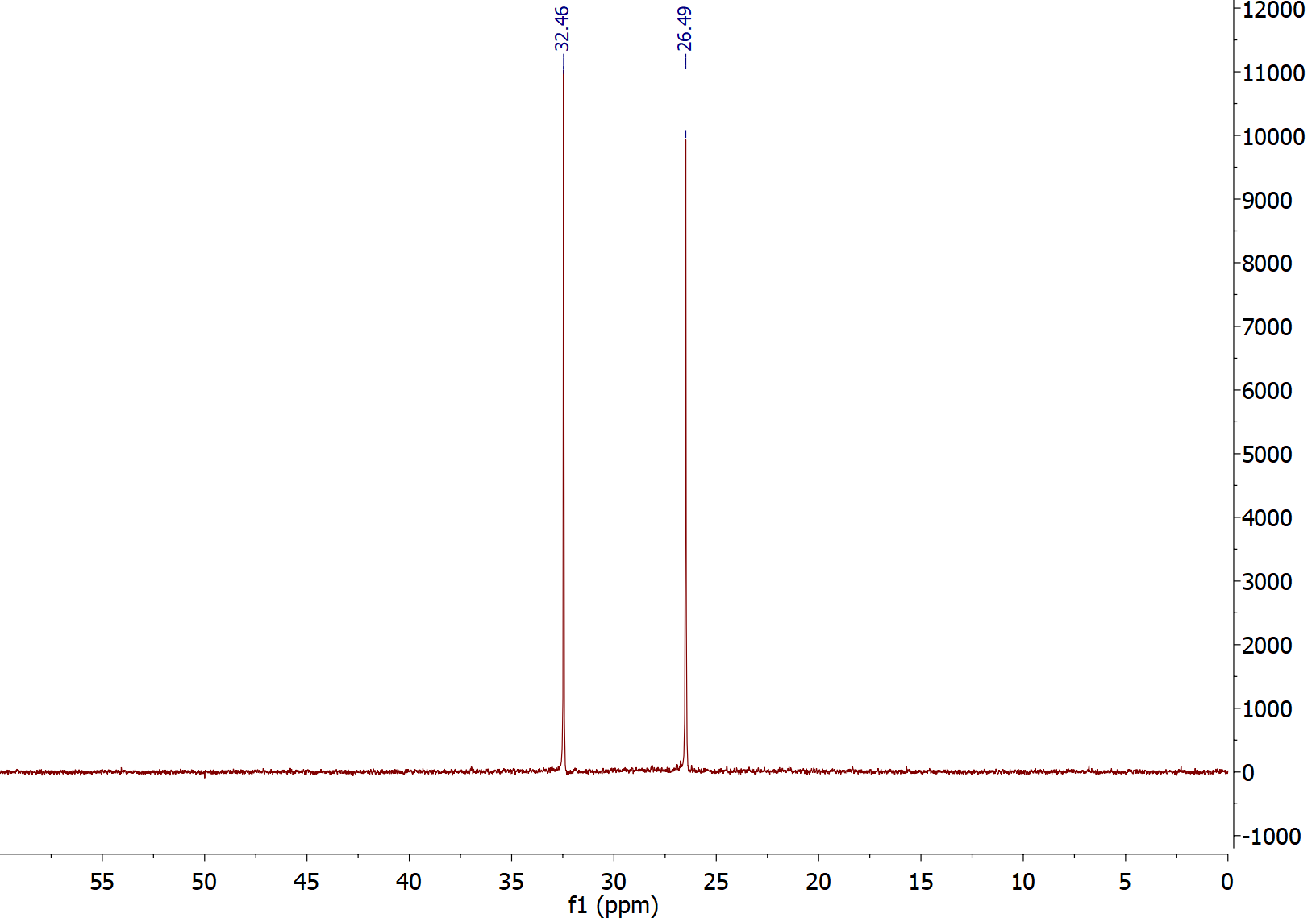


**Figure S39. ^13^C {^1^H} NMR (75 MHz, CDCl_3_) spectra for compound 4Ae**


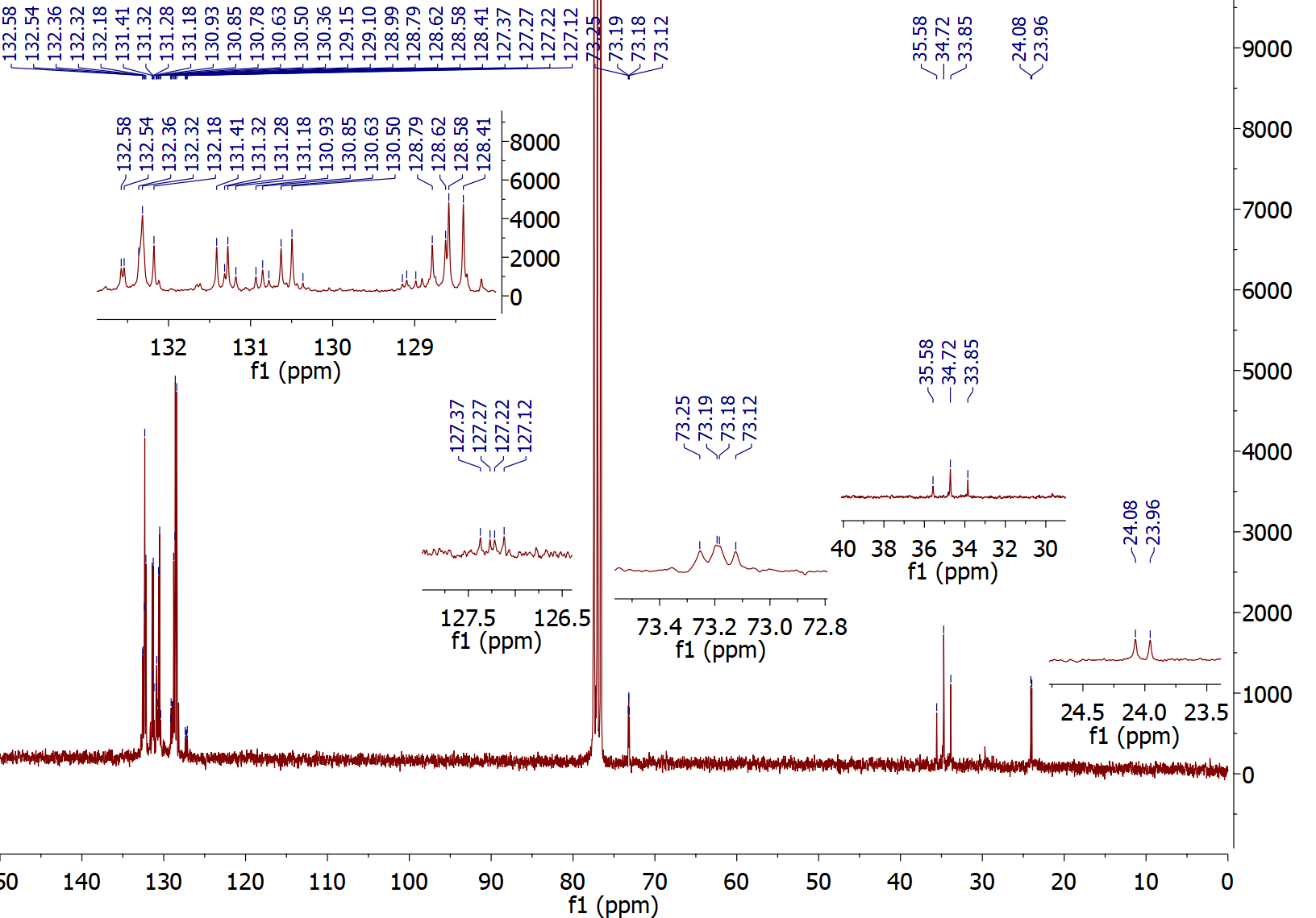


**Figure S40. ^1^H NMR (300 MHz, CDCl_3_) spectra for compound 4Ae**


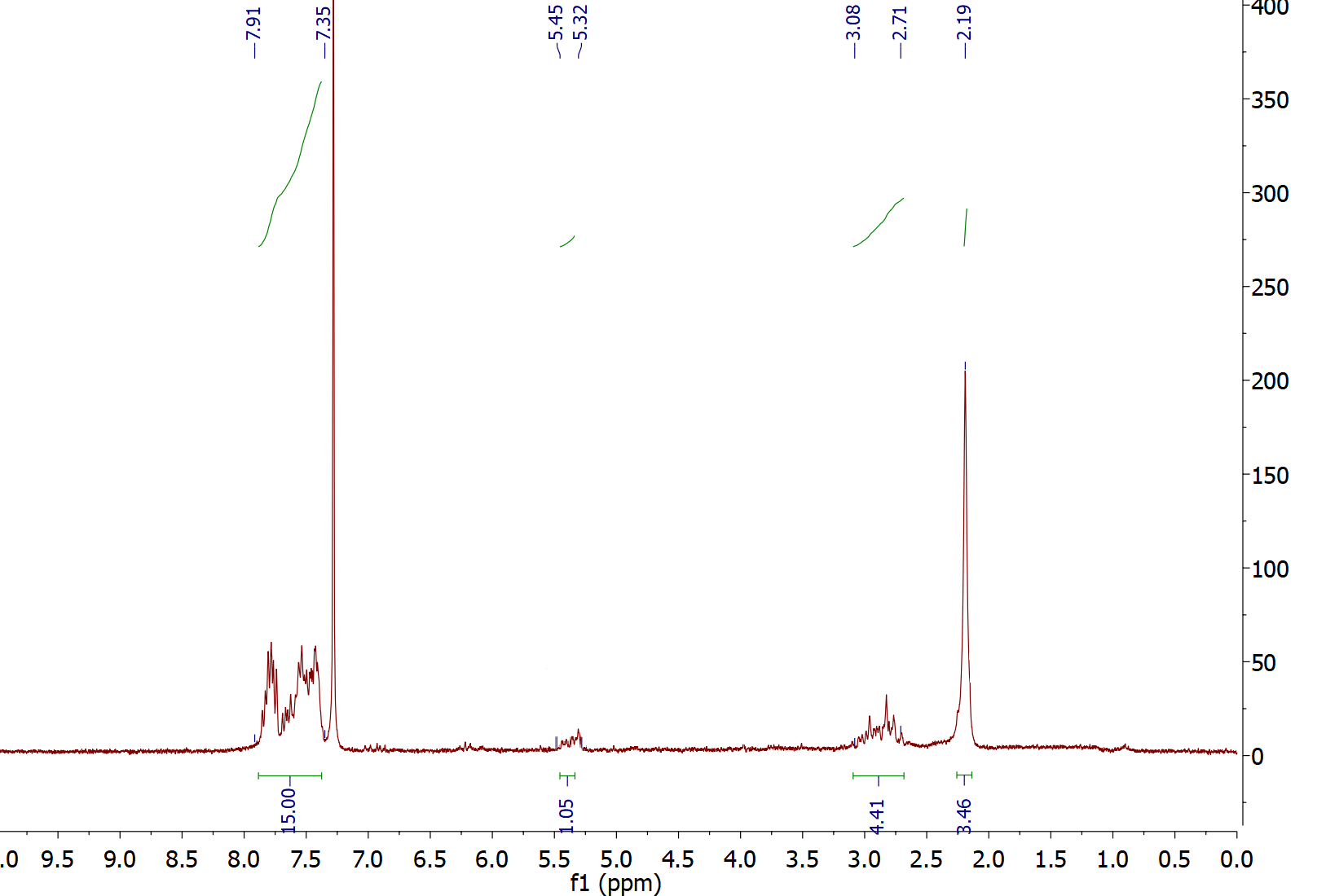


**Figure S41. ^31^P {^1^H} NMR (202 MHz, CDCl_3_) spectra for compound 5Ab**


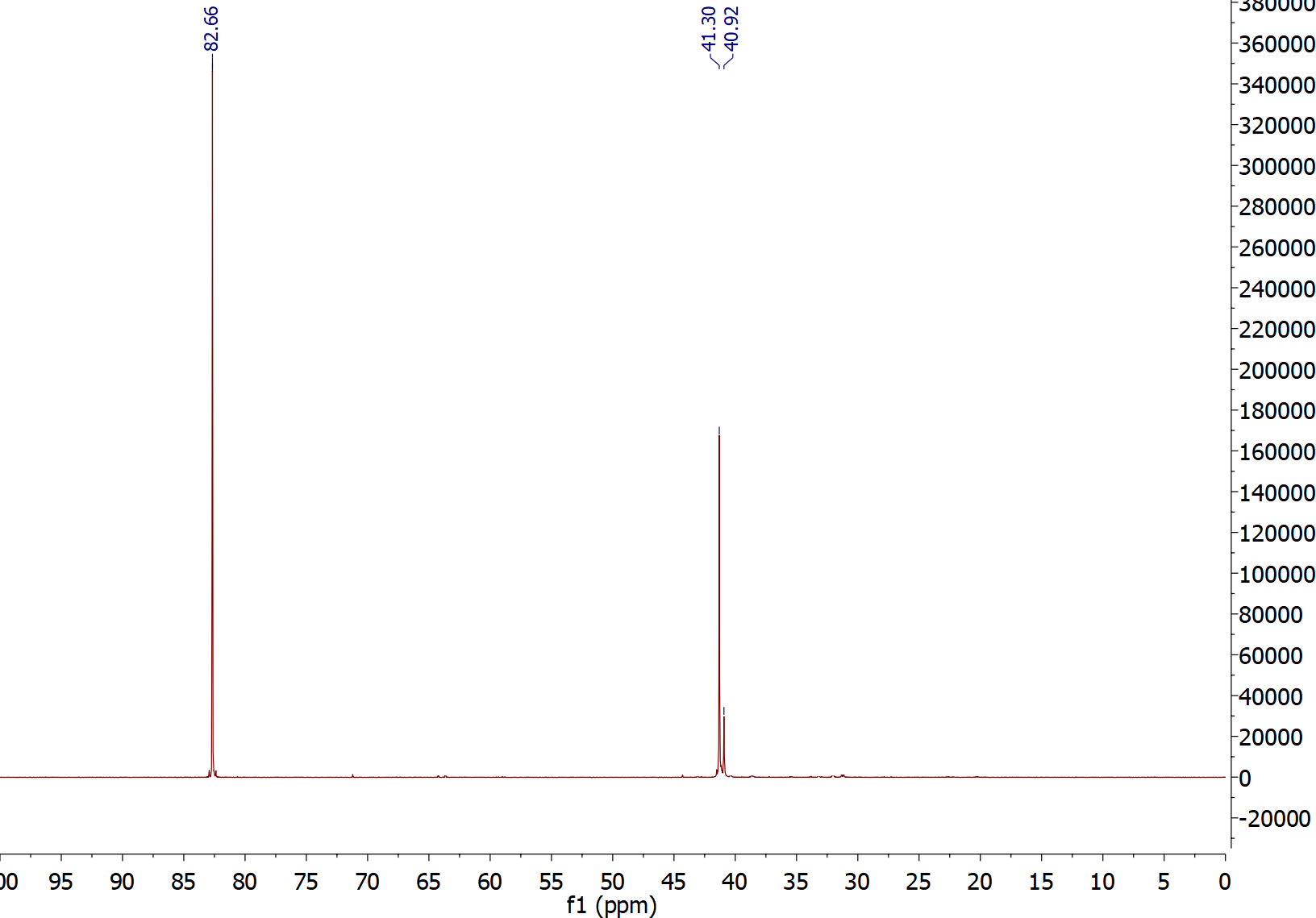


**Figure S42. ^13^C {^1^H} NMR (126 MHz, CDCl_3_) spectra for compound 5Ab**


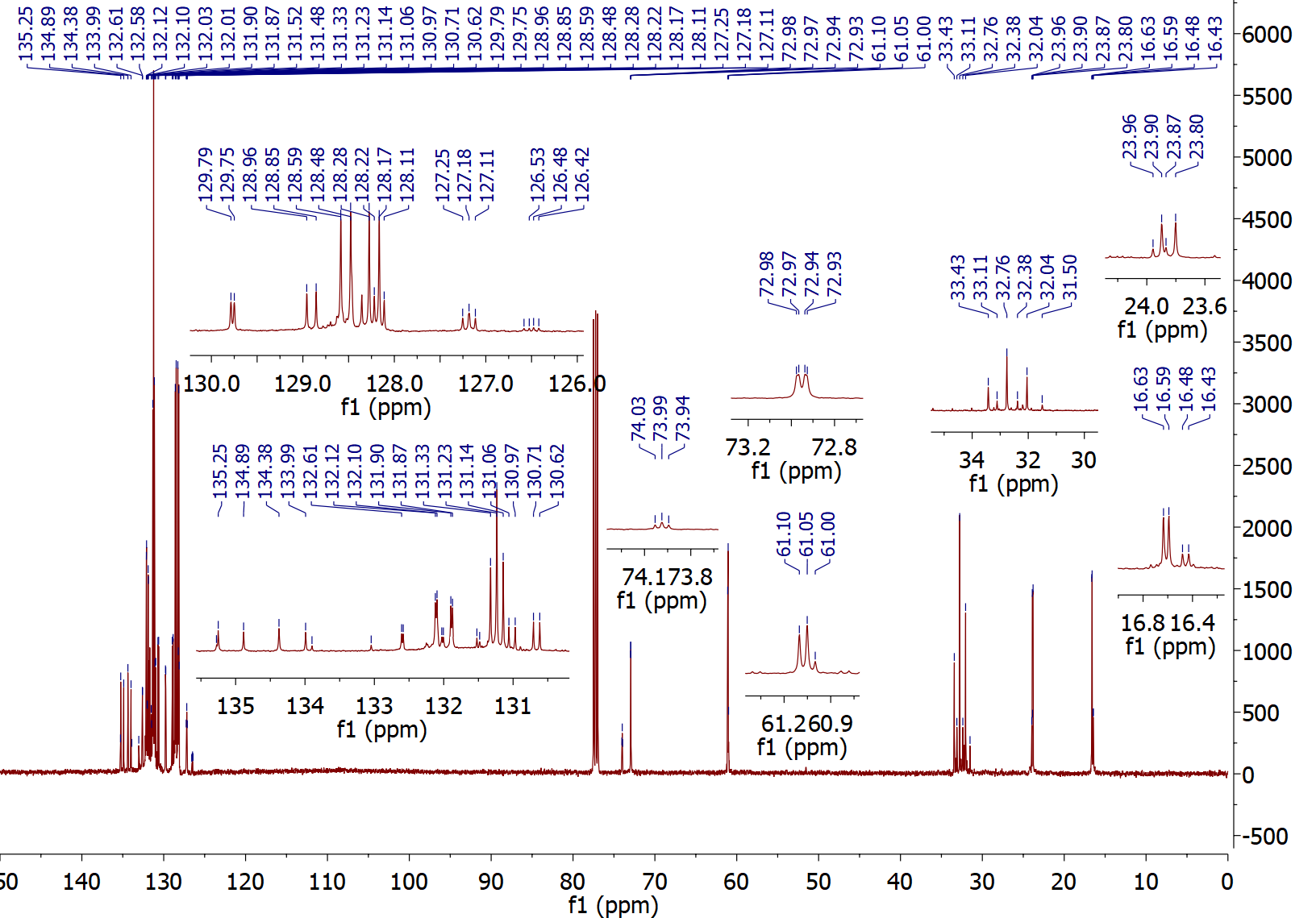


**Figure S43. ^1^H NMR (500 MHz, CDCl_3_) spectra for compound 5Ab**


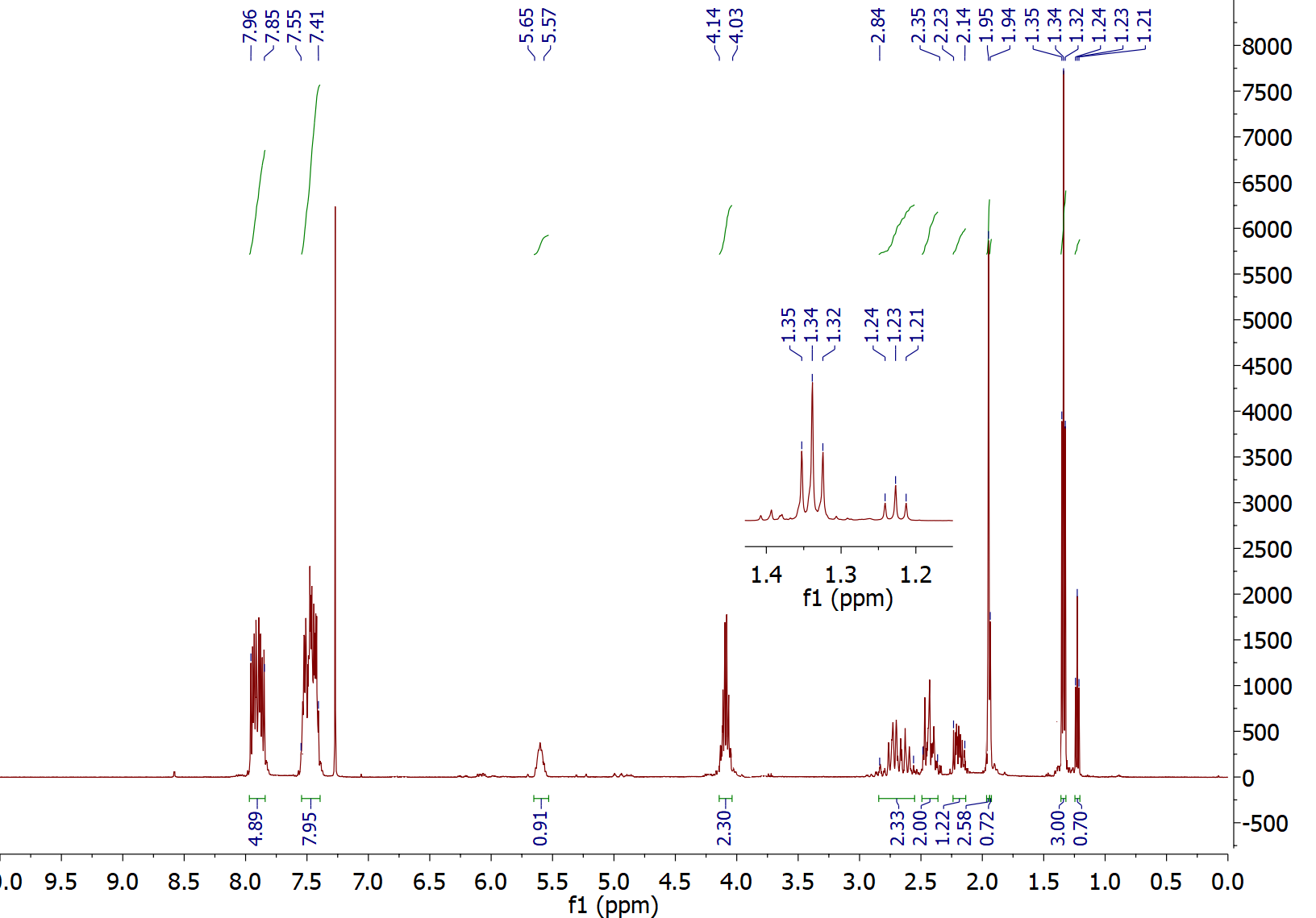


**Figure S44. ^31^P {^1^H} NMR (122 MHz, CDCl_3_) spectra for compound 5Ac**


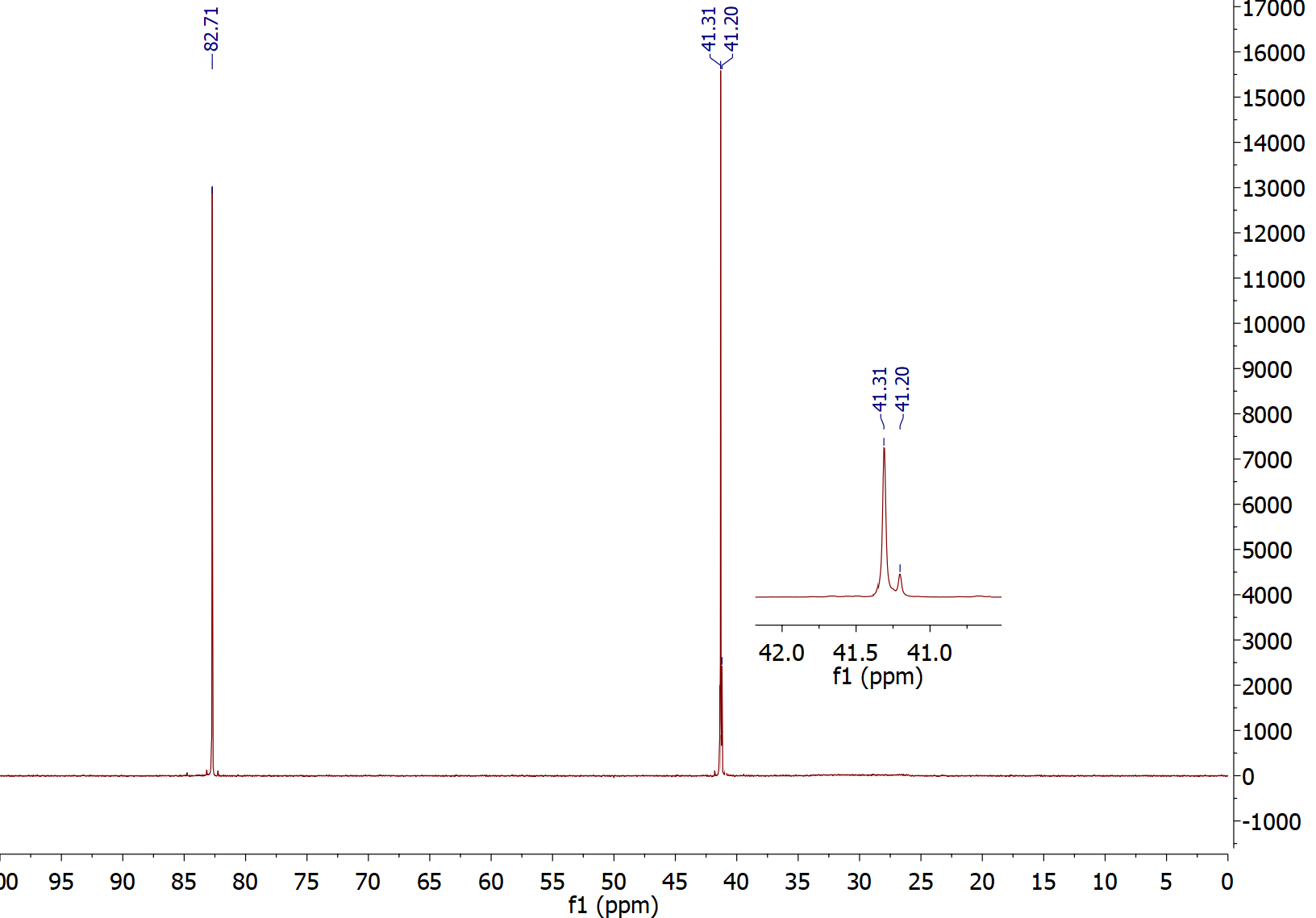


**Figure S45. ^13^C {^1^H} NMR (75 MHz, CDCl_3_) spectra for compound 5Ac**


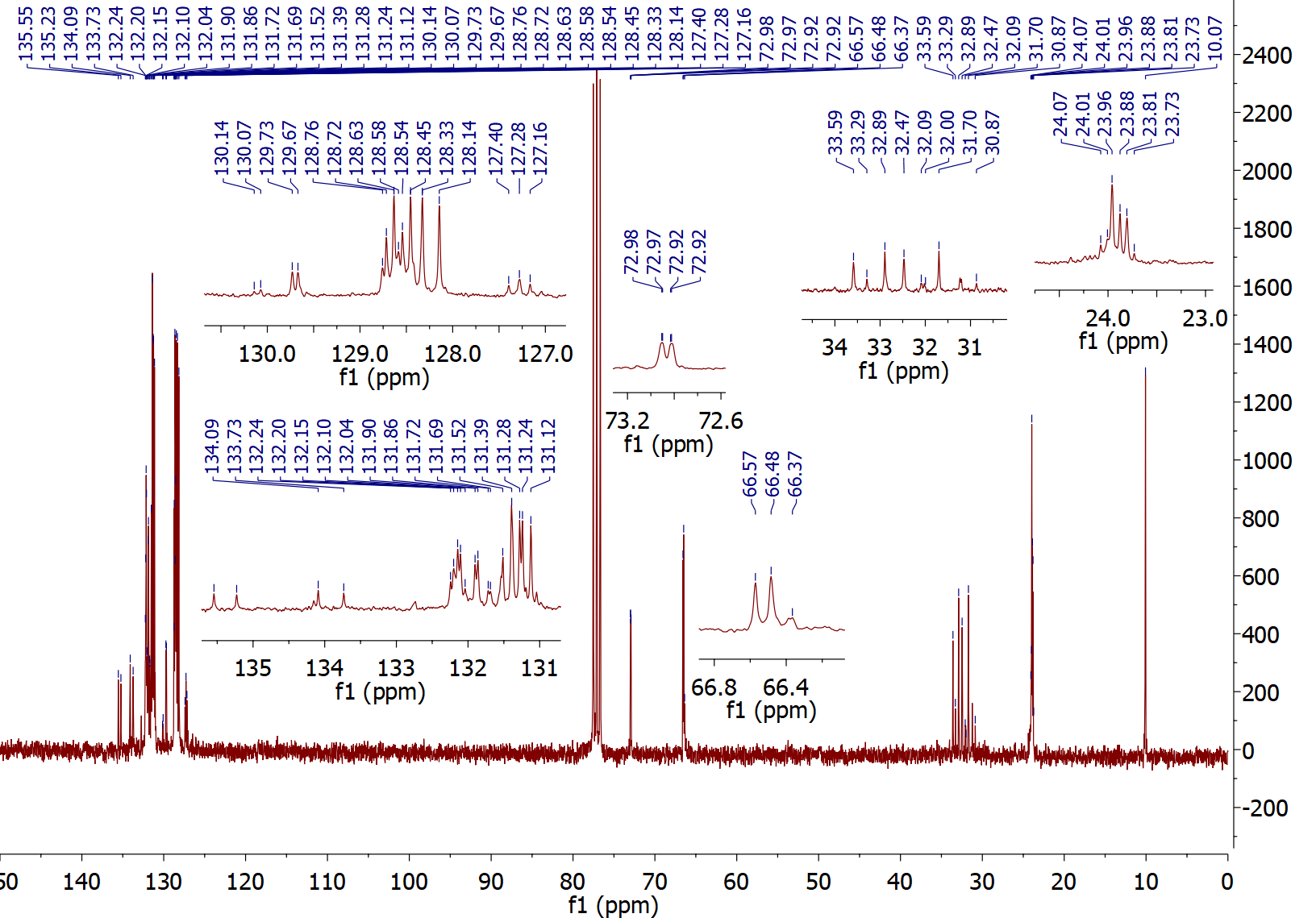


**Figure S46. ^1^H NMR (300 MHz, CDCl_3_) spectra for compound 5Ac**


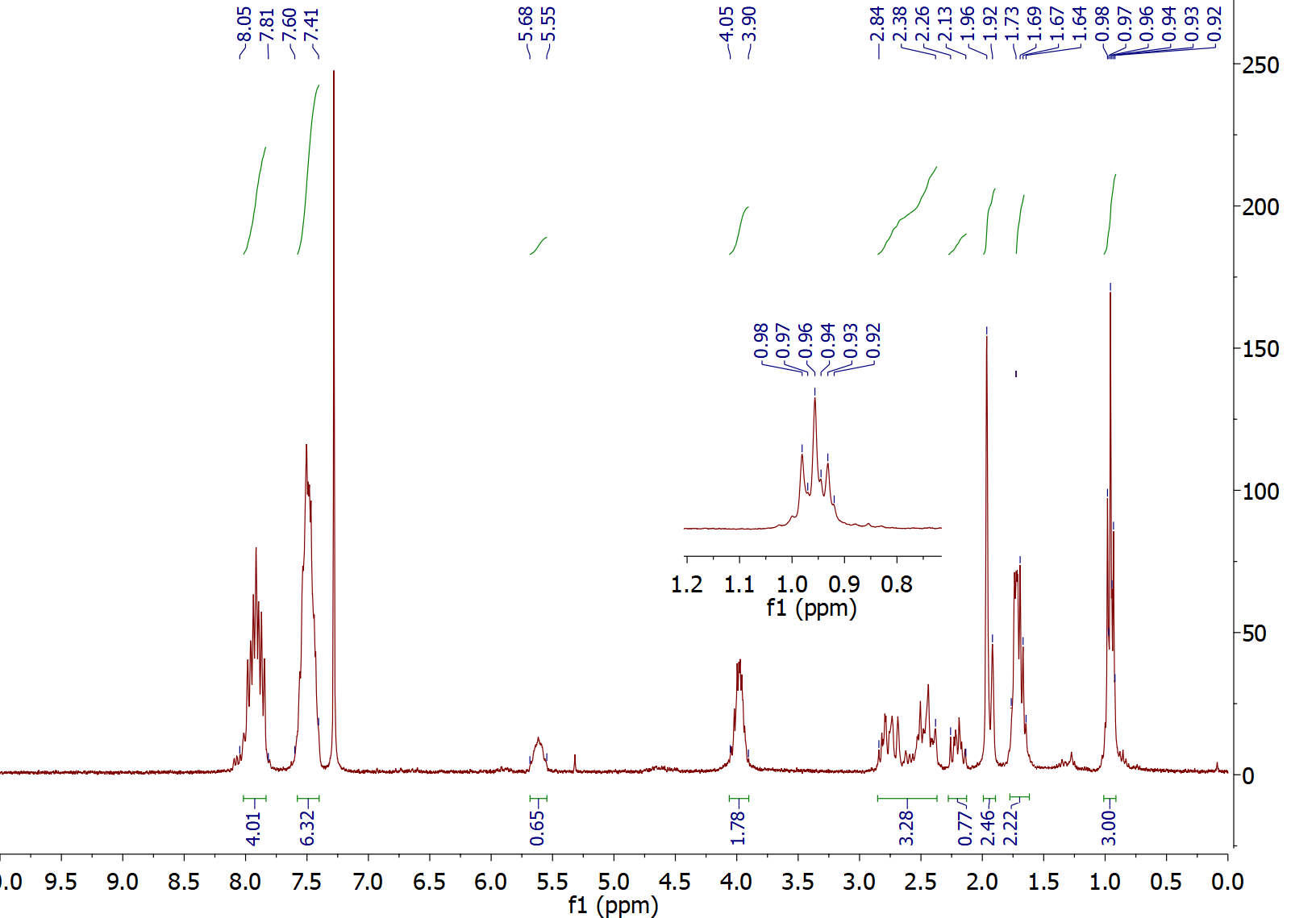


**Figure S47. ^31^P {^1^H} NMR (122 MHz, CDCl_3_) spectra for compound 5Ae**


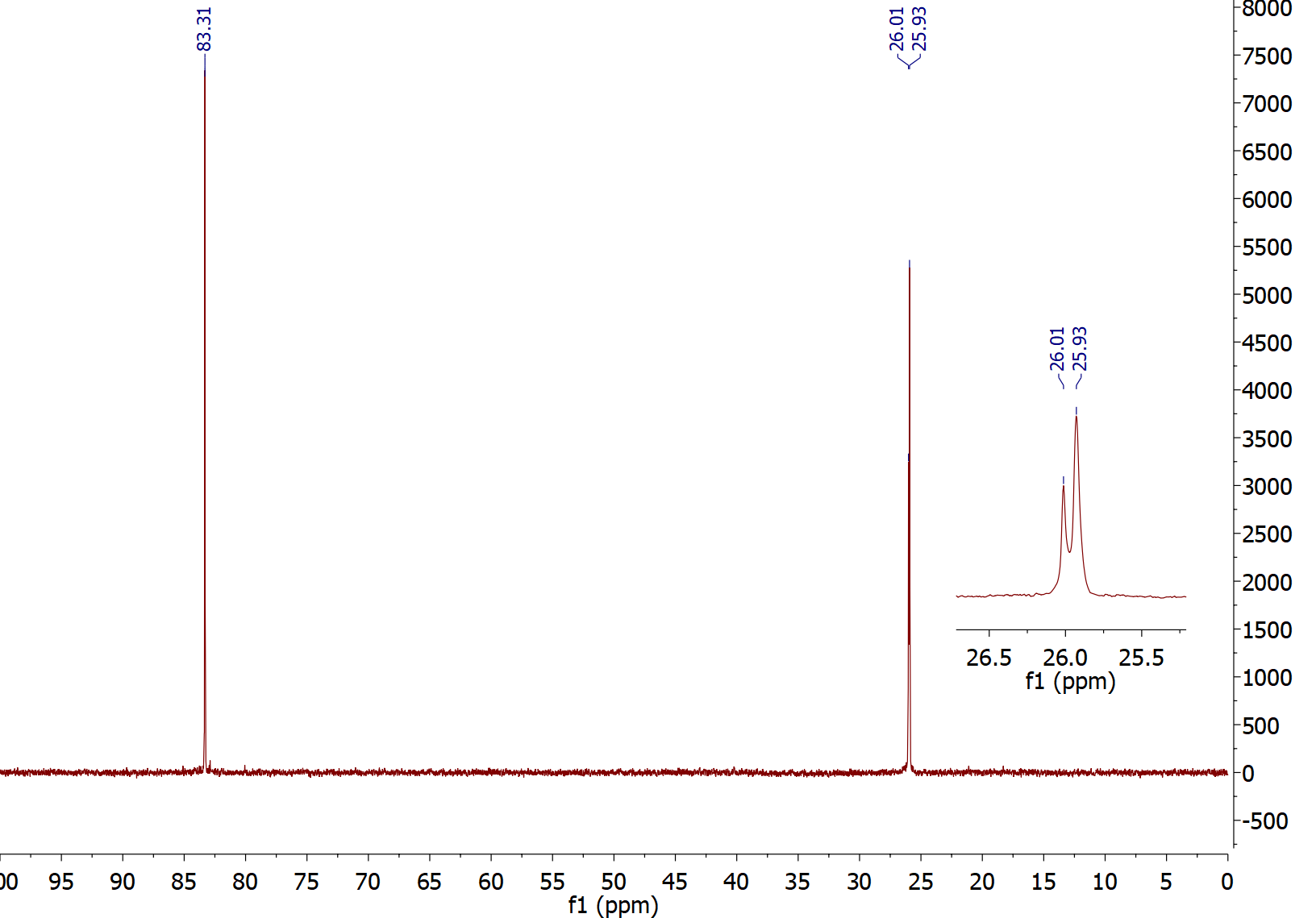


**Figure S48. ^13^C {^1^H} NMR (75 MHz, CDCl_3_) spectra for compound 5Ae**

**
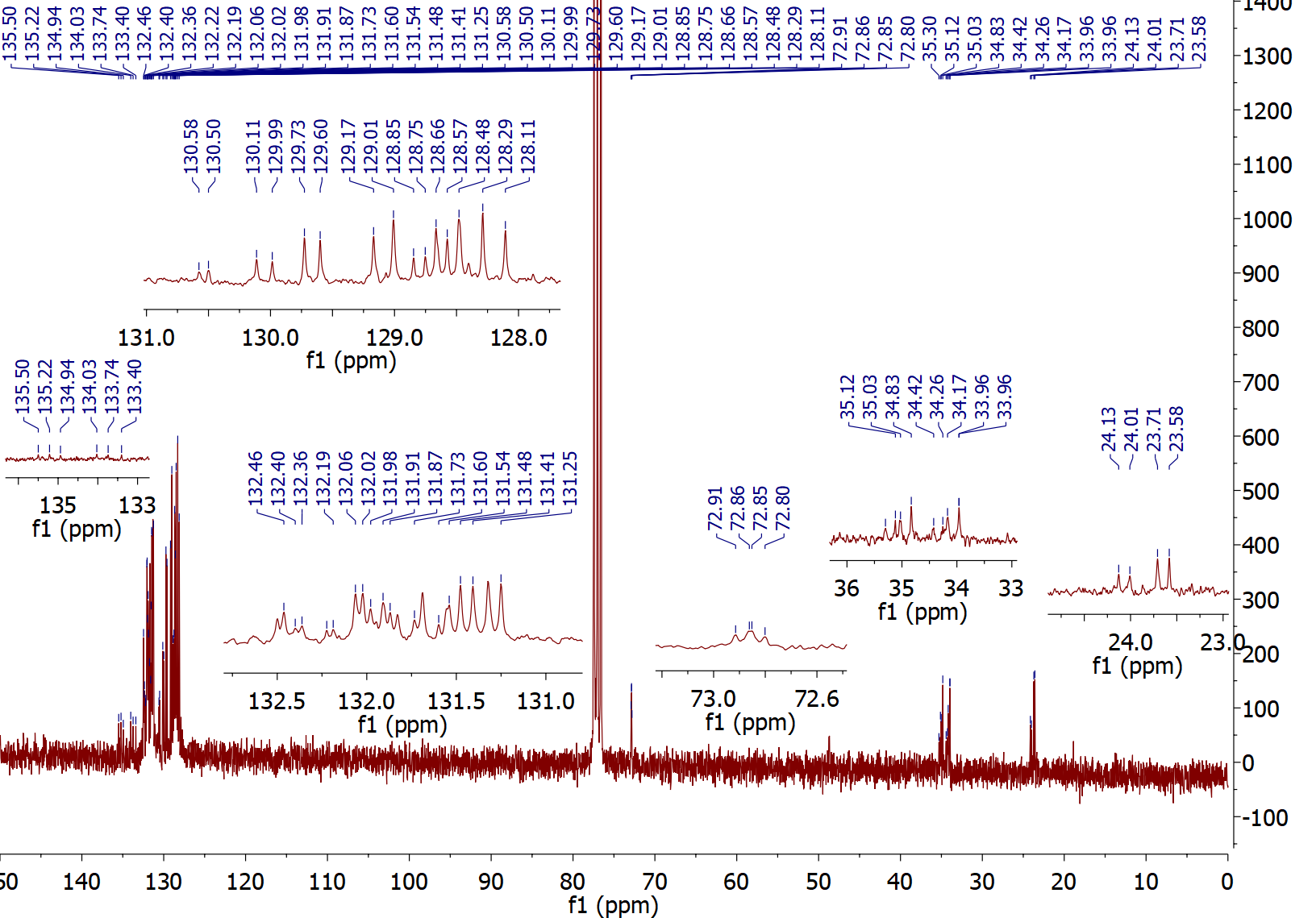
**

**Figure S49. ^1^H NMR (300 MHz, CDCl_3_) spectra for compound 5Ae**


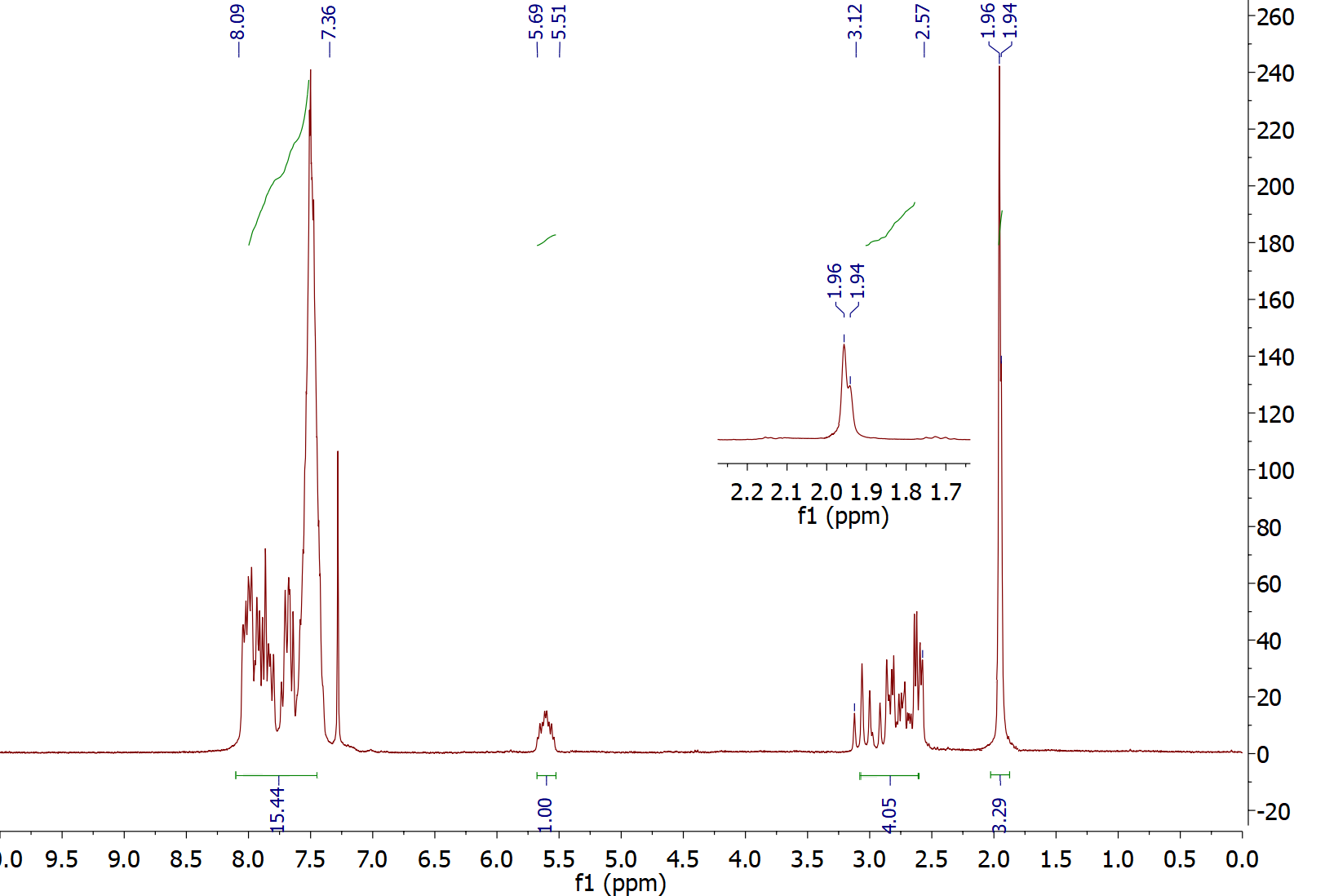

Supplement: Supplementary file 1 [file ao5c11546_si_003.docx]
